# Local Coordination Disorder and Defect Formation in Cadmium Zeolitic Imidazolate Framework Glasses

**DOI:** 10.1021/acs.inorgchem.6c03271

**Published:** 2026-09-24

**Authors:** Renato Pereira Murback Filho, Bruno Trebbi, Ricardo José da Silva Afonso, Camila Mizuki Ito Nishida, Jonas Lins, Gerd Buntkowsky, Torsten Gutmann, Eduardo Ribeiro deAzevedo, Marcos de Oliveira Junior

**Affiliations:** † Sao Carlos Institute of Physics, University of Sao Paulo, IFSC − USP, 13566-590 Sao Carlos, SP, Brazil; ‡ Eduard-Zintl-Institute of Inorganic and Physical Chemistry, 26536 Technical University of Darmstadt, Peter-Grünberg-Straße 8, Darmstadt D-64287, Germany; § Department of Chemistry, Physical Chemistry, University Paderborn, Warburger Straße 100, Paderborn D-33098, Germany

## Abstract

Cadmium-based zeolitic imidazolate glasses provide an attractive model system for investigating local coordination changes during vitrification. Here, we examine the structure of Cd­(Im)_2–*x*_(bIm)_*x*_ (Im = imidazolate, bIm = benzimidazolate) in its as-synthesized and glassy states using solid-state NMR spectroscopy, differential scanning calorimetry, electron paramagnetic resonance, and complementary techniques. Increasing bIm content reduces crystallinity and suppresses recrystallization, promoting glass formation. While ^13^C and ^1^H NMR spectra indicate largely preserved linker environments, broadening of ^15^N and ^113^Cd resonances reveals increased local disorder. ^113^Cd­{^1^H} REDOR measurements show that the average ^113^Cd–^1^H dipolar interaction is largely preserved upon vitrification. Thus, the predominant local Cd coordination environment is retained, although minor coordination defects cannot be excluded. EPR reveals thermally induced paramagnetic centers, whose microscopic origin remains unassigned and may include coordination-related defects and thermal decomposition. Variable-temperature ^1^H NMR further reveals dynamic changes across the glass-transition region. Overall, ligand chemistry influences melting, recrystallization, and glass formation in hybrid coordination networks. Vitrification combines the preservation of the predominant local Cd environment with an increase in local geometric disorder and the formation of thermally induced paramagnetic centers.

## Graphical abstract

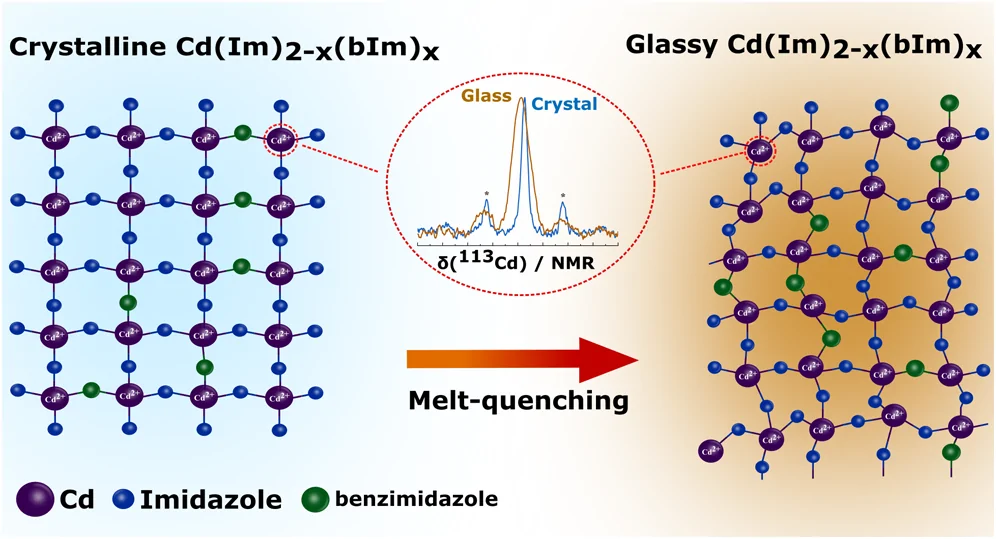

## Introduction

Metal–organic frameworks (MOFs) are a class of porous materials synthesized from inorganic clusters and organic ligands, allowing the formation of well-defined structures with controlled pore size and crystallinity. Different combinations of building elements (metal ions and linkers) make it possible to vary the flexibility of the metal–organic structures and the shape of the cavities, as well as the size of the pores and the degree of crystallinity.^1^ This chemical and structural versatility has led to a vast variety of compounds (over 160 thousand, according to the *Chemoinformatics database for Metal Organic Frameworks* ^2^) for multiple applications, such as gas storage^3^ and gas separation,^4^ catalysis,^5^ photonics,^6–8^ separation of pollutants in aqueous media,^9^ drug delivery,^10^ among others. Although most reported MOFs are crystalline, this is not mandatory for most of their interesting properties.^11^ In the past decade there has been an increasing interest in the development of amorphous MOFs (a-MOFs) materials, especially the glass-forming ones (a_g_-MOFs). These materials offer high stability, large surface area, excellent porosity, and tunable features of traditional MOFs, combined with properties of the amorphous domain like isotropy, defect abundance, active sites, and moldability.^12^ With their demonstrated glass-forming capabilities, Metal–Organic Frameworks (MOFs) glasses have expanded the traditional classification of vitreous materials beyond metallic, organic, and inorganic systems.^13^

Zeolitic imidazolate frameworks (ZIFs) represent a subclass of MOFs, characterized by their transition metal–imidazolate linkages and 145° bond angles, similar to those found in traditional zeolites (e.g., SiO_2_).^14^ Their crystalline structure can be collapsed via different pathways, including melt-quenching,^15^ high-temperature solid–solid transitions,^12,16–18^ high-pressure amorphization,^19^ and ball milling,^20–23^ resulting in amorphous structures. Pair distribution function (PDF) analysis has revealed these structures to be continuous random networks of corner-sharing Zn­(ImT)_2_ (where ImT refers to imidazolate-based organic ligands in general) tetrahedral structural elements, analogous to models of amorphous SiO_2_.^11,12,24,25^ These amorphous materials are of particular interest due to their distinct chemical, mechanical, and physical properties compared with their crystalline counterparts, as well as the ability to trap guest molecules within the collapsed structures.^26,27^

As a newly recognized class of glasses, fundamental questions remain regarding the atomic-level structure of MOF glasses. Moreover, due to the lack of long-range periodicity, these materials represent a major challenge for the complete understanding of their structure. Previous research^13^ has indicated that short-range order is maintained during the transition of ZIF crystals into the glassy phase. However, Madsen et al.^28^ recently reported evidence of short-range disorder in the zinc coordination environment, observed via ultrahigh field ^67^Zn solid-state Nuclear Magnetic Resonance (NMR) spectroscopy. NMR is very well suited for the structural investigation of these glasses, providing valuable information which is complementary to total scattering experiments.^15,27–32^ However, for certain isotopes, solid-state NMR may not be practical for obtaining detailed structural information. For example, the ^67^Zn isotope presents challenges due to the low natural abundance (approximately 4.1%) and a moderate quadrupolar moment, which broadens spectral lines (especially for amorphous systems) and complicates interpretation. While ultrahigh fields can mitigate these issues, their limited accessibility remains a constraint.^28^

To investigate the short-range structural disorder around the metal sites of MOF glasses, we selected Cd^2+^ as a model cation because of its favorable NMR properties. Unlike ^67^Zn, ^113^Cd has a relatively higher natural abundance and a nuclear spin of 1/2, making it particularly suitable for probing local coordination environments by solid-state NMR. Recently, Xue et al.^33^ established the melting, vitrification, and recrystallization behavior of the Cd­(Im)_2–*x*_(bIm)_*x*_ family prepared by a mechanochemical route, demonstrating that partial substitution of imidazolate by benzimidazolate suppresses recrystallization and stabilizes the glassy state. Their study further showed, through pair distribution function (PDF) analysis, that the first coordination shell around Cd is largely preserved after vitrification, while highlighting the lower melting and glass transition temperatures of the Cd-based ZIFs with their Zn analogues. These findings established the Cd­(Im)_2–*x*_(bIm)_*x*_ system as an important extension of MOF-glasses chemistry beyond the widely studied Zn-based ZIFs.

Building upon these results, we investigate the same compositional series synthesized by a mild room-temperature solution-precipitation route.^34^ This synthetic approach provides an alternative route to the preparation of Cd­(Im)_2–*x*_(bIm)_*x*_ (*x* = 0 to 0.40) frameworks and their corresponding melt-quenched glasses. More importantly, the present work provides a comprehensive multinuclear solid-state NMR investigation of the local structural changes accompanying vitrification. The combination of ^1^H, ^13^C, ^15^N, ^113^Cd, and ^113^Cd­{^1^H} REDOR experiments directly probes the local chemical environments of both the Cd centers and the organic linkers, providing complementary insight into local coordination disorder and thermally generated paramagnetic defects. We further demonstrate the application of Dipolar-Filtered Magic Sandwich Echo (DF-MSE) pulse sequences to time-domain ^1^H NMR cryoporometry,^35^ providing a reproducible approach to monitor the evolution of textural mesoporosity with vitrification. To capture the underlying structural relaxation and thermal dynamics during the liquid-to-glass transitions, we deploy in situ variable-temperature static ^1^H Hahn-echo NMR spectroscopy, mapping the onset of liquid-like translational dynamics across the *T*_g_ region. Together, these complementary techniques provide an atomistic description of the structural changes accompanying vitrification, revealing increased local coordination disorder while the predominant Cd coordination environment remains largely preserved.

## Experimental Section

### Synthesis of Cd­(Im)_2–*x*_(bIm)_*x*_

For *x* = 0 (dia-Cd­(Im)_2_), the synthesis was carried out following a previously reported procedure.^16^ In this case, methanol solutions of Cd­(NO_3_)_2_·4H_2_O (9.53 × 10^–5^ mol/mL) and imidazole (6.06 × 10^–4^ mol/mL) were mixed. For the deprotonation of imidazole and subsequent formation of the framework, an aqueous solution of NaOH (4.24 × 10^–3^ mol/mL) was added dropwise to the metal/ligand solution. After 5 min of stirring, a white precipitate was formed. The final solution was filtered under vacuum and the product was washed twice with 10 mL portions of methanol and twice with 10 mL portions of deionized water. The material was dried in a muffle furnace at 80 °C for 30 min.

For *x* > 0, the synthesis of the variable-ligand system was adapted from the dia-Cd­(Im)_2_ solution-precipitation route. To calculate the amounts of cadmium, imidazole and benzimidazole, we used a protocol already established for traditional ZIF-62.^36^ Cd­(NO_3_)_2_·4H_2_O (4.0 mmol) was added to 40 mL of methanol and the mixture was stirred for 1 h to form **solution A**. Ligands were dissolved separately in different ratios of imidazole and benzimidazole (molar ratios given in Table 1). Imidazole was added to 30 mL of methanol, and it was stirred for 30 min to form **solution B**, and benzimidazole was added to 15 mL of methanol and stirred for 20 min to form **solution C**. **Solution C** was added dropwise to **solution B** to form **solution B′** and was stirred for 10 min. Next, **solution B′** was added dropwise to **solution A**. The final solution was stirred for 1 h. An aqueous solution of NaOH (1.25 × 10^–2^ mol/mL) was added dropwise to the final solution to form a white precipitate. The final solution was filtered under vacuum, and the product was washed twice with 10 mL portions of methanol and twice with 10 mL portions of deionized water. The material was dried in a muffle furnace at 80 °C for 30 min. The samples were named based on the experimental Im^–^:bIm^–^ ratio obtained from liquid ^1^H NMR experiments. The nominal Im^–^:bIm^–^ ratio used in the synthesis are presented in Tables 1 and S2.

**Table 1:** Concentrations of Reagents Used in the Synthesis of Cd­(Im)_(2–*x*)_(bIm)_*x*_ Samples^a^

	Im^–^:bIm^–^ ratio					
Cd(Im)_(2–*x*)_(bIm)_*x*_	nominal	experimental	Him (mmol)	HbIm (mmol)	Him (mg)	HbIm (mg)
*x* = 0.02	87:1	99:1	13.04	0.15	888	18
*x* = 0.05	43:1	39:1	12.89	0.3	878	36
*x* = 0.22	14:1	8:1	12.34	0.88	840	104
*x* = 0.40	7:1	4:1	11.54	1.63	786	193

a Experimental Im^–^:bIm^–^ ratios were determined by ^1^H liquid-state NMR.

### Cd­(Im)_(2–*x*)_(bIm)_*x*_ Glasses Preparation

The as-synthesized Cd­(Im)_2*–x*_(bIm)_*x*_ samples were placed in a platinum crucible and melted using a furnace housed inside a glovebox under an inert N_2_ atmosphere. The specific melting point (*T*_m_) for each composition was previously determined via Differential Scanning Calorimetry (DSC) experiments. To maximize the cooling rate and ensure efficient quenching of the molten phases, the crucibles were immediately removed from the furnace upon reaching *T*_m_ and brought into direct contact with a metal plate precooled in liquid nitrogen. This melt-quenching procedure successfully yielded homogeneous, dark brown glassy materials.

### Powder X-ray Diffraction (PXRD)

Structural analysis of the crystalline phase of the samples was carried out in a Rigaku Ultima IV diffractometer using Cu–Kα radiation (λ = 1.5418 Å) in a Bragg–Brentano geometry, at 40 kV and 20 mA, equipped with a sealed copper tube. Diffraction patterns were obtained from 5 to 50° (2θ), step size of 0.02° and scan rate of 5°/min.

### ^1^H Liquid State NMR

Solution ^1^H NMR measurements were performed on acid-digested samples with Bruker DPX-400 spectrometer using DMSO-d6 (0.6 mL) and DCl/D2O (30 wt %, approximately 0.02 mL) as solvents. The data were processed using the ssNake software. The spectra were referenced to the residual proton signal of DMSO, and chemical shifts are given relative to tetramethylsilane.

### Fourier Transform Infrared Spectroscopy (FTIR)

FTIR analyses were performed to identify the functional groups present in the samples using a PerkinElmer Frontier FTIR spectrometer operating in Attenuated Total Reflectance (ATR) mode. Spectra were acquired in the 4000–650 cm^–1^ range. For measurements, powdered samples were applied directly to the diamond crystal.

### Differential Scanning Calorimetry (DSC)

The thermal behavior of the samples was investigated by Differential Scanning Calorimetry (DSC) using a Netzsch DSC 204 Phoenix instrument. Prior to the measurements, the materials were dried overnight at 90 °C under vacuum and subsequently sealed in alumina crucibles inside a glovebox under an inert N_2_ atmosphere. The DSC scans were recorded under a continuous N_2_ flow of 20 mL/min, using a constant heating rate of 10 °C/min. Prior to the DSC measurements, a preliminary heat treatment was performed at 300 °C to ensure the desolvation of molecules trapped in the pores and to allow for structural relaxation processes.

### Thermogravimetric Analysis (TGA)

The thermal stability of the samples was evaluated by thermogravimetric analysis (TGA) using a Shimadzu TGA-50 Thermobalance. Approximately 8 mg of each sample was placed in a platinum crucible and heated from 25 to 800 °C at a heating rate of 10 °C/min under a nitrogen atmosphere with a flow rate of 50 mL/min. Mass loss was monitored as a function of temperature to identify thermal events related to moisture, dehydration, and/or oxidation of the sample constituents.

### N_2_ Adsorption

The specific surface area of the samples was determined using the Brunauer–Emmett–Teller (BET) gas adsorption technique, using a Micromeritics ASAP gas adsorption analyzer. These measurements were performed at the Federal Institute for Materials Research (BAM), Germany. The samples were previously degassed under vacuum at 150 °C for 2 h to remove contaminants and adsorbed moisture. The adsorption and desorption isotherms were obtained at 77 K, using nitrogen as the adsorbent gas. The surface area was calculated from the adsorption isotherm in the relative pressure range (P/P_0_) between 0.05 and 0.30, where the BET model is applicable. The analysis also provided information on pore volume and mean pore diameter, based on the BJH (Barrett–Joyner–Halenda) model, when appropriate.

### Electron Paramagnetic Resonance (EPR)

Measurements were performed on a Bruker Elexsys E580 spectrometer operating at 9.43909 GHz (X-band) using a modulation frequency and amplitude of 100 kHz and 1 G, respectively. The powdered samples were placed in quartz tubes and analyzed at 100 K.

### Solid-State NMR

^113^Cd and ^13^C NMR spectra were acquired in a Varian Unity INOVA spectrometer operating at 7.05 T (^1^H Larmor frequency of 300 MHz), equipped with a Varian 7 mm MAS probe operated at a spinning rate of 5 kHz. ^113^Cd NMR spectra were measured using single-pulse excitation with a 90° pulse of 6 μs, recycle delay of 400 s and approximately 200 scans. The recycling delay was chosen to ensure sufficient relaxation of the ^113^Cd magnetization between successive scans. ^13^C NMR spectra were measured using cross-polarization (CP) from ^1^H under magic angle spinning (MAS) conditions (^13^C­{^1^H} CPMAS). Typical parameters were: a 90° ^1^H excitation pulse of 4.1 μs, contact time of 1 ms, recycle delay of 5 s, up to 120 scans and continuous wave (cw) decoupling during acquisition. ^1^H solid-state NMR spectra were measured on a Bruker Avance Neo spectrometer operating at 14.1 T (600 MHz for ^1^H Larmor frequency) equipped with a Bruker HX 1.3 mm MAS probe operated at a MAS rate of 60 kHz. ^1^H MAS spectra were recorded using the DEPTH pulse sequence to suppress probe background signal,^37^ with 90° pulse duration of 1.4 μs and recycle delay of 6 s. ^113^Cd­{^1^H} Rotational Echo Double Resonance (REDOR)^38^ experiments were performed at a MAS rate of 5.0 kHz, using 90°- and 180°-pulses of 3.75 and 7.5 μs, respectively, for ^113^Cd and 180°-pulses of 7.5 μs for ^1^H. XY-4 phase cycling was used for the ^1^H recoupling π-pulses.^39^ ^113^Cd chemical shifts were referenced to 0.1 M Cd­(ClO_4_)_2_ in aqueous solution, using cadmium acetate dihydrate as secondary standard (^113^Cd-δ = −46 ppm).^40^ ^13^C and ^1^H chemical shifts are reported relative to tetramethylsilane (TMS) using α-glycine as secondary reference, ^13^C-δ­(C=O) = 176.5 ppm and ^1^H-δ­(NH_3_) = 8.5 ppm.^41,42^

^15^N­{^1^H} CPMAS NMR experiments were carried out on a Bruker AVANCE III spectrometer equipped with an Ascend 400 DNP magnet and a ^1^H/X/Y low-temperature probe operating at frequencies of 400.03 MHz for ^1^H and 40.54 MHz for ^15^N. Spectra were recorded at nominally 110 K at a spinning rate of 8 kHz. ^15^N­{^1^H} CPMAS spectra were recorded employing a 3.0 μs excitation pulse on ^1^H followed by a spin-lock pulse with ramped amplitude and a contact time of 4 ms. The recycle delay was set to 10 s and between 8000 and 12,000 scans were accumulated. The tppm15 decoupling sequence^43^ was applied to decouple protons during data acquisition. Spectra were referenced to liquid ammonia using glycine as secondary standard, ^15^N-δNH_3_ = 33.4 ppm.^44^

### NMR Cryoporometry

^1^H time-domain NMR (TD-NMR) measurements were carried out on a Bruker Minispec mq20 spectrometer operating at a ^1^H Larmor frequency of 20 MHz (B_0_ ≈ 0.5 T) and equipped with a variable-temperature (VT) probe head. The spectrometer dead time was 11.5 μs, and π/2 and π pulse lengths of 2.5 and 4.8 μs, respectively, were used. The longitudinal relaxation time (T_1_) was determined using the standard inversion–recovery pulse sequence, with recycling delays set to 3 T_1_ in all experiments. Although the Hahn spin–echo sequence is the standard approach in NMR cryoporometry, here we employ a modified scheme based on the Dipolar-Filtered Magic Sandwich Echo (DF-MSE) sequence.^35,45^ This adaptation provides the same information as the conventional spin–echo approach but offers advantages in signal normalization, measurement time, and reproducibility. A detailed description of the pulse sequence and its rationale is provided by Trebbi et al.^35^ and is presented in Figure S23 of the Supporting Information. DF-MSE experiments were performed with an echo time of 100 μs and a dipolar-filter duration of 3.4 μs for the reference measurement and 2 ms for the filtered measurement over the temperature range from −60 to 20 °C, in 1 °C increments, with a thermal equilibration time of 3 min at each temperature. Cryoporometry measurements were conducted using cyclohexane as the probe liquid in a cylindrical sample holder (4 mm × 5 mm) to ensure temperature homogeneity. Temperature control was achieved with a Bruker BVT system calibrated by placing a thermocouple at the sample position and correlating its reading with the temperature indicated by the NMR sensor. This calibration was validated by measuring the melting temperatures of cyclohexane, *p*-xylene, *t*-butanol, and menthol, resulting in a temperature precision of approximately ±0.5 °C. The DF-MSE experiment was used to selectively detect the liquid fraction of the probe fluid through the normalized signal intensity, denoted *I*_nDFMSE_. Monitoring *I*_nDFMSE_ as a function of temperature yields intensity-versus-temperature curves with upturns corresponding to the melting transitions of both confined and bulk fluids (Figure S23b, Supporting Information S5). The temperature difference between these transitions corresponds to the melting-point depression of the confined liquid, which is directly related to the mean pore diameter through the Gibbs–Thomson equation for cylindrical pores.^46^ In addition, the temperature derivative of the *I*_nDFMSE_(*T*) curve was associated with the relative pore-volume distribution through the same Gibbs–Thomson relationship.^46^ The derivative was calculated both directly from the experimental data and from a smoothed curve obtained by fitting a double-Boltzmann sigmoidal function. Further details on the use of DF-MSE in cryoporometry are provided in Supporting Information S5.

### DFT Calculations

All first-principles calculations were performed within the framework of periodic Kohn–Sham density functional theory (DFT)^47,48^ as implemented in the CASTEP 25.12 package.^49^ The exchange-correlation interactions were treated using the generalized gradient approximation (GGA) with the Perdew–Burke–Ernzerhof (PBE) functional.^50^ Core–valence interactions were described utilizing on-the-fly pseudopotentials.^51^ Following rigorous total-energy convergence testing, the plane-wave basis set kinetic energy cutoff was established at 1200 eV. Brillouin-zone integrations were performed utilizing Monkhorst–Pack *k*-point meshes,^50^ with a 4 × 5 × 5 grid selected as the optimal choice between computational cost and energy convergence for the subsequent calculations.

The initial structure coordinates for the orthorhombic dia-Cd­(Im)_2_ conventional cell, which is also composed of 32 nitrogen sites, were acquired from the Cambridge Structural Database (CCDC 205574).^34^ Since nuclear magnetic resonance (NMR) shielding tensors are sensitive to local geometric disposition and hydrogen positions are not always reliable, two structural relaxation protocols were evaluated: (i) fully internal coordinate relaxation with fixed cell parameter, and (ii) a free hydrogen and nitrogen (H/N) atomic relaxation and cell parameters with constrained other atoms positioning.

Magnetic shielding tensors were subsequently computed using the gauge-including projector augmented-wave (GIPAW) formalism.^51–55^ The constrained H/N-relaxed structural model yielded a highly resolved computational chemical shift pattern. Specifically, the calculations delineate one isolated nitrogen environment that is separated by 14.36 to 14.99 ppm from a near-degenerate cluster of three distinct nitrogen environments. This computationally derived relative separation and site multiplicity provides a theoretical foundation for the subsequent symmetry-based empirical assignment of the nitrogen sites. Other magnetic shielding values were calculated in the original cell depending only on Brillouin Zone symmetric points for inspection and can be found in the Supporting Information.

To evaluate the ^15^N chemical shifts from the GIPAW-DFT calculations, the isotropic magnetic shielding constants (σ_*DFT*_) were converted to the chemical shift scale (δ) referenced to liquid ammonia, using the standard relation δ_DFT_ = σ_ref_ – σ_DFT_ with σ_ref_ = 220.7 ppm. This reference constant was validated by calculating the ^15^N shielding of five reference compounds (nitromethane, ammonium chloride, α-glycine, imidazole and benzimidazole) at the same level of theory as the framework and regressing the calculated shielding against their independently measured solid-state ^15^N chemical shifts (see Supporting Information, Section S5). The resulting calibration (δ = 218.7–0.966·σ_DFT_, *R* ^2^ = 0.996, residual standard error 6.8 ppm, excluding nitromethane) is consistent with the σ_ref_ value used above and reproduces the calculated nitrogen shifts reported in Tables S8 and S9 to within 3 ppm of the experimental resonances.

## Results

Figure 1 shows the powder PXRD diffractogram for the samples in the series Cd­(Im)_2–*x*_(bIm)_*x*_, with *x* = 0, 0.02, 0.05, 0.22, and 0.40 (Im = imidazole, bIm = benzimidazole). For *x* = 0, the diffractogram in Figure 1 is in close agreement with data reported for the crystalline structure of the dia-Cd­(Im)_2_ coordination polymer (see Figure S21).^56^ The structure is characterized by a 2-fold interpenetrated diamond topology, forming a cadmium tetrahedral environment coordinated by four imidazole linkers.^56^ For samples with *x* > 0, the powder PXRD patterns in Figure 1 reveal a progressive loss of crystallinity with increasing bIm content, tending toward an amorphous material at higher bIm concentrations. Nevertheless, the coordination of bIm units to Cd^2+^ in the final compound is confirmed by the FTIR (Figure S2) and the solid-state NMR results described below. The same evolution from crystalline to increasingly amorphous materials with increasing benzimidazole content was previously reported by Xue et al.^33^ for samples prepared by mechanochemical synthesis, indicating that this behavior is intrinsic to the Cd­(Im)_2–*x*_(bIm)_*x*_ system rather than to the synthesis route. The progressive loss of crystallinity is likely associated with the incorporation of the bulkier benzimidazolate ligand into the densely packed dia-Cd­(Im)_2_ framework. The resulting steric constraints and increased chemical disorder hinder the formation of a periodic long-range structure while preserving the local coordination environment, thereby promoting the formation of a partially amorphous material.

**Figure 1:**
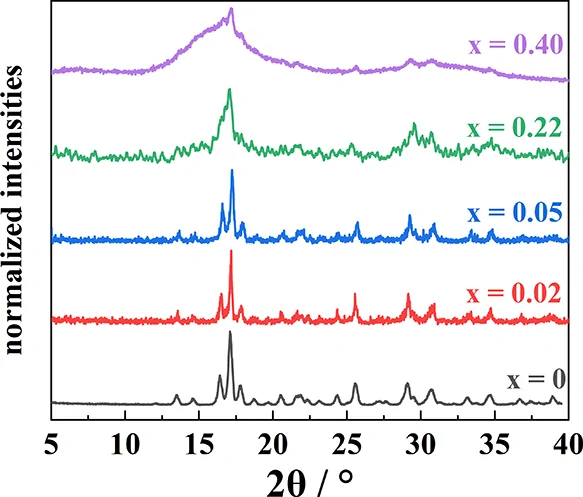
Powder PXRD patterns of Cd­(Im)_2–*x*_(bIm)_x_ samples, with *x* = 0, 0.02, 0.05, 0.22 and 0.40.

Nitrogen adsorption isotherms were measured to investigate the evolution of the textural porosity upon benzimidazole incorporation. Figure 2 shows the adsorption isotherms for samples with *x* = 0, 0.22, and 0.40 under N_2_ atmosphere. Different from traditional zinc-based ZIFs such as ZIF-4, which exhibits a BET surface area of 300 m^2^/g,^15^ dia-Cd­(Im)_2_ (*x* = 0) displays a significantly lower BET surface area of approximately 12.82 m^2^/g (Table 2). This low value reflects the dense 2-fold interpenetrated *dia*-framework, which possesses negligible intrinsic framework porosity. Accordingly, the adsorption isotherm is classified as type II,^57^ characteristic of nonporous or macroporous solids. The pore size distribution obtained from the BJH analysis therefore represents textural mesoporosity, arising from interparticle voids rather than the intrinsic cavities of the coordination framework.

**Figure 2:**
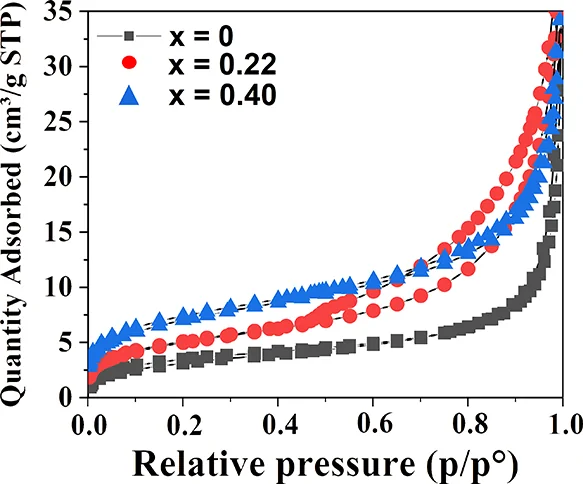
BET N_2_ adsorption–desorption analysis for Cd­(Im)_2–x_(bIm)_*x*_ samples with *x* = 0, 0.22, and 0.40.

**Table 2:** Parameters of Cd­(Im)_2–x_(bIm)_*x*_ Samples (*x* = 0, 0.02, 0.22 and 0.40) Obtained from BET and NMR Cryoporometry Analyses^a^

				**NMR cryoporometry**		
				**GT equation** (±1 nm)	**log-normal fitting** (±2 nm)	
**Cd(Im)**_**2–*x***_ **(bIm)**_**x**_	**BET surface area** (±0.1 m^**2**^ **/g)**	**pore volume** (±0.01 cm^**3**^ **/g)**	**average pore size** (±1 nm)	**average pore size**	**average pore size**	**full with at half maximum**
*x* = 0.40	26.0	0.06	9	15	10	4
*x* = 0.22	18.3	0.07	15			
*x* = 0.02				15	10	10
*x* = 0	12.8	0.05	17	22	16	14

a The table summarizes the BET surface area, pore volume, and average pore size, together with the mean pore diameters derived by direct application of the Gibbs–Thomson (GT) equation and by log-normal fitting of the NMR-derived pore-size distributions. The full width at half maximum (FWHM) values are also included to describe the relative broadness of the pore-size distributions

Upon increasing the benzimidazole content to *x* = 0.22, a hysteresis loop appears in the adsorption isotherm, indicative of capillary condensation within textural mesopores (Type IV isotherm).^57^ This behavior is accompanied by an increase in the BET surface area, a reduction in the average pore diameter, and an increase in the total pore volume (Table 2). For the composition with *x* = 0.40, the hysteresis loop disappears and the isotherm reverts to Type II behavior. At the same time, the BET surface area continues to increase, whereas the average pore diameter and total pore volume decrease (Table 2), indicating further changes in the material’s textural morphology rather than in its intrinsic framework porosity.

The textural pore structure of the Cd­(Im)_2–*x*_(bIm)_*x*_ series was further investigated by NMR cryoporometry using the DF-MSE pulse sequence with cyclohexane as the probe liquid and a filter time of *t*_e_ = 2 ms, following the procedure reported by Trebbi et al.^35^ Figure 3 displays the resulting pore size distributions, obtained from the derivative of the normalized DF-MSE intensity as a function of temperature and converted to pore diameter through the Gibbs–Thomson relation. Similar to gas adsorption, NMR cryoporometry probes mesoscopic confined spaces accessible to the probe liquid rather than the intrinsic cavities of the coordination framework. The resulting distributions therefore reflect the textural morphology of the materials and reveal clear differences in the characteristic confinement dimensions and distribution widths among the samples investigated.

**Figure 3:**
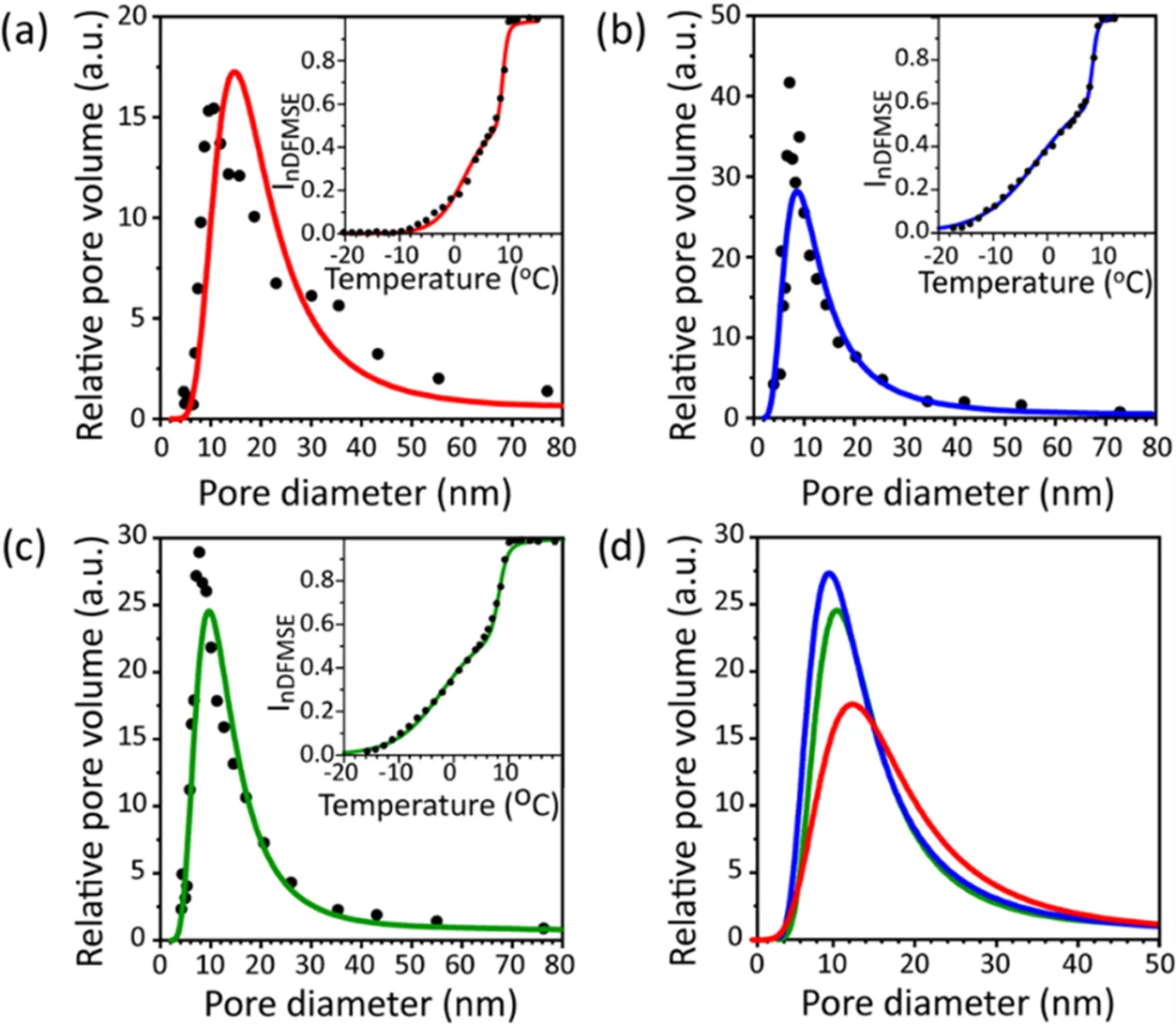
NMR cryoporometry results obtained using the Dipolar-Filtered Magic Sandwich Echo (DF-MSE) pulse sequence with cyclohexane as the probe molecule and filter time *t*_*f*_ = 2 ms). (a–c) Pore size distributions for Cd­(Im_)2–*x*_(bIm)_*x*_ samples with *x* = 0, 0.02, and 0.40, respectively, obtained from the derivative of the normalized DF-MSE intensity with respect to temperature. (d) Superimposed pore size distributions highlight the systematic shift and broadening of the pore population with increasing bIm content.

For the sample with *x* = 0.40, the cryoporometric curve exhibits a relatively narrow distribution centered at approximately 10 ± 2 nm (log-normal fit), consistent with a mean Gibbs–Thomson pore diameter of 15 ± 1 nm and a full width at half-maximum (fwhm) of about 4 nm. Along with the corresponding BET values (surface area = 25.7 m^2^/g; pore volume = 0.060 cm^3^/g) these data indicate a relatively uniform textural mesoporosity.

For *x* = 0.02, the Gibbs–Thomson analysis yields a mean pore diameter of 15 ± 1 nm, while the log-normal fitting points to a broader distribution centered at 10 ± 2 nm with an fwhm of approximately 10 nm, reflecting a greater heterogeneity of the textural mesoporosity.

The compound with *x* = 0 displays the largest and broadest pore population, with an average Gibbs–Thomson pore size of 22 ± 1 nm and a log-normal center at 16 ± 2 nm (fwhm ≈ 14 nm). Its BET surface area (12.8 m^2^/g) and pore volume (0.053 cm^3^/g) are both lower than those of the substituted samples, consistent with a coarser textural morphology characterized by larger interparticle voids.

Although the *x* = 0.22 composition was characterized by BET (18.3 m^2^/g; 0.067 cm^3^/g), no NMR cryoporometry measurement was performed for this sample. The BET results nevertheless suggest that it represents an intermediate regime between *x* = 0.02 and 0.40.

Overall, the cryoporometry results indicate that benzimidazole substitution leads to a progressive reduction in the characteristic dimensions of the confined spaces together with a narrowing of their distribution. This trend is qualitatively consistent with the nitrogen adsorption measurements, which also indicate systematic changes in the textural mesoporosity with increasing benzimidazole content. However, because NMR cryoporometry and gas adsorption probe different aspects of the mesoscale structure, quantitative agreement between the characteristic dimensions obtained by the two methods is not expected. Rather than reflecting changes in the intrinsic porosity of the coordination framework, both techniques primarily probe the evolution of the material’s textural morphology during synthesis and vitrification.

Figure 4 shows the ^13^C­{^1^H} CPMAS spectra for all samples. The ^13^C spectrum for *x* = 0 shows peaks in two characteristic regions, around 127 and 147 ppm, which can be attributed to C1 and C2 Im groups (see scheme in Figure 4), respectively.^58^ For *x* > 0, the ^13^C spectra show additional peaks at 150, 142, 122, and 115 ppm, corresponding to carbon species of the bIm molecules (see attributions in Figure 4). These resonance peaks are typical of bIm units incorporated in the polymeric structure of ZIFs.^13^ The ^13^C­{^1^H} CPMAS spectra show no evidence of unreacted Im and bIm precursors, as evidenced by comparison with the ^13^C spectra of the precursors (Figure S5). This result confirms that the bIm^–^ molecule is not simply adsorbed within the structure but rather forms a chemical bond with the cadmium metal center. While PXRD results show a loss of long-range order, ^13^C NMR shows that the short-range order is preserved, to some extent, for the organic linkers. Only moderate line broadening due to chemical shift distributions is observed for increasing bIm content.

**Figure 4:**
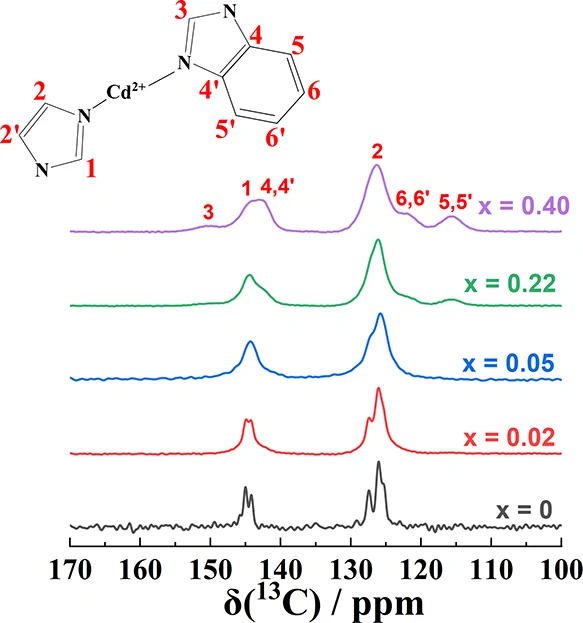
^13^C­{^1^H} CPMAS NMR spectra of Cd­(Im)_2–*x*_(bIm)_*x*_ samples. The inset shows the schemes of the ligand molecules, Im and bIm. Numbers on the spectra are attributions to the carbons in the scheme.

Figure 5 shows ^113^Cd NMR spectra for the investigated compounds. For all samples, the ^113^Cd spectra show a single line centered around 437 ppm. This chemical shift is attributed to Cd tetracoordinated with nitrogen atoms.^58^ Simulation of the spinning sideband pattern for the compound with *x* = 0, dia-Cd­(Im)_2_ (see Figure S6 and Table S1), yields chemical shift anisotropy and asymmetry parameters of 126 ppm and 0.7, respectively, in agreement with previously reported values.^56,58^ For increasing *x* values, the spectral lines of ^113^Cd exhibit a broadening effect, reflecting the short-range disorder in the Cd coordination environment, in good agreement with the amorphization detected by PXRD analysis.

**Figure 5:**
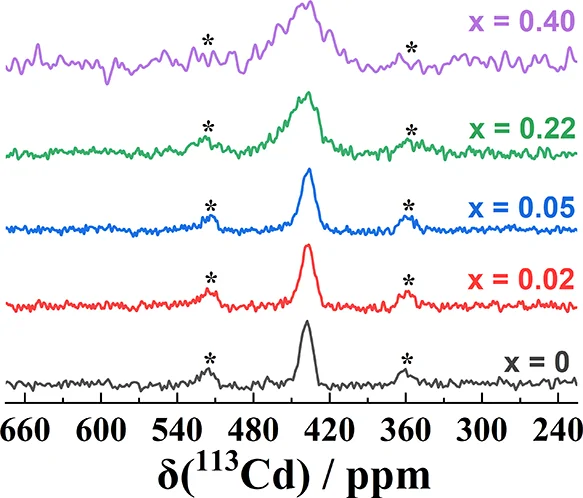
^113^Cd NMR spectra of Cd­(Im)_2–*x*_(bIm)_*x*_ samples. Asterisks indicate spinning sidebands.

The glass-forming ability of Cd­(Im)_2–*x*_(bIm)_*x*_ compounds was investigated via differential scanning calorimetry (DSC). As depicted in Figure 6, each specimen was subjected to two thermal cycles: initial heating to induce melting, followed by rapid cooling to facilitate glass formation; subsequently, a second heating cycle was performed to identify the glass transition temperature. The presence of a glass transition during the second scan is an indication of the successful formation of the glass phase.

**Figure 6:**
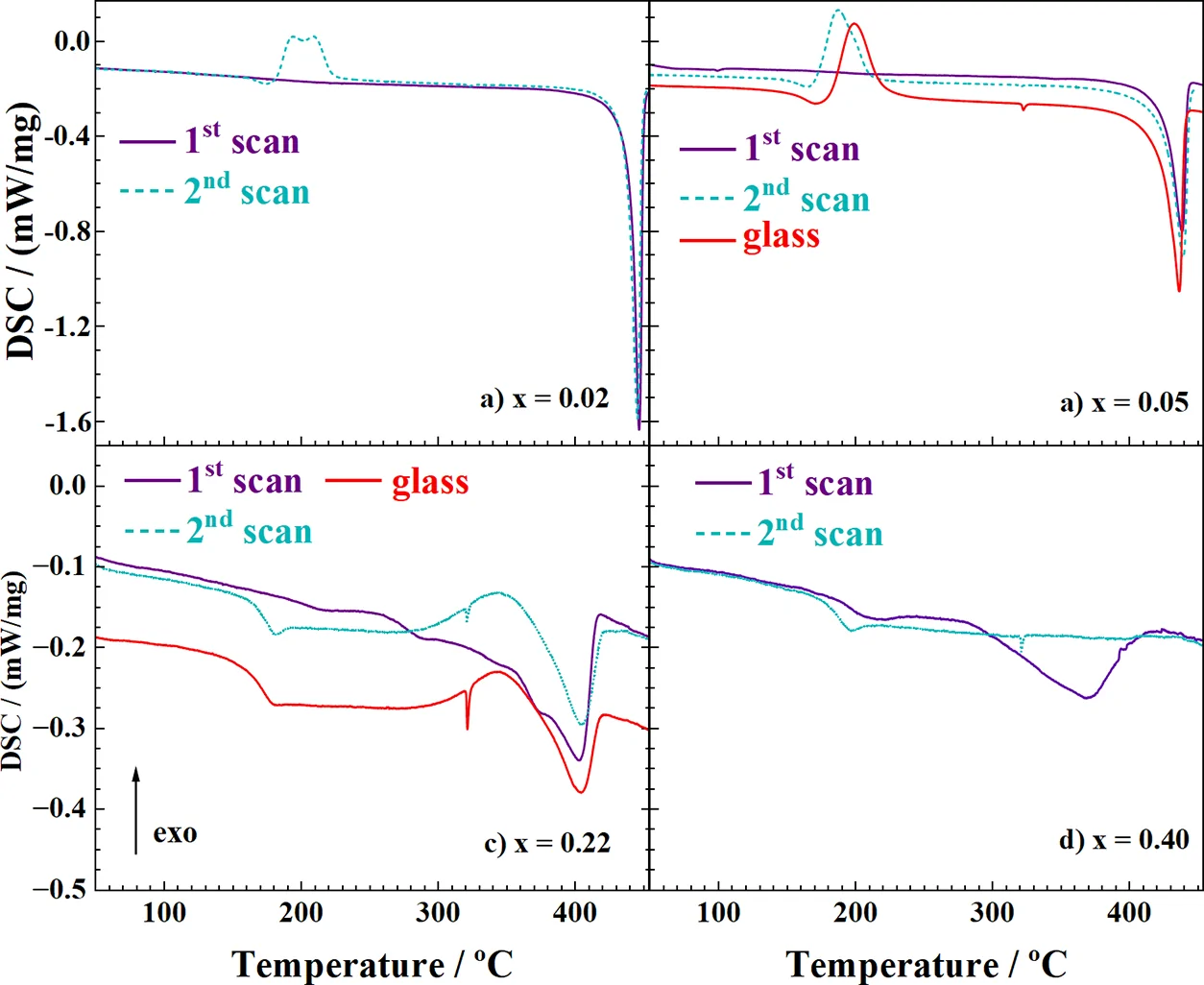
DSC analysis with two thermal cycles for materials (a) *x* = 0.02, (b) *x* = 0.05, (c) *x* = 0.22 and (d) *x* = 0.40. For *x* = 0.22 and 0.40, the DSC curve for glasses prepared in a furnace under a controlled N_2_ atmosphere are also shown in red.

For *x* = 0, the melting endothermic peak was observed at 452 °C in the first upscan, consistent with previous reports for this material (Figure S18).^33^ The corresponding melting enthalpy was −89.85 J·g^–1^ (Table S2). In the second heating cycle, no glass transition event was observed, whereas metallic cadmium was identified from its melting event at 321 °C. The Cd^0^ content was estimated to be approximately 0.17 wt % based on the ratio between the enthalpy of the cadmium melting event and the total melting enthalpy of the material. Powder PXRD results further show that crystallinity is maintained after a melting/cooling cycle under an N_2_ atmosphere, although a decrease in the number of counts is observed, which may be associated with partial decomposition (Figure S20). Therefore, in contrast to the results reported by Xue et al.,^33^ the compound with x = 0 synthesized via our chemical route does not form a complete glass upon melt-quenching, even at cooling rates of approximately 40 °C/min and retains some degree of crystallinity after the heat treatment. Louis Frentzel-Beyme and collaborators have discussed that ZIFs composed exclusively of Im-, such as dia-Cd­(Im)_2_, typically exhibit limited glass-forming ability and do not form a stable liquid at low temperatures.^36^ The thermogravimetric analysis (TGA, Figure S17) indicates that the thermal decomposition of dia-Cd­(Im)_2_ occurs very close to its melting temperature, with a weight loss of approximately 2.5% at 449 °C, corresponding to partial decomposition. Around 500 °C, a second decomposition stage occurs, resulting in a 30% weight loss, followed by a third and almost complete mass loss at approximately 660 °C.

The occurrence of metallic cadmium upon melting indicates that dia-Cd­(Im)_2_ undergoes partial thermal decomposition during high-temperature processing. A similar phenomenon has recently been reported for Cd­(im)_2_ glasses, where the formation of metallic Cd was attributed to the partial decomposition of imidazolate ligands, whose decomposition products promote the reduction of Cd^2+^ during melting.^33^ The reduction of metal ions to the metallic state during thermal decomposition has also been reported for Co- and Cu-based MOFs.^59,60^ In the present work, however, the low intensity of the metallic Cd melting peak (0.17 wt %) together with the absence of detectable Cd reflections in the X-ray diffraction patterns indicate that metallic Cd constitutes only a minor fraction of the material.

For the *x* = 0.02 sample, no major change in the thermal response was observed during the first DSC heating scan, except for a decrease in the melting temperature to 446 °C (Figure 6a). The corresponding melting enthalpy was −73.17 J g^–1^ (Table S2). During the second heating scan, an exothermic event was observed at approximately 193 °C, with an associated enthalpy of 32.5 J g^–1^, and a weak glass transition event at approximately 166 °C. A weak endothermic event attributable to metallic Cd was also detected during the second heating scan, corresponding to an estimated Cd fraction of approximately 0.15% of the molten material. The relatively small magnitude of this signal, together with the absence of detectable metallic-Cd reflections by PXRD, indicates that metallic Cd is present only as a minor component.

For *x* = 0.05, the melting temperature further decreased to approximately 440 °C in the first upscan (Figure 6b), with a corresponding melting enthalpy of −65.71 J·g^–1^ (Table S2). During the second heating scan, a clear glass transition was observed at approximately 144 °C, confirming the formation of a glass phase. The glass-forming ability, determined from the reduced glass-transition temperature, *T*_g_/*T*_m_ (in Kelvin units), was approximately 0.58. Metallic cadmium was again detected in the second heating scan, corresponding to approximately 0.11% of the molten material’s composition. Xue et al. reported a *T*_g_ of 179 °C for the *x* = 0.05 sample.^33^ The *T*_g_ observed for this cadmium-based glass is considerably lower than those reported for traditional zinc-based ZIF glasses, with *T*_g_ values of approximately 294 °C for ZIF-4 and 322 °C for ZIF-62.^13,61^ PXRD further confirms the absence of long-range structural order in the melt-quenched material (Figure S22). Upon further heating, crystallization occurs at approximately 187 °C, with an associated enthalpy of 42.3 J·g^–1^. This glass-to-crystal transformation is consistent with the crystallization behavior previously reported by Xue et al. for the same compositions.^33^

The initial DSC profile of the *x* = 0.22 sample exhibited a complex thermal response, with several endothermic and exothermic events. Based on successive heating–cooling experiments, these features were found to progressively disappear upon repeated thermal cycling, with treatment at 300 °C being sufficient to complete the irreversible processes observed during the initial heating. Accordingly, all samples were thermally treated at 300 °C prior to the DSC measurements discussed below. After this treatment, the DSC profiles became considerably simpler, facilitating the identification of the melting event during the first heating scan and the glass-transition and crystallization events during the subsequent heating scan. The origin of the individual irreversible thermal events cannot be unambiguously established from the present measurements. They may involve structural relaxation and/or amorphous–amorphous transformations, as well as the removal of residual or trapped solvent. Because a detailed assignment of these processes is beyond the scope of the present work, we focus here on the reproducible melting, glass-transition, and crystallization events observed after thermal treatment. In the first heating cycle, the treated sample exhibited a reduced melting temperature of 404 °C. The experimental *x*-value observed by liquid-state ^1^H NMR is 0.22. Xue et al. reported the value of 427 °C for the composition *x* = 0.25.^33^ The corresponding melting enthalpy was −37.32 J·g^–1^ (Table S2). During the second heating scan, a glass transition was observed at approximately 170 °C, followed by crystallization centered at 344 °C, with an associated enthalpy of 19.1 J·g^–1^. The glass-forming ability of this material was approximately 0.65, higher than that determined for the *x* = 0.05 sample. The PXRD pattern further confirmed the absence of long-range structural order, consistent with the formation of a glassy phase (Figure S22). Diffraction peaks attributable to CdO were also observed in the *x* = 0.22 glass, providing evidence for partial decomposition during thermal treatment. A weak endothermic event attributable to metallic Cd was detected in the second heating scan, corresponding to an estimated fraction of approximately 0.5%.

A similar complex thermal response was observed for the *x* = 0.40 sample in the initial heating scan, which became more clearly resolved after thermal treatment at 300 °C (Figure 6d). No significant changes in the melting or glass-transition temperatures were observed after this treatment. During the first heating scan, a broad endothermic event centered at 368 °C was observed, corresponding to the lowest melting temperature in the investigated series. The associated enthalpy was 33.37 J g^–1^. During the second heating scan, a glass transition was observed at 181 °C, with no subsequent crystallization event detected within the investigated temperature range. The glass-forming ability was approximately 0.68. A weak endothermic event attributable to metallic Cd corresponded to an estimated fraction of approximately 0.35%.

Across the series, increasing benzimidazolate content is accompanied by a decrease in the melting temperature and melting enthalpy. A similar decrease in melting temperature with increasing bIm content was reported by Xue et al. for these Cd-based frameworks.^33^ The authors related this behavior to the increased structural disorder associated with benzimidazole incorporation. Although the present data does not allow the microscopic origin of this trend to be established unambiguously, the incorporation of the bulkier bIm linker is expected to increase structural heterogeneity and frustrate efficient packing of the framework. This interpretation is consistent with the progressive loss of crystallinity observed by PXRD and with the suppression of recrystallization at higher bIm contents. In contrast, ZIF-62 exhibits the opposite trend, with the melting temperature increasing as the benzimidazole content increases.^13^ The glass transition temperature also increases with increasing benzimidazole content, in agreement with the trend reported by Xue et al.^33^ and previously observed for the glass-forming ZIF-62.^13^ In addition, the crystallization temperature shifts toward higher values as the benzimidazole content increases, while the enthalpy associated with crystallization decreases. The increase in benzimidazole content enhances the glass-forming ability of the material, as evidenced by the increase in the reduced glass-transition temperature from 0.58 at *x* = 0.05 to 0.65 at *x* = 0.22 and 0.68 at *x* = 0.40. These changes are consistent with increasing resistance to structural rearrangement and crystallization as the fraction of the bulkier bIm linker increases. Thus, benzimidazolate incorporation modifies the balance between melting, glass transition, and crystallization, favoring the retention of the disordered state upon cooling.

Altogether, we observed that sample *x* = 0, so-called dia-Cd­(Im)_2_, upon melting and cooling maintains partial crystalline characteristics (Figure S21). To control the rapid recrystallization of the unstable liquid and promote the glass formation, the presence of a larger coligand is usually required to create steric hindrance.^36^ Beyme et al. hypothesize that a high number of crystalline ZIFs can exhibit a stable liquid phase when composed of Im^–^ and a larger ligand. Since dia-Cd­(Im)_2_ consists only of small Im^–^ ligands, its structure melts due to its high steric accessibility, but forms a metastable liquid phase that easily recrystallizes. For this reason, as the benzimidazole content increases in the Cd­(Im)_2–*x*_(bIm)_*x*_ system a clear glass behavior is observed. Benzimidazole is a large imidazolate-type linker, which can form large tetrahedral units, thereby enhancing steric hindrance.^36^ Higher steric hindrance, entropy and structure asymmetry can increase glass formation ability due to the greater kinetic impediment that the molecules will encounter during cooling, favoring the glass formation. Although metallic Cd is formed as a minor decomposition product during high-temperature treatment, its role in the crystallization behavior appears to be secondary. Xue et al.^33^ likewise reported that increasing benzimidazole content progressively suppresses recrystallization, even though only trace amounts of metallic Cd were detected in their samples. This comparison suggests that the reduced recrystallization tendency is primarily governed by the structural modifications introduced by benzimidazole substitution rather than by the presence of metallic Cd.

Solid-state NMR experiments were performed to compare the local structure of Cd­(Im)_2–*x*_(bIm)_*x*_ glasses with their crystalline counterparts. Figure 7 shows the ^13^C­{^1^H} CPMAS spectra for the as-synthesized *x* = 0.05 and 0.22 samples and their corresponding glassy phase. In both cases, only minor line broadening is observed upon glass formation, indicating that the local chemical environments of the imidazole and benzimidazole linkers are preserved. This result is consistent with previous studies of MOF glasses, where vitrification typically retains the short-range order of the organic framework.

**Figure 7:**
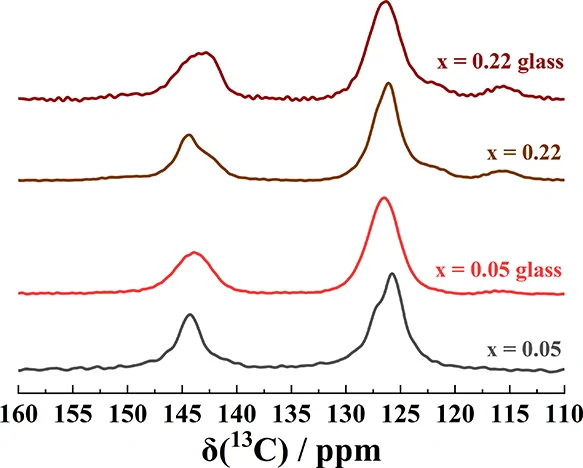
Comparison of ^13^C­{^1^H} CPMAS NMR spectra of Cd­(Im)_2–*x*_(bIm)_*x*_ (*x* = 0.05 and 0.22 samples) as synthesized and as a glass.

In contrast, the ^113^Cd MAS NMR spectra (Figure 8) are more sensitive to the structural changes induced by vitrification. While the as-synthesized materials already exhibit some degree of line broadening due to the compositional disorder introduced by bIm incorporation (Figure 5), the corresponding glasses display substantially broader and slightly asymmetric resonances (see Figures S9 and S11). Notably, both glass compositions exhibit nearly identical ^113^Cd line shapes despite their different linker contents, suggesting that vitrification generates similar distributions of local cadmium environments. The observed broadening and asymmetry are consistent with a distribution of Cd coordination geometries arising from variations in bond angles and distances around the metal centers. Other types of defects, such as broken coordination bonds or dangling linkers, may also be present. However, if such defects exist, their concentration is expected to be below the detection limit of the solid-state NMR experiments and, therefore, they are not resolved in the spectra. This behavior is consistent with previous observations for Cd-based MOF glasses^33^ and with ultrahigh-field ^67^Zn NMR studies on ZIF-62 glass,^28^ which showed that amorphization in ZIFs is accompanied by the development of short-range disorder at the metal nodes while maintaining the underlying metal–ligand connectivity.

**Figure 8:**
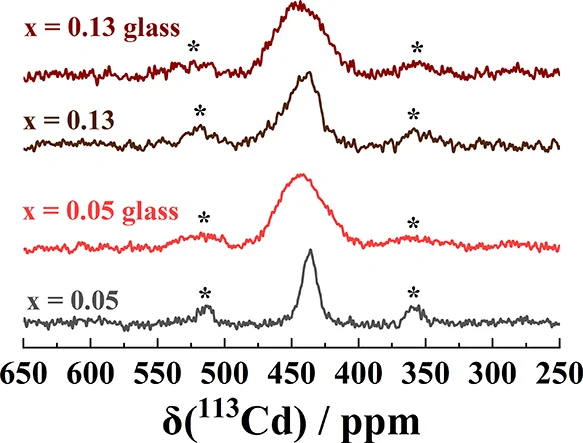
Comparison of ^113^Cd MAS NMR spectra of Cd­(Im)_2–*x*_(bIm)_*x*_ (*x* = 0.05 and 0.22 samples) as synthesized and as a glass. Asterisks indicate spinning sidebands.

To evaluate the local coordination geometry of the nitrogen atoms, low-temperature (110 K) ^15^N­{^1^H} CPMAS NMR spectra were acquired for the as-synthesized and glassy states of samples with *x* = 0.05 and 0.22 (Figure 9). The crystalline materials display three well-defined resonances at 216, 207, and 202 ppm. To unambiguously assign these sites, DFT calculations were performed for the fully crystalline dia-Cd­(Im)_2_ structure, using a geometry optimization in which hydrogen and nitrogen positions were relaxed with a variable unit cell.^34^ The calculated values for dia-Cd­(Im)_2_ are 206, 207, 209, and 221 ppm (dashed lines in Figure 9, Table S8); this result is corroborated by two additional, independently converged geometry-optimization protocols reported in the Supporting Information (Tables S8 and S9), which give the same one-isolated/three-clustered nitrogen topology. Three of these sites are clustered within a narrow chemical-shift range, whereas the fourth is significantly separated, reflecting the asymmetric tetrahedral coordination environments around the Cd centers. The calculated shift pattern closely reproduces the experimental observation of one isolated resonance and a second group of overlapping resonances. The signals at 216 and 207 ppm are assigned, based on the periodic DFT calculation, to nitrogen atoms belonging to imidazolate linkers coordinated with Cd. The resonance signal at 202 ppm has no counterpart among the four calculated dia-Cd­(Im)_2_ nitrogen environments in any of the three geometry-optimization protocols tested (Supporting Information, Tables S7–S9) and is assigned by exclusion to nitrogen atoms in benzimidazolate linkers. This assignment is supported by a chemical-shift-trend argument: GIPAW calculations on the isolated benzimidazole and imidazole reference crystals show a consistent 10–15 ppm increase in shielding for both the pyridine-type and pyrrole-type nitrogen environments (Supporting Information, Section S5), placing a benzimidazolate-type nitrogen 10–15 ppm below its imidazolate counterpart on the chemical-shift scale, consistent with the observed 202 ppm resonance (although not based on a direct periodic calculation), and by the analogous differentiation between imidazolate and benzimidazolate nitrogen sites previously reported for ZIF-62.^62^ Because no periodic model containing benzimidazolate or the amorphous phase was calculated in this work, this specific assignment should be regarded as supported by reference-compound analogy rather than by direct periodic DFT calculation.

**Figure 9:**
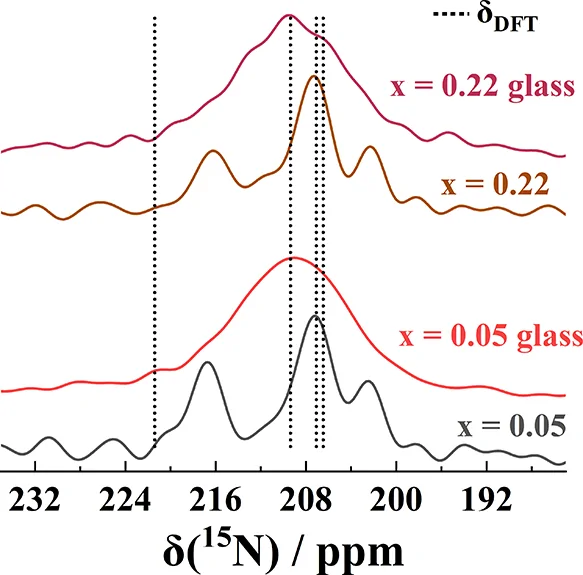
^15^N­{^1^H} CPMAS NMR spectra of as-synthesized and glass Cd­(Im)_2–*x*_(bIm)_*x*_ samples for *x* = 0.05 and 0.22 measured at 110 K. Dashed lines represent chemical shift values obtained from DFT calculations (Table S8 in Supporting Information) for the crystalline Cd­(Im)_2_ compound.

In the glassy state, the onset of local structural disorder is clearly indicated by the broadening of these resonance lines. Because the nitrogen atoms are directly coordinated to the metal center, this broadening mirrors the distribution of distorted geometries around the cadmium nodes observed in the ^113^Cd NMR spectra (Figure 8). Remarkably, despite this broadening, no new chemical sites or decomposition products are observed in the spectra. This indicates that, although the local geometry around cadmium is distorted during the vitrification process, the Cd–N connectivity is preserved.

The local environment of the Cd^2+^ centers was further probed via ^113^Cd­{^1^H} REDOR experiments (see Figure 10). From these measurements, it is possible to extract information about the second moment (*M*_*2*_) of the ^113^Cd–^1^H heteronuclear dipolar interaction, which directly correlates with both the average Cd–H distance and the number of neighboring protons surrounding the cadmium sites.^63^ In practice, for complex systems the structural information is primarily encoded in the initial dephasing regime. However, because of the low MAS frequency available (5 kHz) and the relatively strong REDOR dephasing observed for these samples, a reliable quantitative determination of *M*_2_ or individual Cd–H distances was not possible.

**Figure 10:**
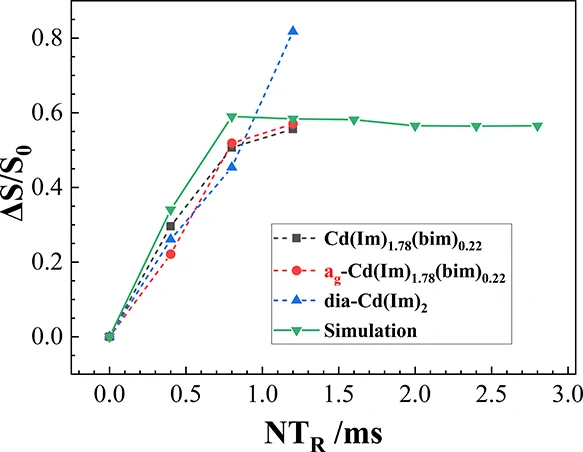
^113^Cd­{^1^H} REDOR dephasing curves obtained for crystalline and glassy Cd­(Im)_1.78_(bIm)_0.22_ and for dia-Cd­(Im)_2_ crystal. The green curve represents the REDOR dephasing simulated using SIMPSON for a simplified five-spin model based on the average ^113^Cd–^1^H and ^1^H–^1^H dipolar couplings obtained from the crystalline dia-Cd­(Im)_2_ structure. Dashed lines are drawn as guide for the eyes.

To further assess the observed dephasing behavior, numerical REDOR simulations were performed using SIMPSON.^64^ A simplified five-spin model consisting of one ^113^Cd spin surrounded by four ^1^H spins was considered, with effective ^113^Cd–^1^H and ^1^H–^1^H dipolar couplings derived from the average couplings calculated for the crystalline dia-Cd­(Im)_2_ structure. The values used were M_2_(^113^Cd–^1^H) = 4.83 × 10^7^ rad^2^ s^–2^ and M_2_(^1^H–^1^H) = 3.64 × 10^9^ rad^2^ s^–2^. The simulations were performed using the same experimental parameters as the REDOR measurements. The SIMPSON input file is provided as Supporting Information. As shown in Figure 10, the initial dephasing of the simulated curve is in close agreement with the experimental dephasing. For the crystalline dia-Cd­(Im)_2_ sample, the simulated curve lies slightly above the experimental data, indicating a small underestimation of the dipolar dephasing in the experiment relative to that expected from the crystallographic model. Such an underestimation of the experimentally observed second moment is a well-recognized feature of REDOR measurements and can arise from experimental imperfections, including nonideal radio frequency pulses. Importantly, the crystalline dia-Cd­(Im)_2_ sample provides a reference structure with known average Cd–H and H–H dipolar coupling constants, allowing the comparison between the experimental and simulated curves to serve as an experimental benchmark for the REDOR measurements. The good agreement between the two, despite the slight systematic difference, indicates that the simplified spin model and the experimental conditions provide a reasonable description of the observed dephasing.

The close correspondence of the experimental REDOR curves for dia-Cd­(Im)_2_, crystalline *x* = 0.22, and *x* = 0.22 glass therefore provides semiquantitative support for the preservation of the average ^113^Cd–^1^H dipolar interaction upon vitrification. This conclusion is fully consistent with the pair distribution function (PDF) analysis reported by Xue et al., which likewise showed that the first coordination shell around Cd is largely preserved upon vitrification.^33^ The present multinuclear solid-state NMR results complement this structural picture by demonstrating that, although the predominant local coordination environment is retained, vitrification is accompanied by increased local geometric disorder at the Cd sites. Accordingly, the pronounced line broadening observed in the ^113^Cd NMR spectra is primarily attributed to a distribution of local Cd geometries, particularly variations in Cd–N–Cd bond angles, rather than to extensive disruption of the Cd–N coordination network. While the presence of minor coordination defects cannot be excluded, the combined NMR and REDOR results indicate that such defects do not dominate the average local structure.

Defects, such as color centers, may also form during the vitrification process.^65^ The as-synthesized materials appear as white powders, whereas the glasses obtained after melt-quenching exhibit a dark brown color. To investigate potential paramagnetic defects induced by vitrification, EPR experiments were conducted. Figure 11a compares the EPR spectra of glassy Cd­(Im)_1.78_(bIm)_0.22_ and ZIF-62, normalized by the number of moles. A marked difference in intensity is observed, consistent with their visual appearance: Cd­(Im)_1.78_(bIm)_0.22_ forms a dark brown glass, whereas ZIF-62 is typically light brown. Despite this difference in intensity, both samples exhibit nearly identical EPR line shapes and *g*-values. This similarity, despite the different metallic species present in the two frameworks, suggests that the paramagnetic centers originate from the organic linker sublattice rather than from the metal nodes.

**Figure 11:**
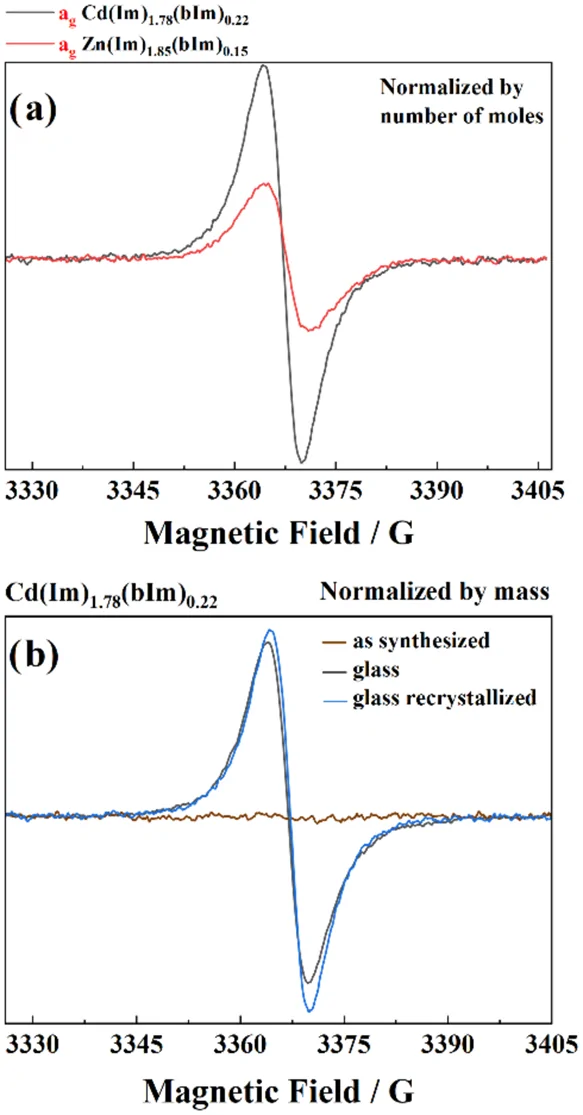
(a) Comparison of the EPR spectra of glassy Cd­(Im)_1.78_(bIm)_0.22_ and ZIF-62, normalized by the number of moles. (b) EPR spectra of Cd­(Im)_1.78_(bIm)_0.22_ in the as-synthesized, glassy, and recrystallized states (annealed at the DSC crystallization peak, 320 °C), normalized by sample mass.

To further examine the formation of these defects, Figure 11b shows the EPR spectra of Cd­(Im)_1.78_(bIm)_0.22_ in its as-synthesized, glassy, and recrystallized states (annealed at the DSC crystallization peak of 320 °C). A broad resonance centered at *g* = 2.0023 emerges only after thermal processing, demonstrating the generation of paramagnetic centers during vitrification. Signals in this region are commonly associated with organic radical species or delocalized π-electron systems exhibiting negligible spin–orbit and hyperfine interactions.^66,67^ Similar defect states have been reported in thermally treated ZIF-8 and attributed to modifications of the organic framework.^68^ The absence of resolved hyperfine structure in the present case suggests a high concentration of interacting defects and/or a distribution of local environments. Following recrystallization, the signal intensity increases slightly, indicating that defect formation is largely irreversible and accumulates during thermal cycling, consistent with progressive structural modification of the framework.

The nearly identical REDOR dephasing observed for the crystalline and glassy samples indicates that the average distribution of protons around Cd remains essentially unchanged after vitrification. Because REDOR probes ensemble-averaged heteronuclear dipolar interactions, these measurements indicate that the predominant local coordination environment around Cd is largely retained during glass formation. Nevertheless, the technique is inherently insensitive to a minor population of structural defects and therefore cannot exclude the presence of low concentrations of coordination defects. In this context, the EPR results provide complementary evidence that thermally induced paramagnetic defect centers are generated during high-temperature processing. Although the present data do not allow the microscopic nature of these defects to be established, their *g*-values are consistent with organic or carbon-centered paramagnetic species. Contributions from radicals associated with thermally induced ligand decomposition or carbonization therefore cannot be excluded.^69^ Similar defect species, including dangling-linker defects, have recently been proposed for related Zn-based MOF glasses.^70^ However, further experimental and computational studies will be required to establish whether analogous defect structures are present in the Cd-based system investigated here.

The formation of paramagnetic defect centers during vitrification may also have broader implications for the electronic and optical properties of Cd-ZIF glasses. Recently, Li et al.^71^ reported broadband white-light emission in ZIF-62 glasses and attributed this behavior to defect-induced modifications of the electronic structure. Although photoluminescence was not investigated in the present work, the direct observation of thermally generated paramagnetic defects by EPR provides independent evidence that defect formation accompanies vitrification. Whether the EPR-active centers identified here correspond directly to the emissive defect states proposed by Li et al. remains an open question. Further investigation of the emissive properties of Cd-ZIF glasses will be necessary to establish possible structure–property relationships.

To probe the molecular dynamics associated with vitrification, static ^1^H Hahn-echo NMR experiments were performed as a function of temperature. Representative spectra are shown in Figure S13 (Supporting Information). The spectra consist of a broad component superimposed with a narrower resonance. The broad signal is attributed to rigid framework regions dominated by strong ^1^H–^1^H dipolar couplings, whereas the narrow component originates from more mobile proton environments. Because the broad component exhibits only minor temperature-dependent changes and partially overlaps with the narrow resonance, the analysis presented in Figure 12 focuses exclusively on the full width at half-maximum (fwhm) of the narrow component.

**Figure 12:**
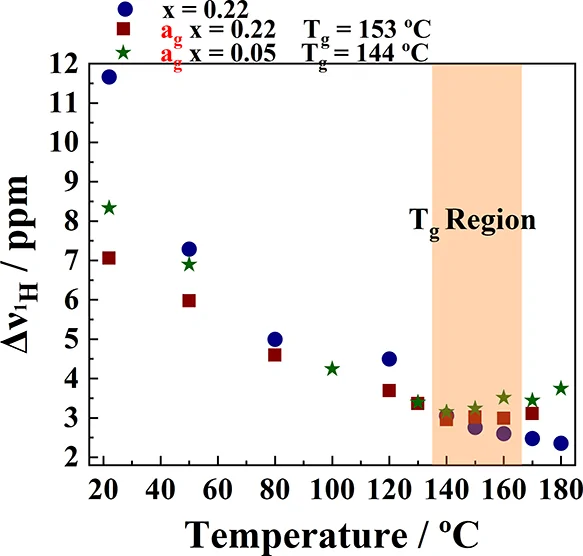
fwhm of the ^1^H ssNMR resonance peaks as a function of temperature for Cd­(Im)_1.78_(bIm)_0.22_ in the as-synthesized and glassy states, and for glassy Cd­(Im)_1.95_(bIm)_0.05_. a_g_ = glass state. The shaded area indicates the region where glass transition takes place. More details are provided in the Supporting Information material.

For all samples, the line width of the narrow resonance decreases with increasing temperature, reflecting the progressive motional averaging of dipolar interactions.^72,73^ However, marked differences are observed between the glassy and crystalline materials. For the glasses, the fwhm reaches a minimum in the vicinity of *T*_g_ (140–160 °C), indicating a significant increase in molecular mobility and the activation of dynamic processes associated with the glass transition.^74^ This behavior contrasts with that of the crystalline precursor, which exhibits a continuous line width narrowing throughout the investigated temperature range, consistent with thermally activated local motions within an ordered framework.

Above *T*_g_, the glasses display a renewed line width broadening. Notably, this temperature region coincides with the crystallization events observed by DSC, suggesting that the formation of increasingly rigid crystalline domains progressively restricts molecular motion and restores dipolar broadening. The distinct temperature dependence observed for the glasses therefore reflects the competition between mobility enhancement near *T*_g_ and structural reorganization during crystallization.

Each temperature step was maintained for approximately 1.5 h to ensure thermal equilibration and structural relaxation. Although preliminary, these results demonstrate the potential of static ^1^H NMR as a sensitive probe of dynamic processes occurring during glass transition and crystallization in MOF-derived glasses. While most previous studies have focused primarily on the structural characterization of the initial and final states, the present measurements provide direct spectroscopic insight into the evolution of molecular dynamics across the vitrification pathway.

## Conclusions

In this work, we investigated the structural evolution of as-synthesized and glassy Cd­(Im)_2–*x*_(bIm)_*x*_ series and established the role of benzimidazole incorporation in enabling glass formation in this family of cadmium-based ZIFs. While the parent dia-Cd­(Im)_2_ readily recrystallizes upon cooling from the melt, increasing bIm content introduces steric constraints and structural asymmetry that suppress crystallization and stabilize the glassy state. This behavior is associated with the incorporation of the bulkier bIm ligand into the densely packed dia framework, which increases structural disorder and hinders the development of long-range periodic order.

A combination of solid-state NMR, EPR, thermal analysis, and complementary characterization techniques reveals that vitrification preserves the fundamental metal–ligand connectivity while introducing substantial local disorder. ^13^C, ^15^N, and ^1^H NMR spectra demonstrate that the short-range order of the organic linker network remains largely intact after melt-quenching. In contrast, the pronounced broadening observed in the ^113^Cd spectra evidences a distribution of local cadmium environments in the glass. Importantly, ^113^Cd­{^1^H} REDOR measurements, supported by comparison with crystalline dia-Cd­(Im)_2_ and by SIMPSON simulations based on the crystalline structure, further indicate that the average ^113^Cd–^1^H dipolar interaction is largely preserved upon vitrification. Together, these results support the retention of the predominant local Cd coordination environment while indicating increased geometric disorder at the Cd sites.

Thermal processing also produces minor amounts of decomposition products, including metallic Cd and CdO. The EPR measurements reveal thermally induced paramagnetic centers, but their microscopic origin cannot be established unambiguously. Contributions from coordination-related defects, ligand-centered or carbon-centered radical species, and processes associated with thermal decomposition cannot be excluded. Similarly, the present NMR measurements cannot rule out a minor population of broken coordination bonds or dangling-linker-type defects. Nevertheless, the combined NMR and REDOR results indicate that such minor species do not dominate the average local structure observed in the glass.

The present work complements the structural framework established by Xue et al.^33^ by providing a comprehensive multinuclear solid-state NMR perspective on the local structural disorder of Cd-ZIF glasses. Whereas previous PDF analysis demonstrated that the first coordination shell around Cd is largely preserved upon vitrification, the present results reveal that this retained local coordination is accompanied by increased geometric disorder at the Cd sites and the formation of thermally induced paramagnetic defects.

Overall, these results provide an atomistic picture of vitrification in Cd-ZIFs, showing that the transition from crystal to glass involves subtle structural rearrangements that largely preserve the predominant Cd coordination environment while generating increased local geometric disorder and thermally induced paramagnetic defects. Although the microscopic nature of these defects remains unresolved, the combined experimental evidence indicates that glass formation involves structural changes extending beyond simple geometric disorder. Furthermore, the results demonstrate how subtle modifications in linker chemistry can profoundly alter the balance between melting, recrystallization, and glass formation in hybrid coordination networks, highlighting the importance of local coordination chemistry in understanding amorphization processes in hybrid framework materials.

Beyond their fundamental significance, the present findings may also contribute to the development of functional MOF glasses. A detailed understanding of the local coordination environment and defect formation is expected to facilitate the rational design of framework glasses with tailored properties, including optical and luminescent materials, ion-conducting glasses, and materials for sensing applications. More generally, establishing structure–property relationships in these amorphous coordination frameworks is expected to guide the development of new functional glassy materials.

## Supplementary Material



## Notes

Additional experimental details, FTIR complementary analysis, solid-state NMR complementary characterizations, liquid-state NMR characterizations, thermal behavior analysis, NMR cryoporometry and DFT calculations information, and supplementary figures (PDF)
