# Supplementary material for: Local Coordination Disorder and Defect Formation in Cadmium Zeolitic Imidazolate Framework Glasses
Source: Inorg Chem. 2026 Sep 24;65(39):23135–49. doi: 10.1021/acs.inorgchem.6c03271 (PMC13644304; doi:10.1021/acs.inorgchem.6c03271)
Supplement: Supplementary file 1 [file ic6c03271_si_001.pdf]

Supporting Information to

Local Coordination Disorder and Defect Formation in Cadmium Zeolitic Imidazolate Framework Glasses

*Renato Pereira Murback Filho,[1] Bruno Trebbi,[1] Ricardo José da Silva Afonso,[1] Camila Mizuki Ito Nishida,[1] Jonas Lins,[2] Gerd Buntkowsky,[2] Torsten Gutmann,[2,3] Eduardo Ribeiro deAzevedo,[1] Marcos de Oliveira Junior[1]**

[1] Sao Carlos Institute of Physics, University of Sao Paulo, IFSC – USP, 13566-590, Sao Carlos, SP, Brazil

[2] Eduard-Zintl-Institute of Inorganic and Physical Chemistry, Technical University of Darmstadt, Peter-Grünberg-Straße 8, Darmstadt D-64287, Germany

[3] Department of Chemistry, Physical Chemistry, University Paderborn, Warburger Straße 100, Paderborn D-33098, Germany

***Corresponding author:** mjunior@ifsc.usp.br

Contents

1. FTIR complementary analysis
2. Solid State NMR characterizations
3. ^{1}H liquid state NMR
4. Thermal behavior analysis
5. NMR Cryoporometry
6. Periodic DFT/GIPAW NMR Calculations for Nitrogen-Site Assignment in dia-$Cd(Im)_2$
7. SIMPSON Input file for simulation of REDOR curve

1. FTIR complementary analysis

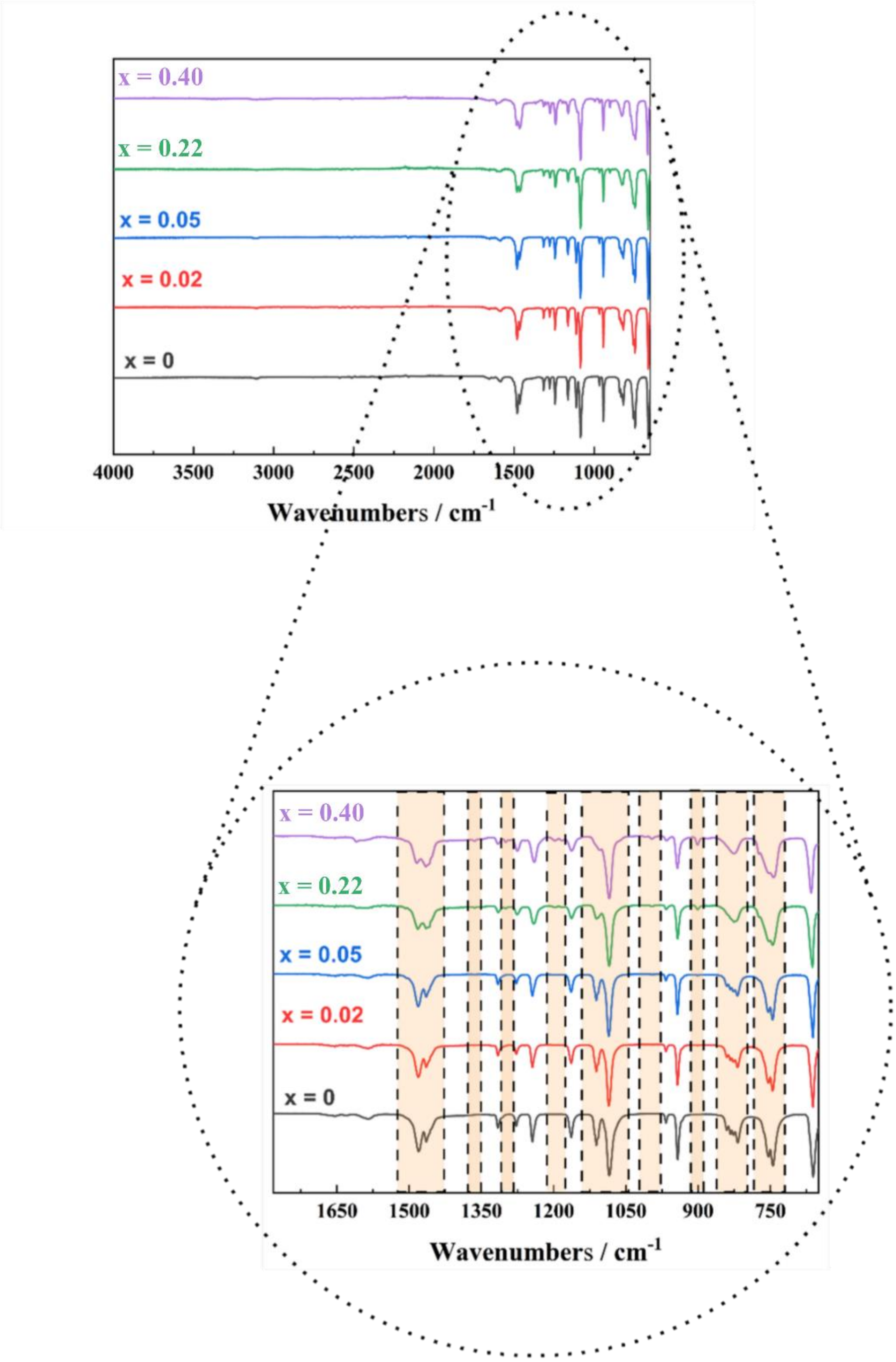

Figure S1. FTIR spectra of $Cd(Im)_{(2-x)}(bIm)_x$ samples.

Figure S1 shows the FTIR spectra of the $Cd(Im)_{(2-x)}(bIm)_x$ samples and an enlarged view in the range of 650 to 1780 cm^{-1}, where most of the bands are observed.

The spectra of all samples show the disappearance of the N–H stretching band at 3016 cm^{-1}, associated with the N–H bond of free imidazole, which revealed the deprotonation of imidazole and the subsequent coordination to cadmium ions.[1] The FTIR spectra also show bands at 3127 cm^{-1}, 1670 cm^{-1}, 1592 cm^{-1} and 1315 cm^{-1}, representing, respectively, the C-H, C=C, C=N and C-N stretching vibrations, respectively, characteristics compatible with the structural unit of imidazole, as reported by Bennett et al. for conventional ZIF-4,[1] indicating the structural integrity of the organic ligands.

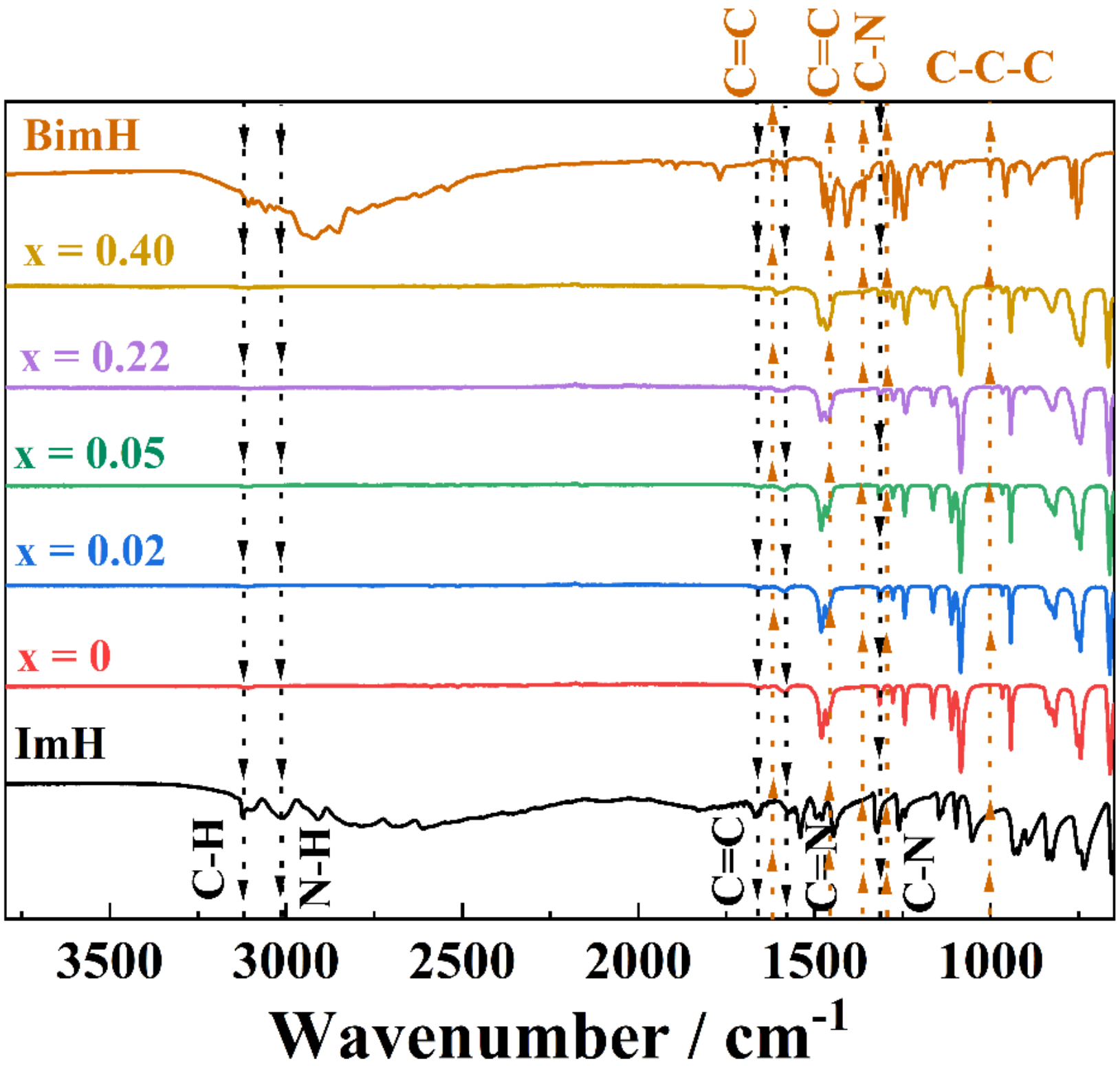

Figure S2. FTIR of the $Cd(Im)_{(2-x)}(bIm)_x$ samples compared with imidazole and benzimidazole precursors.

Figure S2 shows the FTIR spectra of pure benzimidazole and imidazole compared with the spectra of the $Cd(Im)_{(2-x)}(bIm)_x$ samples. As benzimidazole content increases, characteristic bands appear in the spectra, indicating structural integration. Characteristic benzimidazole bands corresponding to stretching vibrations of the C=C in the benzimidazole ring at 1618 and 1445 cm^{-1}, stretching vibrations of the C-N bond at 1360 and 1298 cm^{-1}, in-plane C-H bending at 1198 cm^{-1} and C-C-C trigonal bending at 1001 cm^{-1},[2] show an increase in intensity as benzimidazole content increases in the structure.

This result confirms the observation in the $^{13}C\{^{1}H\}$ CPMAS NMR spectra, which indicate benzimidazole coordination.

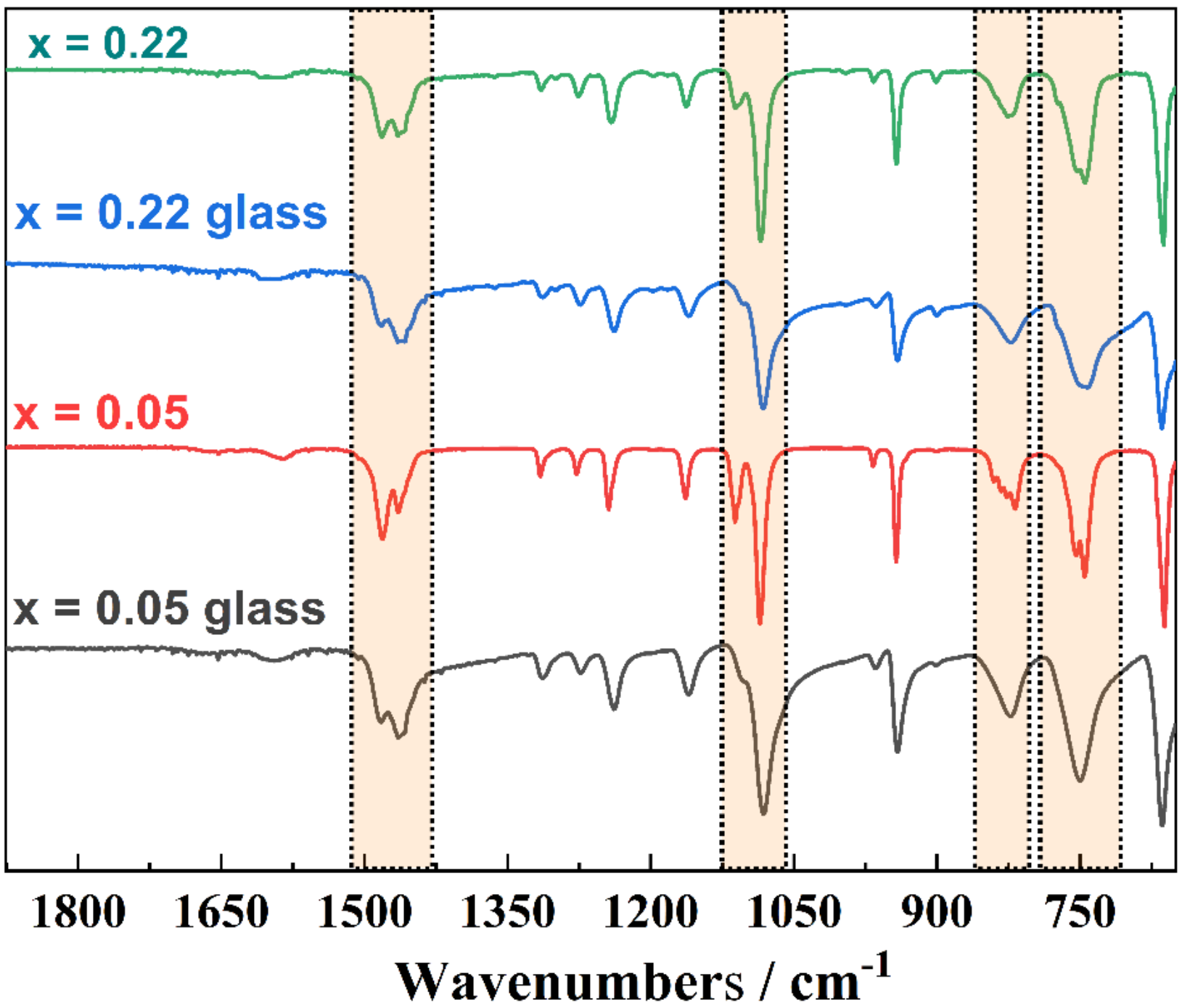

Figure S3. FTIR spectra of $Cd(Im)_{1.78}(bIm)_{0.22}$ and $Cd(Im)_{1.95}(bIm)_{0.05}$ as synthesized and in glass state.

Figure S3 shows the FTIR spectra of the $Cd(Im)_{1.78}(bIm)_{0.22}$ and $Cd(Im)_{1.95}(bIm)_{0.05}$ materials in both their as-synthesized and glassy states for comparison. Some characteristic bands undergo changes due to the vitrification process. The bands around 750 cm^{-1} and 825 cm^{-1} lose resolution due to the structural disorder typical of glassy materials. The band at 1112 cm^{-1} decreases in intensity in the glassy samples compared to the as-synthesized ones. In contrast, the C=C stretching band of benzimidazole at 1445 cm^{-1} shows increased intensity in the glassy materials relative to the as-synthesized counterparts.

2. Solid State NMR characterizations

^{1}H MAS spectra of $Cd(Im)_{(2-x)}(bIm)_x$

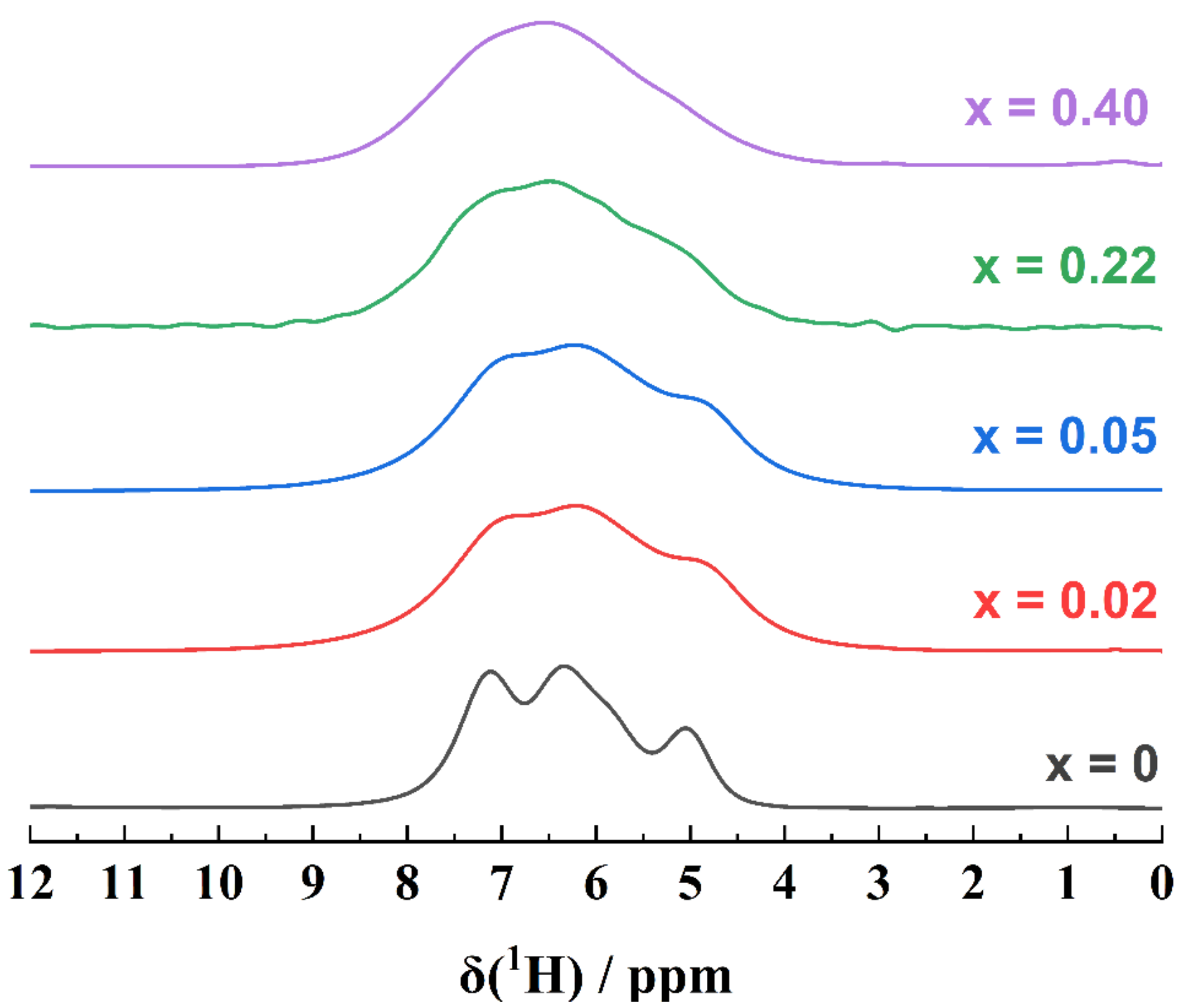

Figure S4. ^{1}H MAS NMR spectra of the $Cd(Im)_{(2-x)}(bIm)_x$ samples.

Figure S4 presents ^{1}H MAS NMR spectra of $Cd(Im)_{(2-x)}(bIm)_x$ for materials tested in this work (x = 0, x = 0.02, x = 0.05, x = 0.22 and x = 0.40). A loss of resolution is observed as benzimidazole content increases in the sample. The loss of ^{1}H short-range order accompanies loss of crystallinity in large-range order at higher benzimidazole contents.

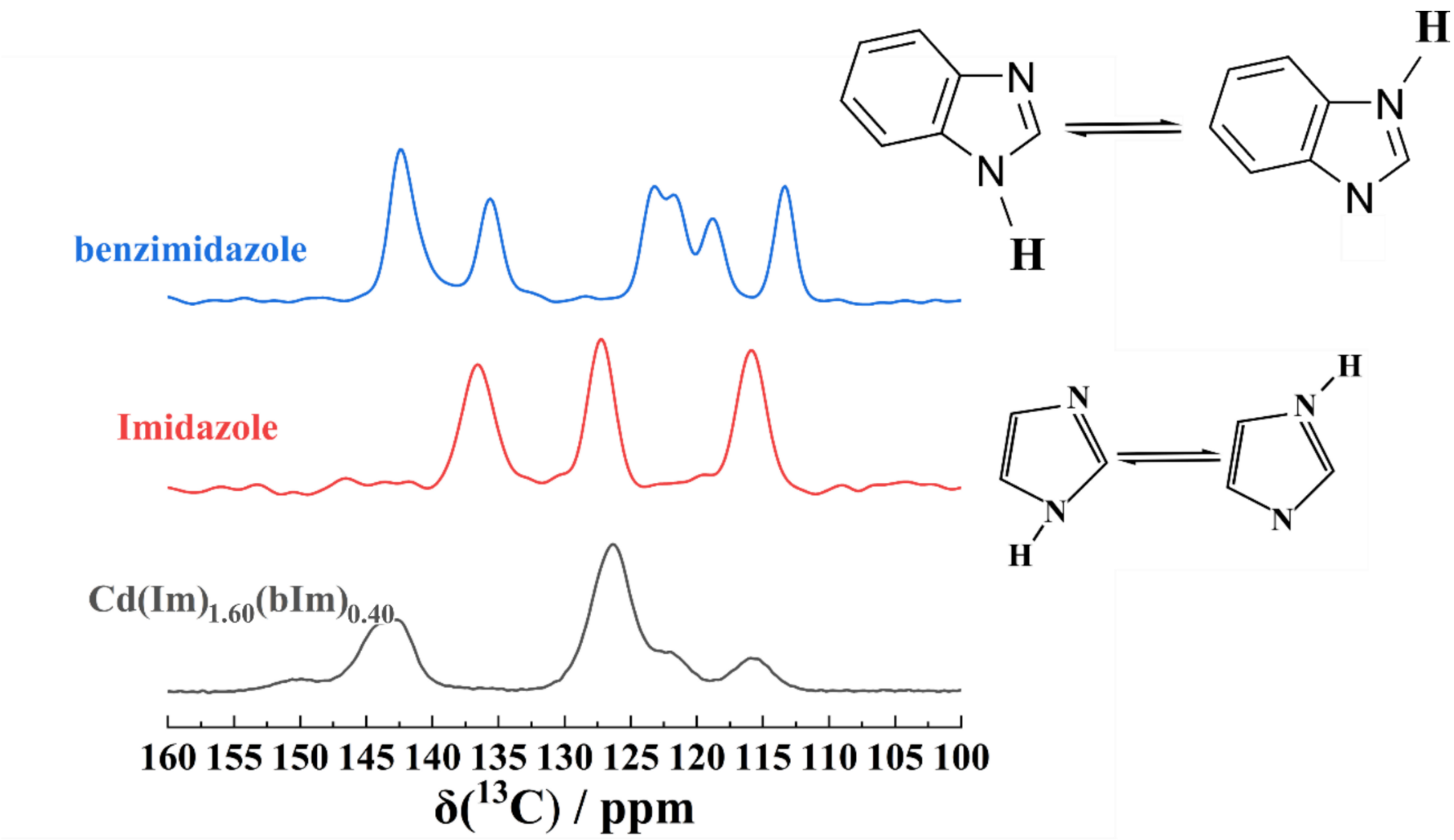

Figure S5. Comparation $^{13}C\{^1H\}$ CPMAS NMR spectra of $Cd(Im)_{1.60}(bIm)_{0.40}$, pure imidazole and benzimidazole. The inset shows the structures of the two tautomeric states of the ligand molecules Im and bIm, respectively.

Figure S5 shows the comparation between $^{13}C\{^1H\}$ CPMAS NMR spectra of $Cd(Im)_{1.60}(bIm)_{0.40}$, pure imidazole (Im) and benzimidazole (bIm). The two tautomeric states are also shown in the figure. The spectrum of pure imidazole exhibits three distinct peaks, resulting from the coexistence of two tautomeric forms of the molecule. This tautomerization involves the redistribution of double bonds and occurs quickly in solution. However, in the solid state, this dynamic is significantly reduced, resulting in the observation of two well-defined states in the ^{13}C spectrum. The same applies to benzimidazole, explaining the spectrum shape. In the $Cd(Im)_{1.60}(bIm)_{0.40}$ spectrum, the tautomeric states have collapsed into well-defined peaks of coordinated imidazole and benzimidazole ligands.

^{113}Cd spectra simulations

The chemical shift anisotropy powder pattern simulations for the ^{113}Cd spectrum were performed using the ssNake software.[3]

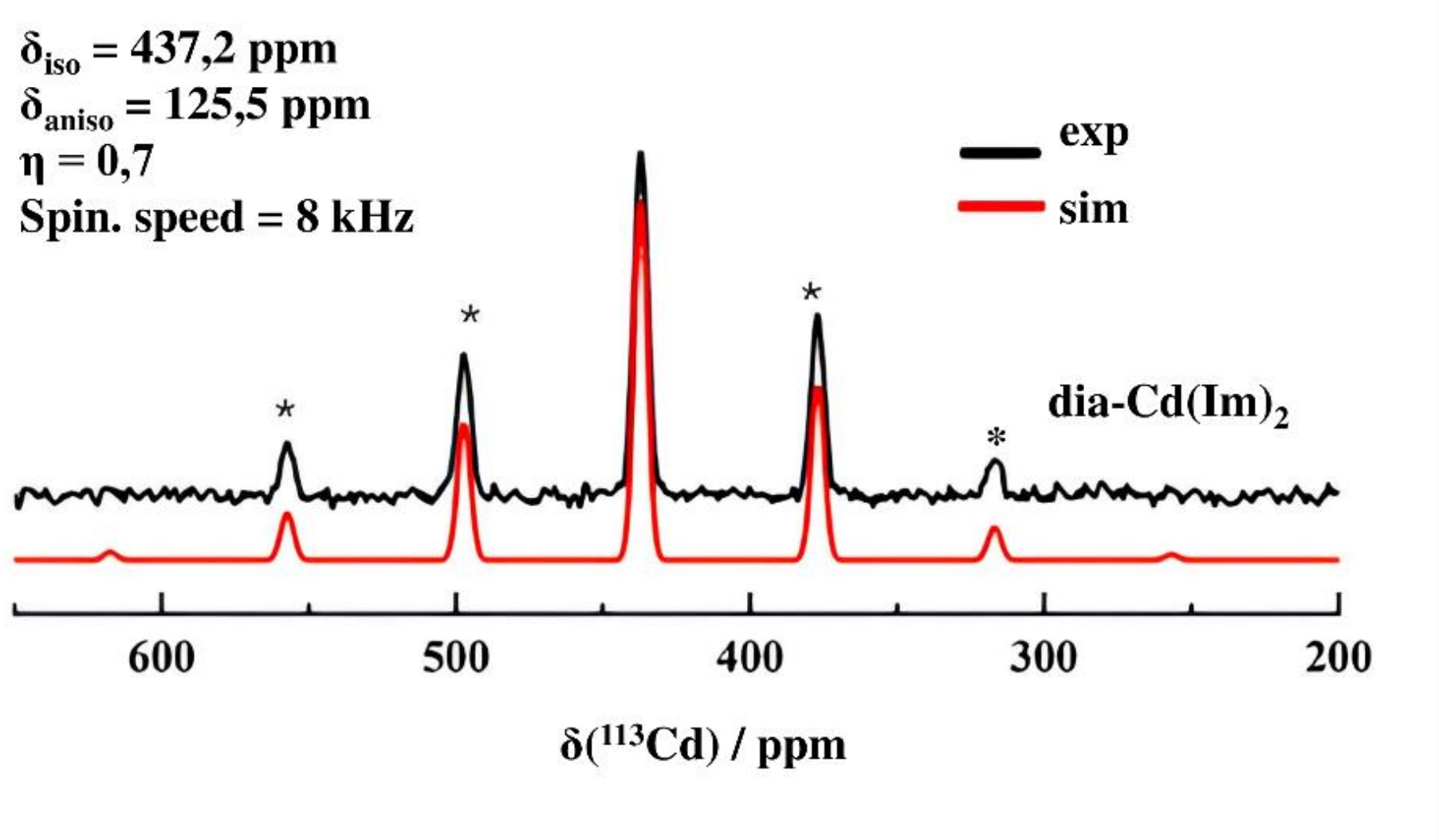

Figure S6. dia-$Cd(Im)_2$ ^{113}Cd spectrum simulation.

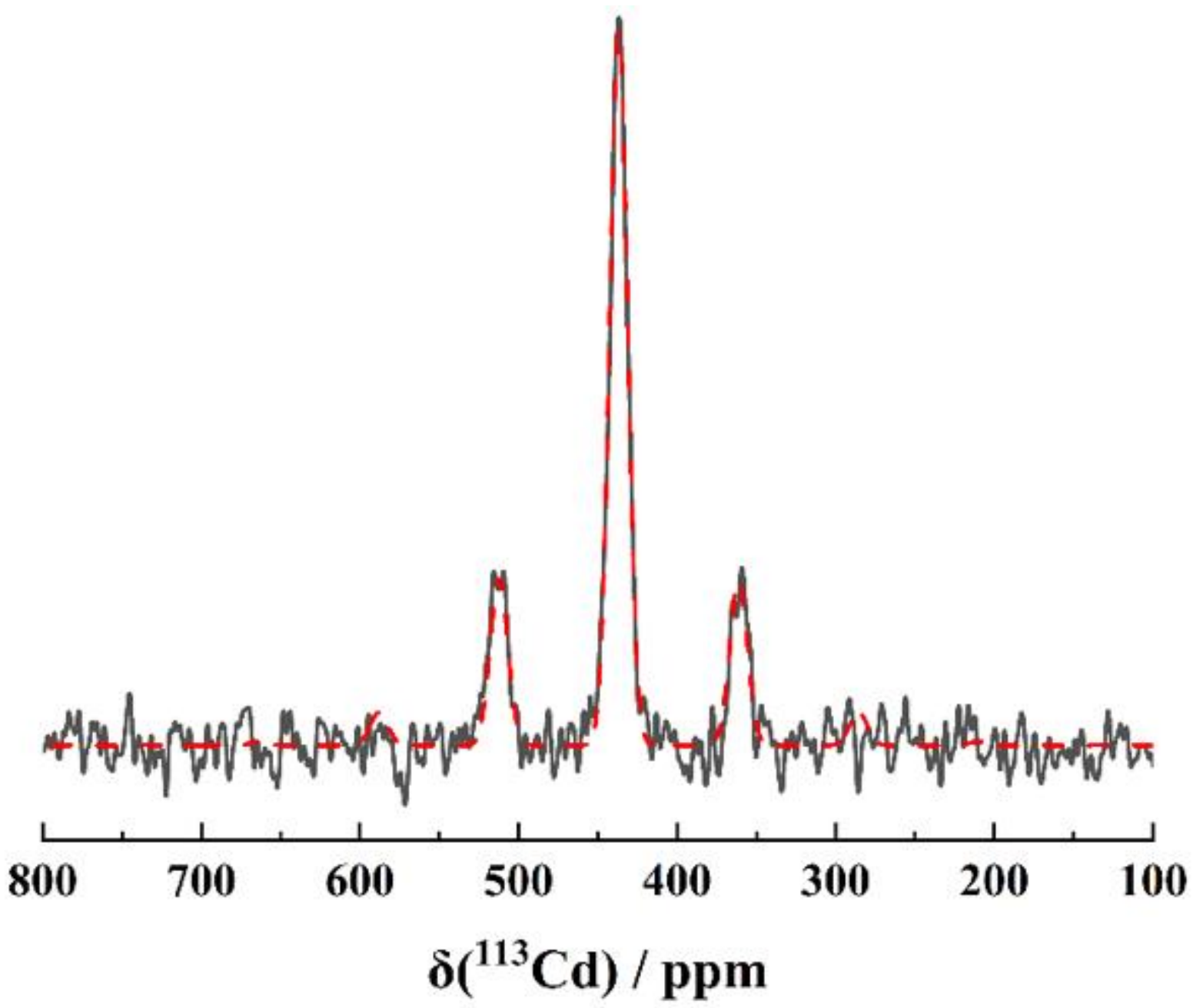

Figure S7. $Cd(Im)_{1.98}(bIm)_{0.02}$ ^{113}Cd spectrum simulation.

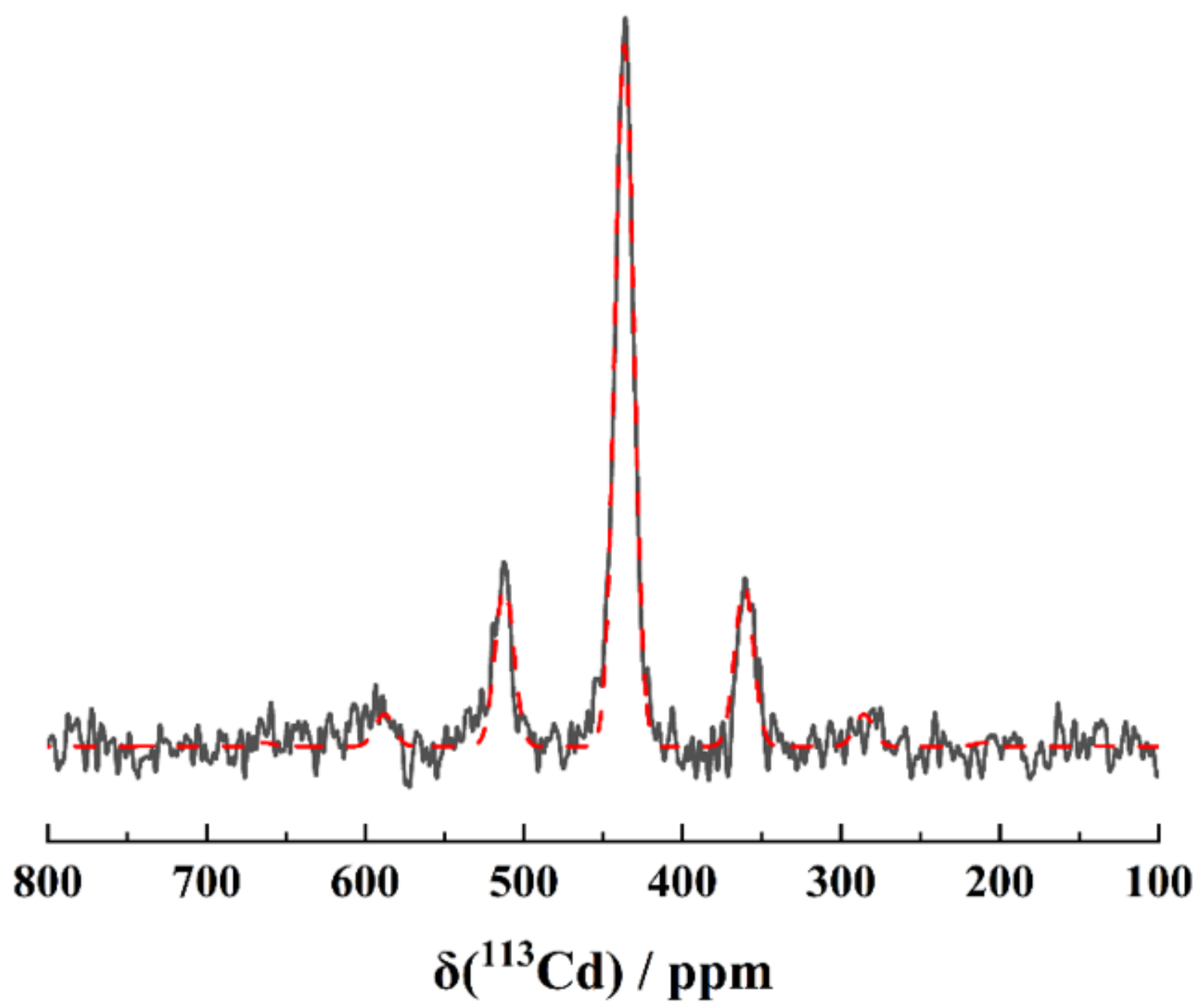

Figure S8. Cd(Im)$_{1.95}$(bIm)$_{0.05}$ ^{113}Cd spectrum simulation.

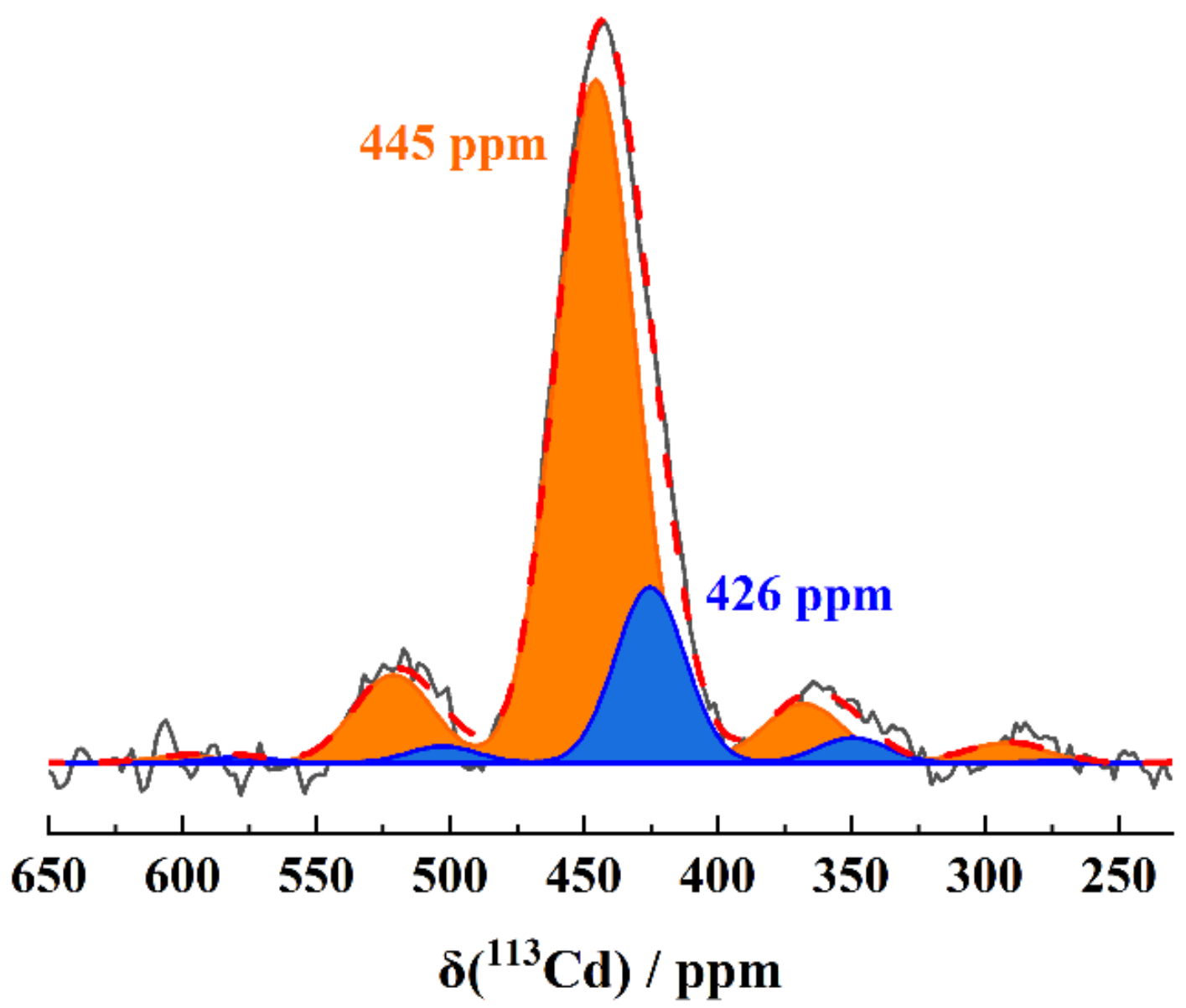

Figure S9. Cd(Im)$_{1.95}$(bIm)$_{0.05}$ glass ^{113}Cd spectrum simulation.

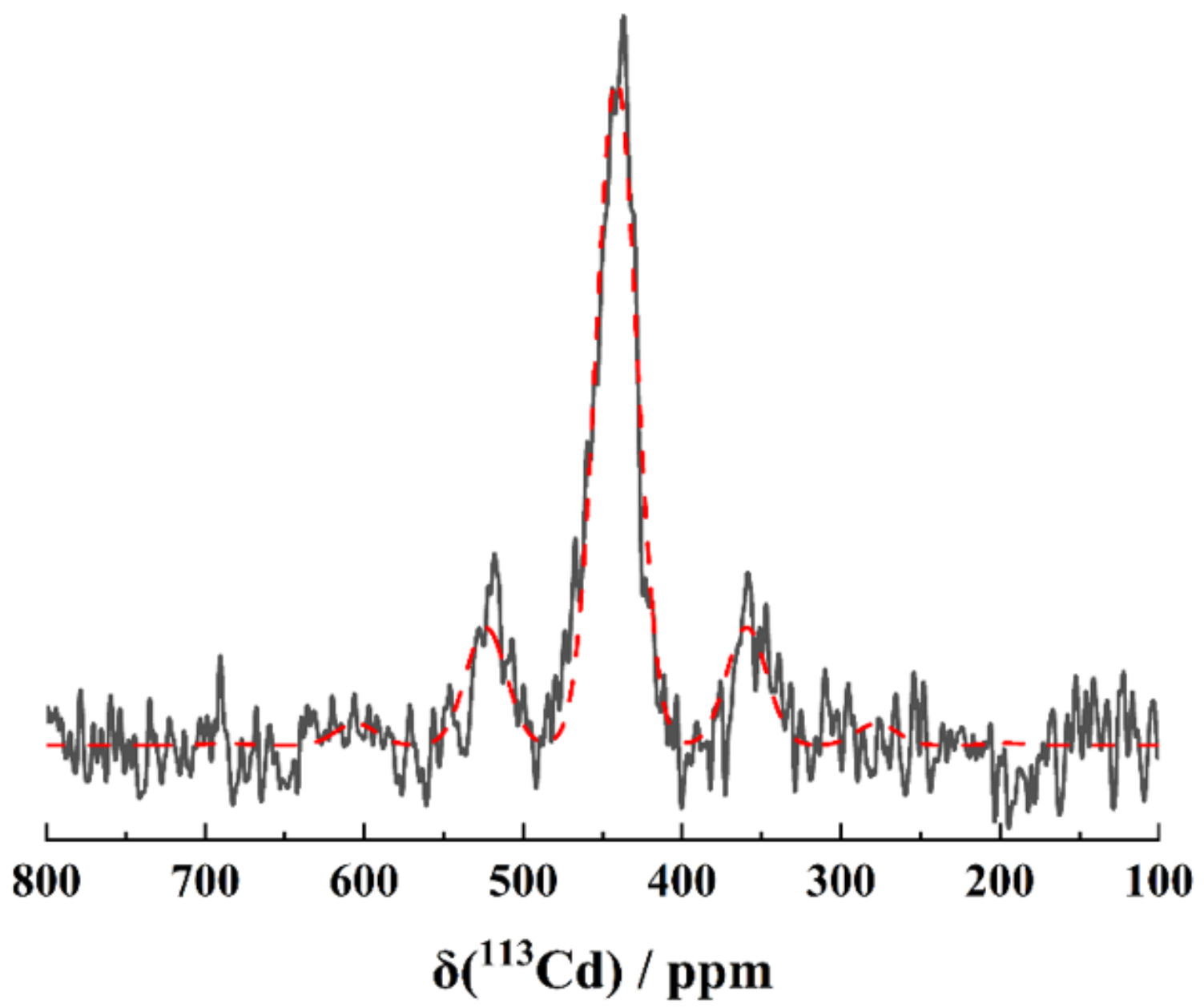

Figure S10. $Cd(Im)_{1.78}(bIm)_{0.22}$ ^{113}Cd spectrum simulation.

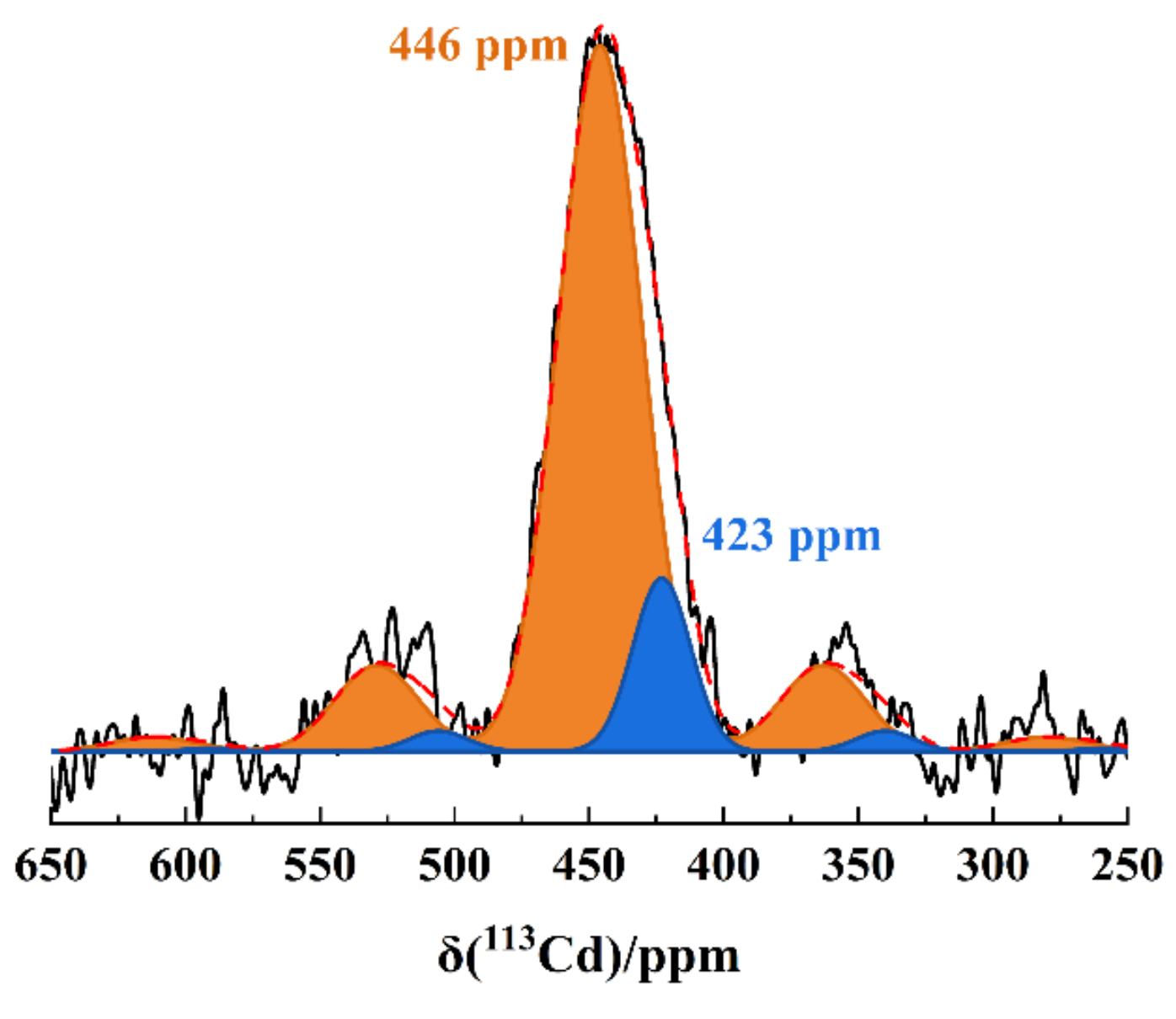

Figure S11. $Cd(Im)_{1.78}(bIm)_{0.22}$ glass ^{113}Cd spectrum simulation.

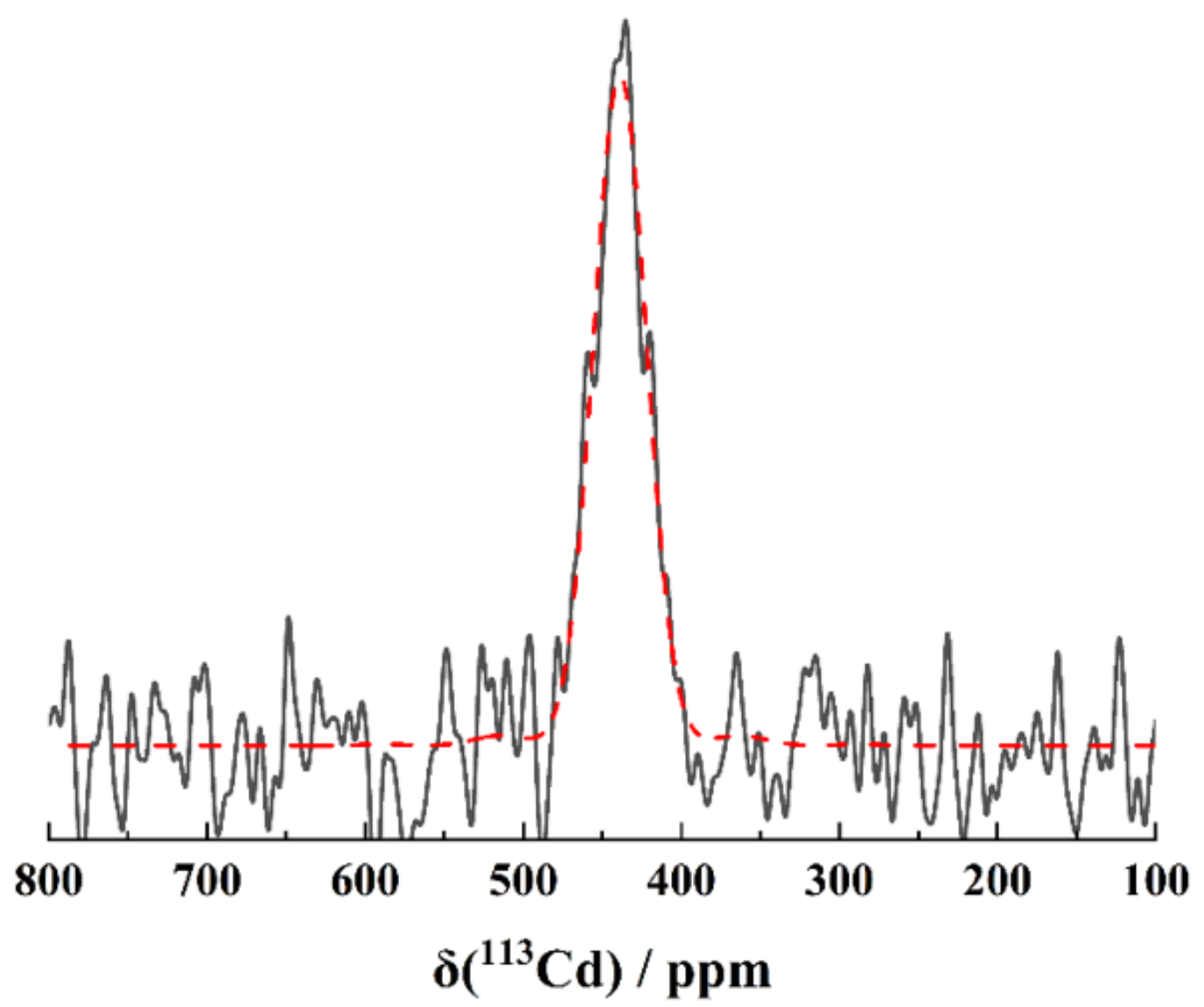

Figure S12. $Cd(Im)_{1.60}(bIm)_{0.40}$ ^{113}Cd spectrum simulation.

Table S1. ^{113}Cd chemical shift tensor parameters

**As-synthesized materials**					
**$Cd(Im)_{(2-x)}(bIm)_x$**	δ_{iso} /ppm	δ_{aniso} /ppm	η	Integral	Gaus / ppm
**x = 0**	437.2	125.5	0.7	1672	5.55
**x = 0.02**	436.8	113.8	1	1430	13.2
**x = 0.05**	436.7	112.0	1	1481	14
**x = 0.22**	441.4	108.5	1	2748	28.9
**x = 0.40**	438.8	30.9	1	2660	39.4
**Glasses**					
**glass $Cd(Im)_{(2-x)}(bIm)_x$**	δ_{iso} /ppm	δ_{aniso} /ppm	η	Integral	Gaus / ppm
**x = 0.05**	445	78.4	1	2820	35.96
	426	130.2	1	998.8	40.97
**x = 0.22**	446	93.1	1	3440.0	38.33
	423	128	0.5	655.9	27.52

^1H Hahn echo NMR spectra with variation temperature.

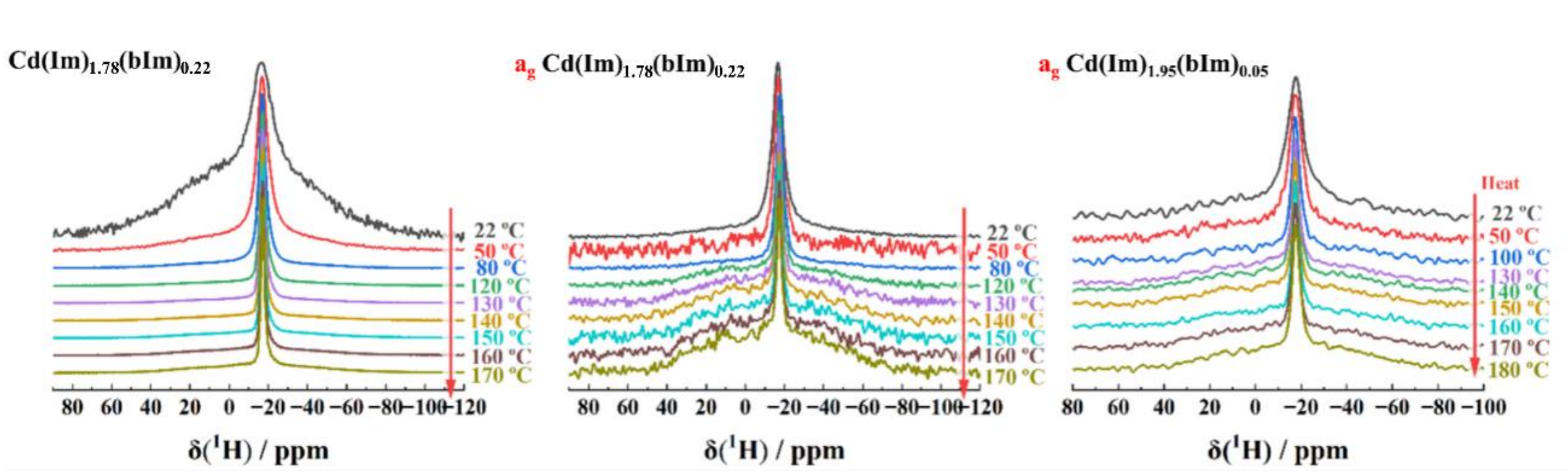

Figure S13. ^1H Hahn echo NMR spectra of as-synthesized $Cd(Im)_{1.78}(bIm)_{0.22}$, $Cd(Im)_{1.78}(bIm)_{0.22}$ glass and as synthesized $Cd(Im)_{1.95}(bIm)_{0.05}$ glass. a_g = glass state.

Variable-temperature static ^1H NMR spectra were recorded at a magnetic field of 5.64 T using an Agilent DD2 spectrometer equipped with a 4.0 mm probe. The spectra were acquired under static conditions using a Hahn-echo pulse sequence (π/2–τ–π–τ–acquisition). A π/2 pulse length of 7.7 μs and an interpulse delay (τ) of 200 μs were employed, with up to 128 scans accumulated for each spectrum. Measurements were performed at 22, 50, 80, 120, 130, 140, 150, 160, and 170 °C. Figure S13 presents the static ^1H NMR spectra as a function of temperature for the as-synthesized and glass samples with x = 0.22, as well as for the glass sample with x = 0.05. The spectra consist of two distinct components: a broad resonance and a narrower resonance. Both components evolve with increasing temperature, exhibiting changes in linewidth and relative intensity. However, the broad component does not display a clear or systematic temperature dependence. Therefore, the analysis was focused on the narrow component, whose spectral evolution provides a more reliable probe of the temperature-dependent proton dynamics.

2D Heteronuclear correlation (HETCOR) ^1H-^{13}C experiment

For the assignment of the ^1H and ^{13}C sites in the solid-state NMR spectra, 2D heteronuclear correlation (HETCOR) ^1H-^{13}C measurements were performed.

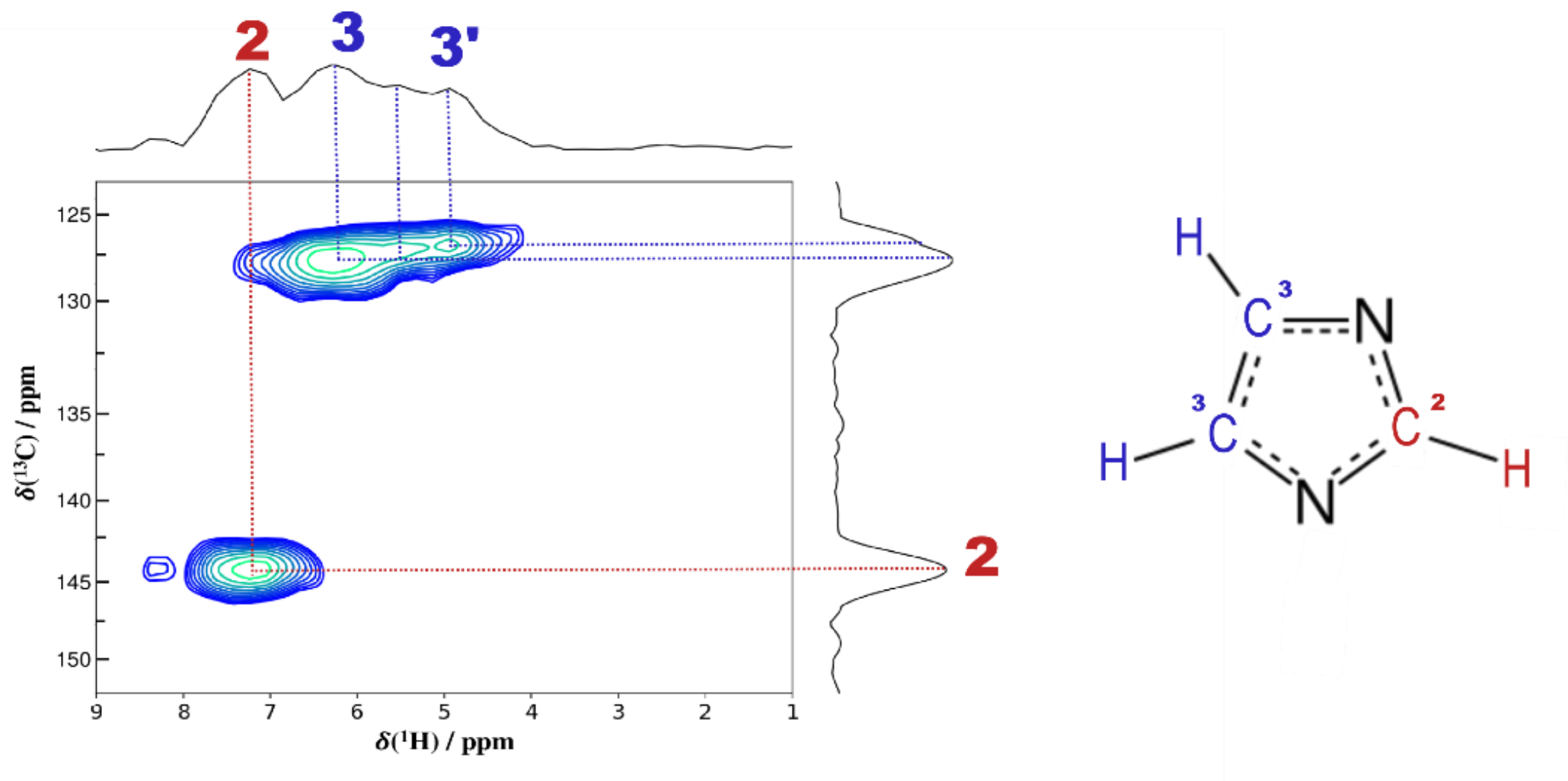

Figure S14. $^1H\{^{13}C\}$ HETCOR spectra of the dia-Cd(Im)$_2$. The spectra were measured at 60 kHz MAS and a contact time of 2 ms.

Heteronuclear correlation (HETCOR) spectra were measured at 60 kHz MAS with ^1H detection, using a double-CP pulse sequence.[4] Chemical shifts are reported relative to tetramethylsilane at 0 ppm for all isotopes, using α-glycine as a secondary reference ($^{13}C[\delta(C{=}O)]$ = 176.5 ppm and $^1H[\delta(NH_3)]$ = 8.5 ppm). The $^1H\{^{13}C\}$ HETCOR spectrum acquired with fast MAS (60 kHz) and proton detection for the dia-Cd(Im)$_2$ is shown in Figure S14. The experiment was carried out using a relatively long contact time of 2 ms to probe the H-C spatial proximity through the ^1H-^{13}C heteronuclear dipolar coupling. The proton resonance at 7.2 ppm shows a strong correlation with the ^{13}C signal at 146.0 ppm, assigned to the NCN carbon site (2). The proton with signals at 6.7 ppm (3) and 5.1 ppm (3') correlate with the ^{13}C signals around 128 ppm, which are assigned to the NCC carbon sites. These correlations are consistent with the bonding arrangement within the imidazolate ring and support the assignment of the chemically distinct proton and carbon environments.

2D Homonuclear correlation (SQ-DQ) ^1H-^1H experiment

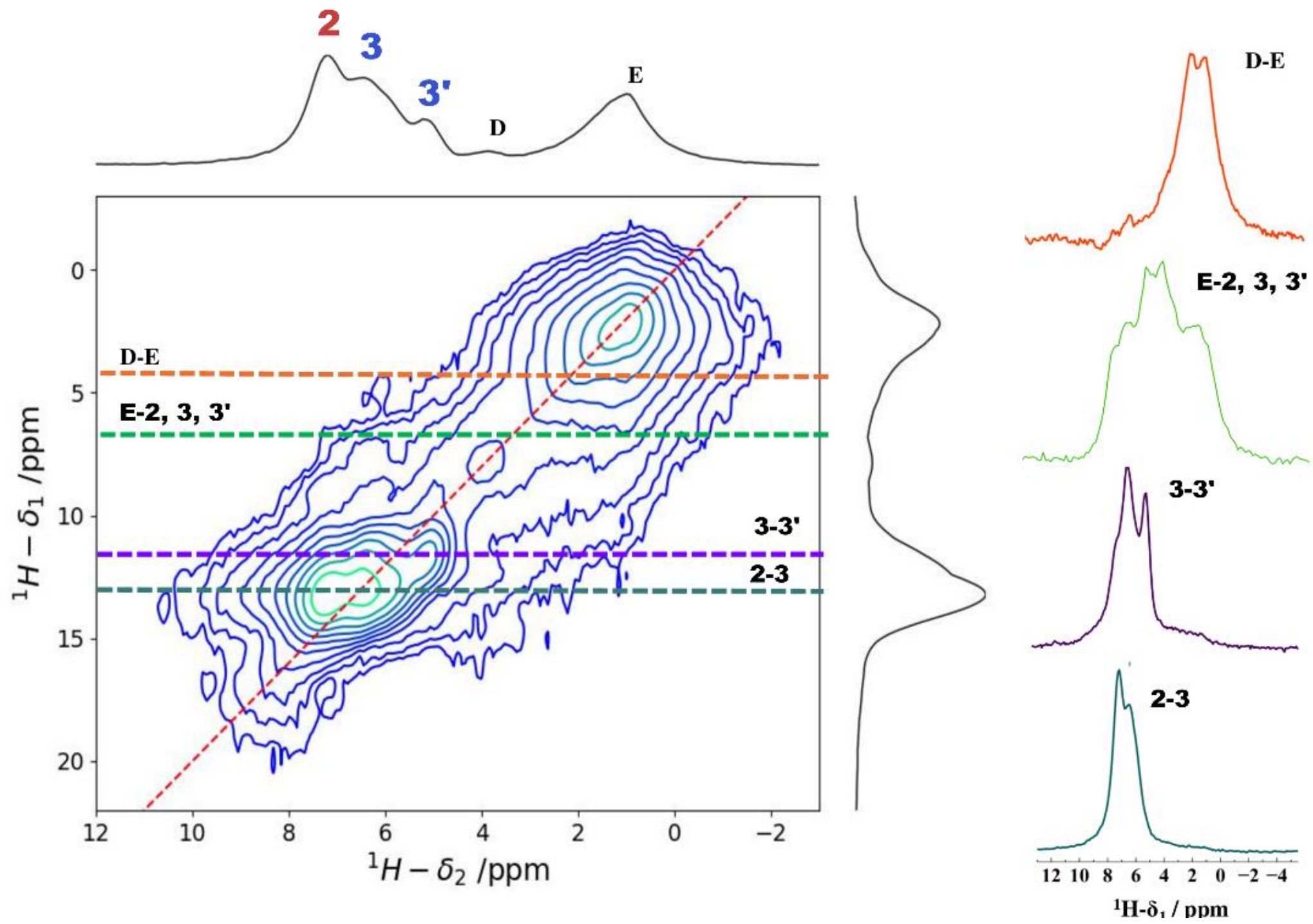

Figure S15. dia-Cd(Im)$_2$ 2D ^1H–^1H SQ-DQ NMR.

^1H double quantum–single quantum (DQ–SQ) experiments were performed under 60 kHz MAS using an $R14_4^5$ symmetry-based homonuclear recoupling scheme with no homonuclear decoupling during DQ evolution.5 Figure S15 shows the two-dimensional homonuclear 1H–1H correlation spectrum (SQ-DQ) of dia-Cd(Im)2 and the observed correlations between the sites. At approximately 13.0 ppm on the DQ scale (δ1), two peaks can be observed that indicate the interaction between sites 2 (7.2 ppm) and 3 (6.7 ppm), corresponding to protons belonging to imidazole. It is also possible to observe, at approximately 12.0 ppm on the DQ scale (δ_1), the interaction between sites 3 (6.7 ppm) and 3' (5.1 ppm), which are also hydrogen atoms of the imidazole unit. This corroborates the proximity of the protons that were assigned to the imidazole molecule. At approximately 7.0 ppm on the DQ scale (δ_1), interactions can be observed between pairs involving site E (1.1 ppm) and the hydrogens of the imidazole unit. These interactions are of low intensity, indicating that although there is still some spatial proximity, these units are more distant compared to the strong interactions observed between imidazole molecules themselves. This suggests that the species at 1.1 ppm is present in peripheral regions of the framework or has lower structural coupling with dia-Cd(Im)$_2$. Additionally, at approximately 4.0 ppm on the DQ scale (δ_1), it is possible to observe an interaction

between site D (3.8 ppm) and site E (1.1 ppm), indicating proximity between the species, which most probably belong to methanol molecules trapped in the pores of the material.

3. ^{1}H liquid state NMR

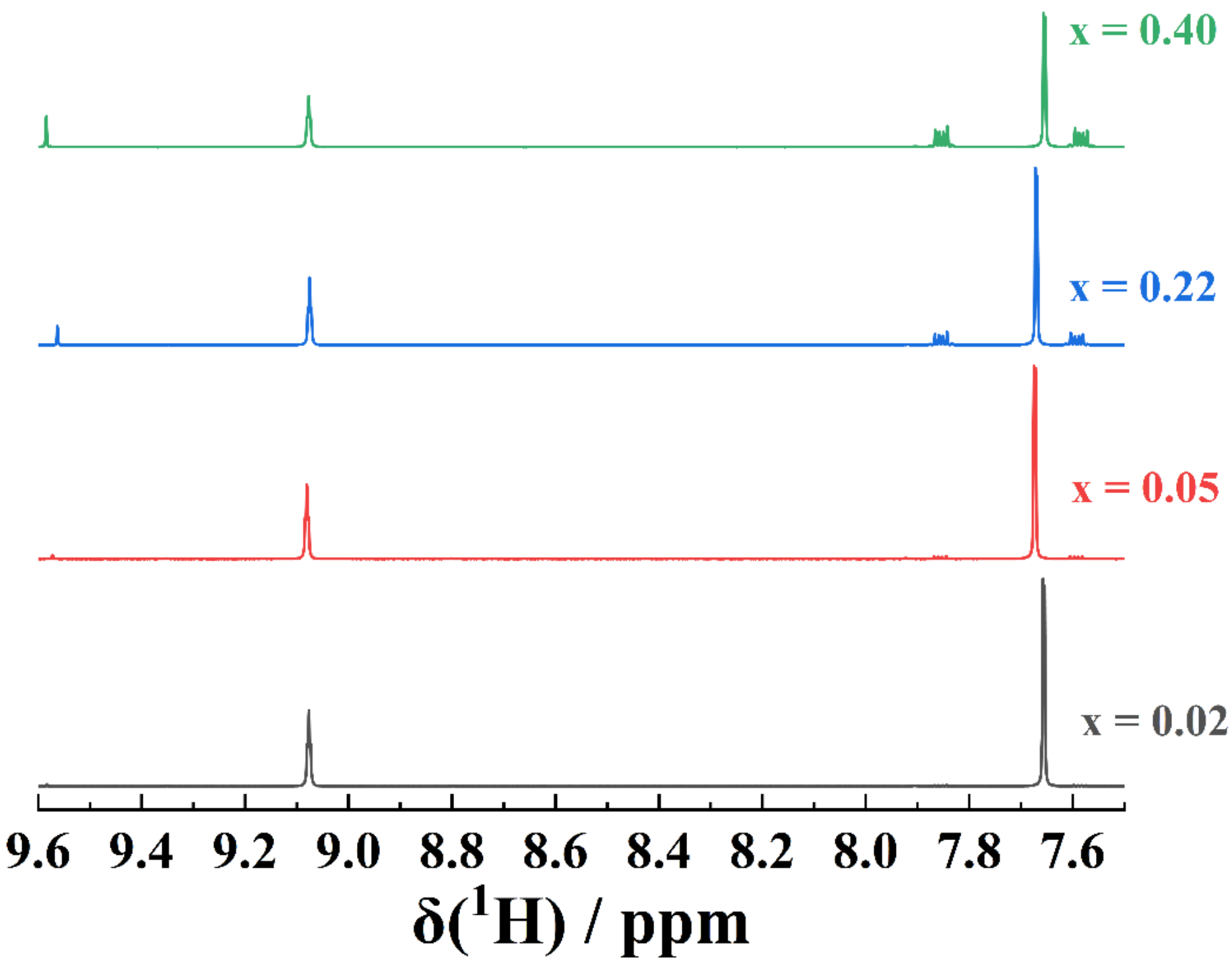

Figure S16. ^{1}H liquid state NMR spectra samples x = 0.02, x = 0.05, x=0.22 and x=0.40.

To quantify the Im/bIm ratio in each sample of the Cd(Im)$_{2-x}$(bIm)$_x$ serie, we performed ^{1}H liquid-state NMR experiments. Figure S16 shows the ^{1}H spectra for all samples, as well as the assignments for each resonance peak corresponding to the sites of the imidazole and benzimidazole molecules. Table S2 below presents the normalized integral values for each site of the measured samples.

Table S2. integral values of each normalized ^{1}H liquid state NMR resonance peak.

	2a	1a	2b	1b	2c
**0.40**	0.40	1.60	0.91	3.53	0.95
**0.22**	0.22	1.78	0.49	3.9	0.52
**0.05**	0.05	1.95	0.11	4.28	0.13
**0.02**	0.02	1.98	0.06	4.24	0.07

4. Thermal behavior analysis

TGA analysis

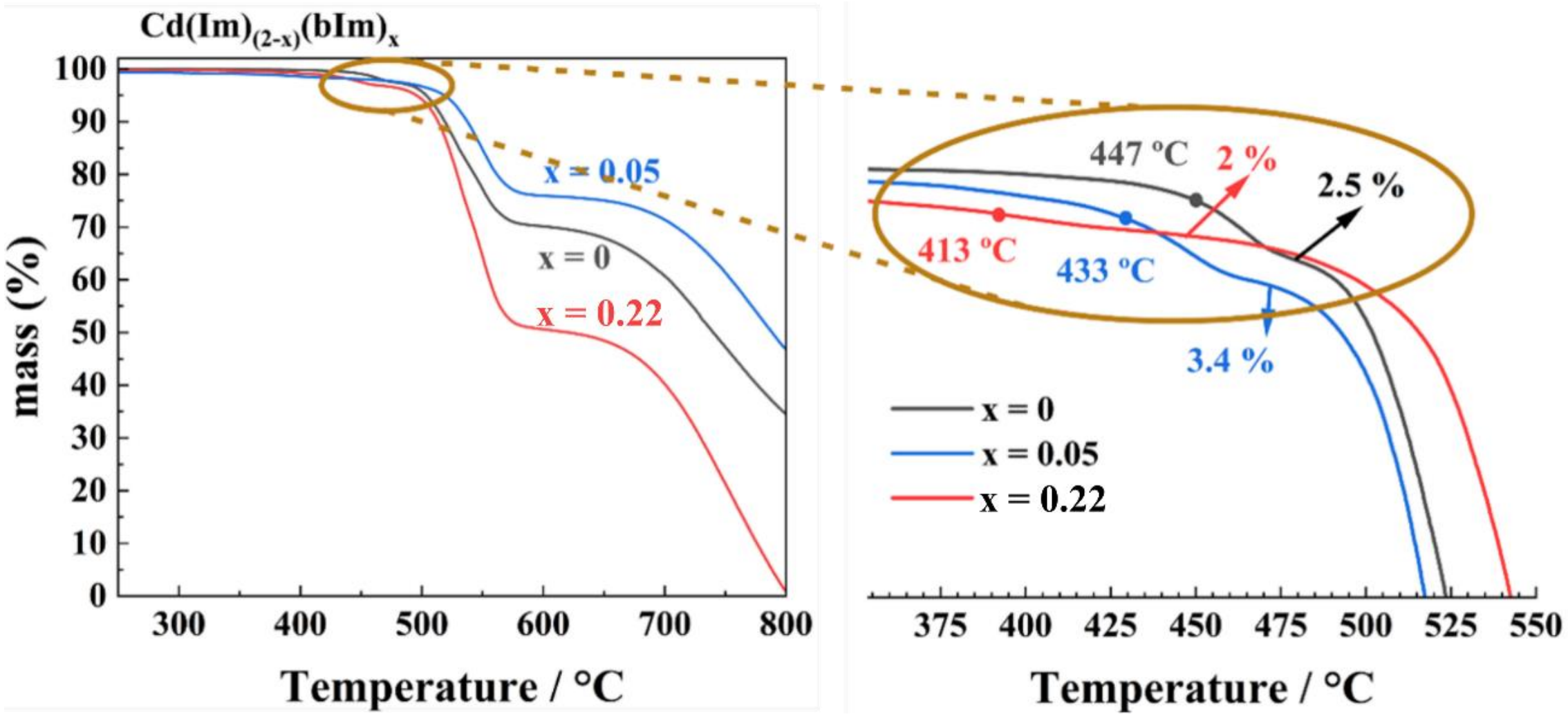

Figure S17. TGA analysis of samples x = 0, x = 0.05 and x=0.22.

For x = 0, thermogravimetric analysis (TGA) (Figure S16) indicates that the thermal decomposition occurs close to the melting temperature (447 °C). A small weight loss of approximately 2.5% is observed, corresponding to a partial decomposition. Around 500 °C, a second decomposition stage occurs, resulting in a 30% weight loss. Finally, at approximately 660 °C, a third and almost complete mass loss is observed. The same decomposition behavior is observed for the three samples. For x = 0.05, the first decomposition temperature decreases to 433 ºC, and the weight loss is approximately 3.4 %. For x = 0.22, decomposition temperature at first decreases to 413 ºC and the weight loss is approximately 2 %. The second step occurs at a similar temperature range for the three samples, although the associated weight loss varies among the samples.

Table S3. Thermal events of $Cd(Im)_{2-x}(bIm)_x$ system.

$Cd(Im)_{2-x}(bIm)_x$	Melting event		Glass Temperature (T_g) / °C	Crystallization (T_c) / °C	
	Temperature T_m / °C	Enthalpy / J/g		Temperature / °C	Enthalpy / J/g
**x = 0**	452	-89.85	-	-	-
**x = 0.02**	446	73.17	-	-	-
**x = 0.05**	440	-67.71	144	187	42.3
**x = 0.22**	404	-37.32	153	344	19.1
**x = 0.40**	368	-33.37	165	-	-

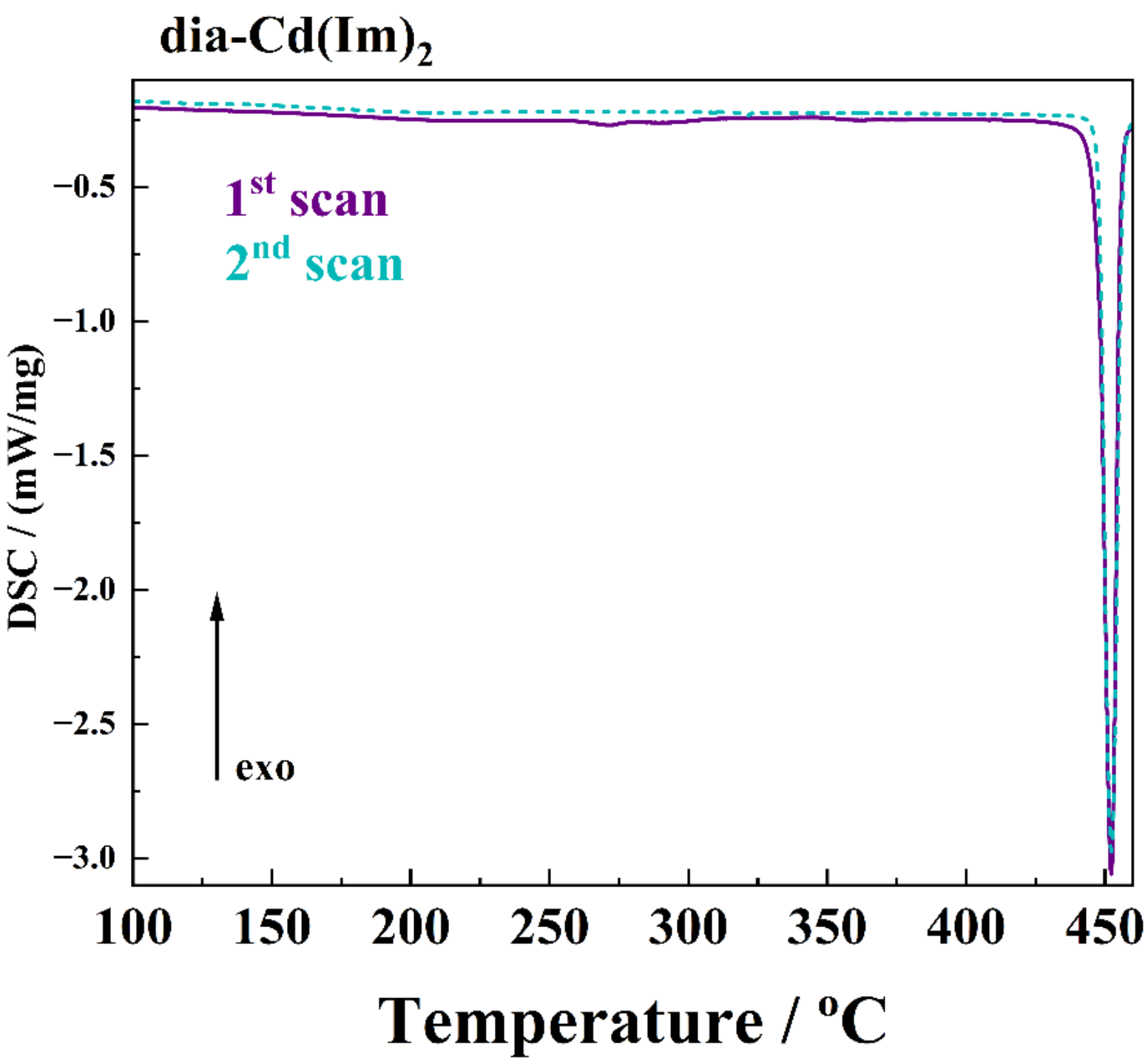

Figure S18. DSC profiles dia-$Cd(Im)_2$.

The melting endothermic peak of dia-$Cd(Im)_2$ was observed at 452 °C in the first upscan. The corresponding melting enthalpy was -89.85 $J \cdot g^{-1}$. In the second heating cycle, no glass transition event was observed, whereas metallic cadmium was identified (321 °C). The Cd^0 content was estimated to be approximately 0.17 wt% based on the ratio

between the enthalpy of the cadmium melting event and the total melting enthalpy of the material.

DSC analysis of $Cd(Im)_{1.78}(bIm)_{0.22}$ crystallization behavior.

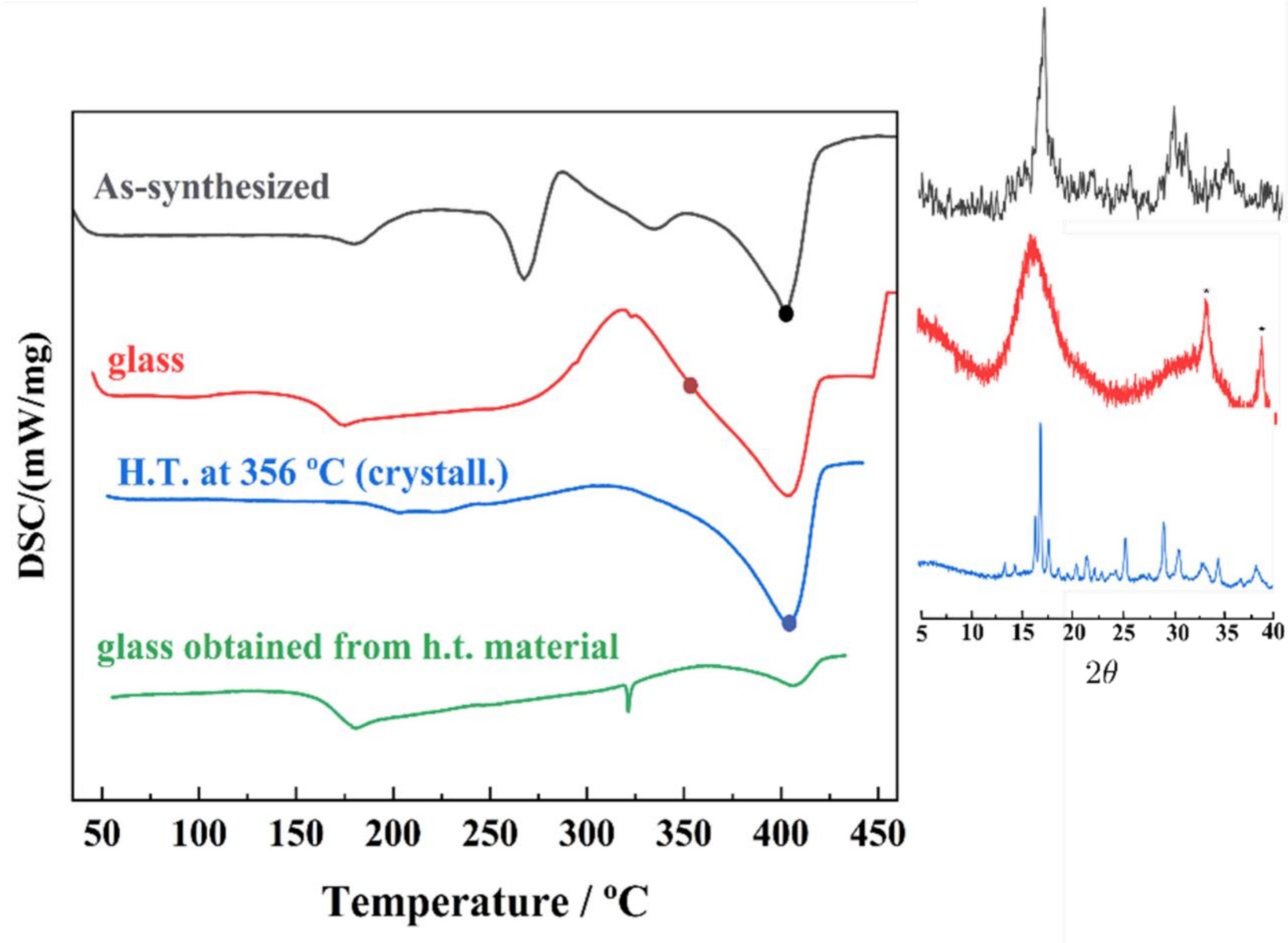

Figure S19. DSC analysis of $Cd(Im)_{1.78}(bIm)_{0.22}$ as sythesized, glassy, heat treated at crystallization peak and the glass obtained from crystallized material.

Figure S17 shows the DSC curves of as-synthesized $Cd(Im)_{1.78}(bIm)_{0.22}$, the corresponding glass, the material heat-treated at the crystallization peak, and the glass obtained after remelting the crystallized material. As discussed above, this is the first ZIF glass reported to exhibit recrystallization behavior. To investigate this phenomenon, the glass was heat-treated at 355 °C for 20 min, corresponding to the end of the glass crystallization peak. The DSC curve of the heat-treated (H.T.) material still exhibits a weak Tg, indicating that a fraction of the sample remained amorphous and did not crystallize during the treatment. Since most of the material recrystallized, the crystallization peak becomes significantly less intense. Interestingly, the XRD pattern of the H.T. material indicates a tendency toward crystallization into the dia-$Cd(Im)_2$

structure, which does not contain benzimidazolate linkers, unlike the $Cd(Im)_{1.78}(bIm)_{0.22}$ precursor. The as-synthesized material forms an amorphous solid because the incorporation of benzimidazolate is incompatible with the dia framework. However, upon heating, the glass tends to recrystallize into the dia-$Cd(Im)_2$ phase. The recrystallized material was subsequently melted and rapidly cooled to room temperature. Glass formation was again observed, as evidenced by the presence of a Tg at a temperature similar to that of the original glass (green curve). The slight difference between the Tg values of the two glasses may arise from variations in the cooling rate during each thermal cycle, since the glass transition temperature depends on kinetic factors. The melting peak associated with metallic cadmium becomes more intense after thermal cycling, suggesting partial decomposition during each cycle. In contrast, the crystallization and framework melting events nearly disappear, which may also be attributed to progressive decomposition occurring throughout the thermal treatment.

Powder X-ray Diffraction

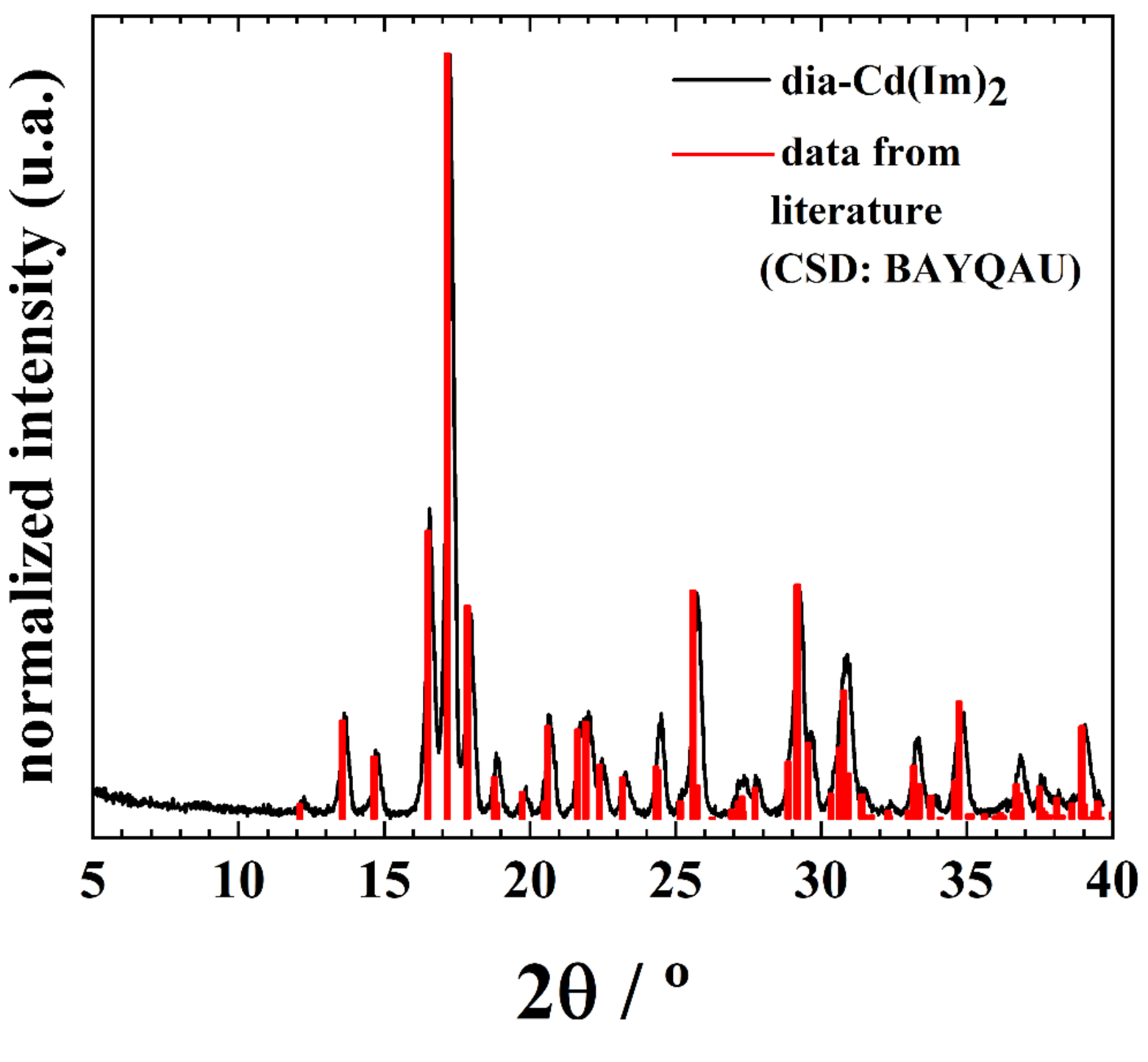

Figure S20. Powder X-ray Diffraction of dia-$Cd(Im)_2$ (x = 0) compared to reported in literature.

dia-Cd(Im)$_2$ synthesized by a solution-precipitation route has a crystalline phase with good agreement with data reported in the literature (BAYQAU). This structure crystallizes in a two-fold interpenetrated diamond topology, forming a cadmium tetrahedral environment coordinated by four imidazole linkers.

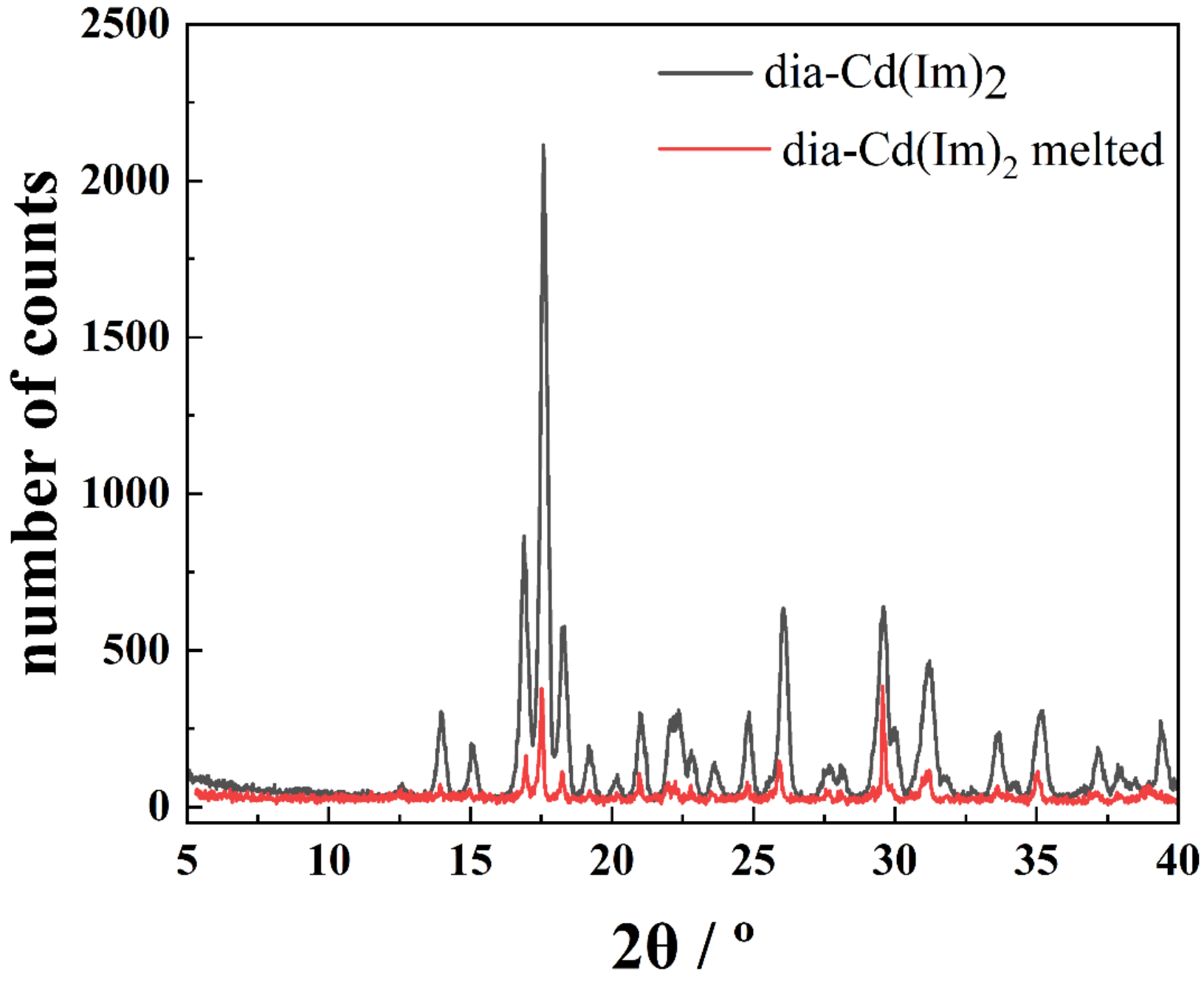

Figure S21. Comparation of Powder X-ray Diffraction pattern of dia-Cd(Im)$_2$ (x = 0) as-synthesized and after a Heat treatment of melt and cooling.

After melting and subsequent cooling, dia-Cd(Im)$_2$ synthesized by our chemical route did not exhibit a complete glass formation. XRD shows a decrease in the number of counts of the heat-treated material, maybe due to partial decomposition that can form an amorphous carbon structure decorated by metallic particles. However, crystalline characteristics of the material that did not decompose are preserved in the diffractogram.

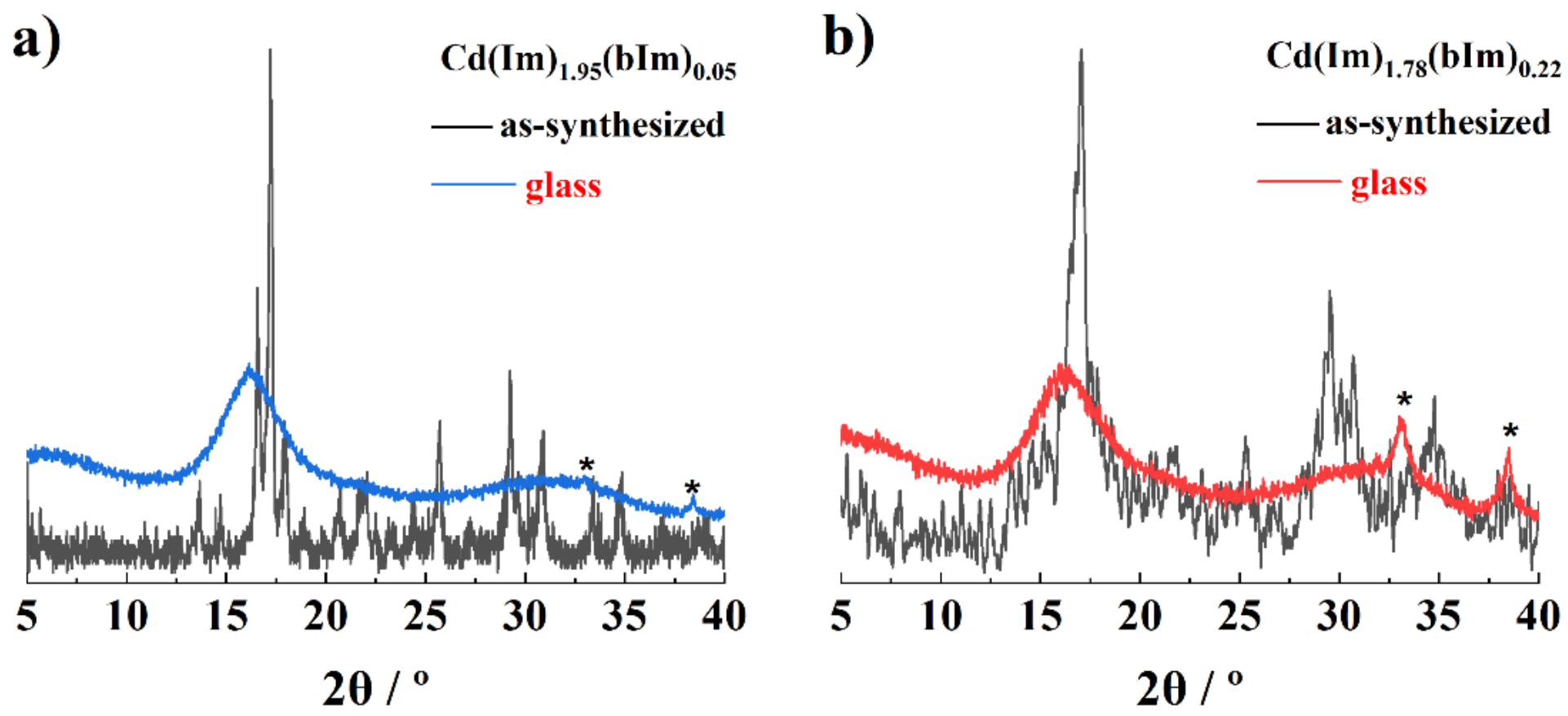

Figure S22. Powder XRD pattern of **a)** Sample x = 0.05 and **b)** x = 0.22 as-synthesized and in the glassy state. Asterisks indicate CdO signal.

Figure S19 shows the Powder XRD pattern of samples x = 0.05 and x = 0.22 as-synthesized and in a glassy state. Glasses are characterized by a complete non-crystalline pattern, with a diffuse halo in the region of the crystalline peaks of the material as-synthesized, which indicates a structural memory of the crystal precursor in the glass. Formation of CdO can be observed in both glasses, which may be due to the partial decomposition suffered during the vitrification. A higher CdO signal is observed for the x = 0.22 glass.

5. NMR Cryoporometry

Cryoporometry is based on detecting variations in the melting/freezing temperature of fluids confined within pores, as described by the Gibbs–Thomson equation, which relates the shift in transition temperature to changes in the Helmholtz free energy. When a liquid is confined within a pore, its interaction with the pore surface alters the Helmholtz free energy and consequently shifts the melting/freezing temperature (T_0) of the bulk phase.[6,7]

The relationship between the melting temperature of the confined and bulk phases was first proposed in [6,7] and later refined to allow its application to both spherical and cylindrical geometries.[8] The resulting expression is given by Equation (1):

$$\Delta T_f = -\frac{K_{GT}}{x},$$

where K_{GT} is the Gibbs–Thomson constant (specific to the probe fluid) and x is the pore volume-to-surface ratio (V/S). Later developments of the method have enabled not only the determination of pore sizes but also the identification of the predominant pore geometry—spherical, cylindrical, or lamellar.[8]

The pore-size distribution can also be derived from the Gibbs–Thomson relation by assuming that the derivative of the relative pore volume filled with liquid, with respect to the pore radius r, is proportional to the derivative of the NMR signal intensity $I_{n\mathrm{DFMSE}}$ with respect to r. Since the experimental signal is measured as a function of temperature, r can be expressed in terms of T using

$$r = -\frac{\alpha K_{GT}}{T - T_{\mathrm{melting}}}. \qquad (2)$$

where α is a parameter that depends on the pore geometry. Thus,

$$\frac{dV}{dr} \propto \frac{dI_{n\mathrm{DFMSE}}}{dr} = \left|\frac{\partial I_{n\mathrm{DFMSE}}}{\partial T}\right| \left|\frac{\partial T}{\partial r}\right|, \qquad (3)$$

and, using the Gibbs–Thomson expression,

$$\left|\frac{\partial T}{\partial r}\right| = \left(\frac{T - T_{\mathrm{melting}}}{\alpha K_{GT}}\right)^2. \qquad (4)$$

Combining these terms yields

$$\frac{dV}{dr} \propto \left(\frac{T - T_{\mathrm{melting}}}{\alpha K_{GT}}\right)^2 \left|\frac{\partial I_{n\mathrm{DFMSE}}}{\partial T}\right|. \qquad (5)$$

The temperature derivative of $I_{n\mathrm{DFMSE}}$ may be obtained either numerically from discrete data or analytically from the derivative of a fitted sigmoidal function (e.g., a double-Boltzmann model), particularly suitable for controlled-pore systems with approximately Gaussian distributions of pore radii.

To observe these temperature variations, one must use a probe liquid with a known K_{GT} capable of penetrating the sample's pores while maintaining a fraction of bulk fluid.[9] An experimental technique sensitive to the melting or freezing differences between the confined and bulk phases is also required.

Among the available methods, Nuclear Magnetic Resonance (NMR) cryoporometry is one of the most widely used.[10,11] The classical approach employs the Hahn spin-echo sequence, which generates an echo that refocuses magnetic-field

inhomogeneities, susceptibility differences, and chemical-shift dispersion, but does not refocus ^1H–^1H dipolar couplings. Because dipolar couplings are strong in solids but averaged in liquids by fast molecular motion, the echo selectively detects the liquid component. With a suitable echo time $t_f = t_{\text{echo}}$, the rigid-phase signal is suppressed, and the temperature evolution of the remaining signal reveals the melting and freezing transitions that underpin cryoporometry.

However, since signal intensity also depends on factors such as the Curie law and instrumental drifts, it is customary to normalize the data using a reference liquid that does not undergo a phase transition within the studied temperature range. The normalized signal thus isolates effects due to pore confinement.

In this work, we employ an alternative acquisition scheme named Dipolar-Filtered Magic Sandwich Echo (DF-MSE) that provides the same phase discrimination while improving normalization and reducing experimental time.[12] The pulse sequence, shown in Figure S1a, begins with a Hahn-echo module that filters out rigid components, followed by a z-filter period of duration $t_z$that stores magnetization along the z-axis. A *mixed Magic Sandwich Echo* (mixed-MSE) block is then applied, refocusing both dipolar and inhomogeneity effects and generating an echo at time t_{MSE}. The acquired signal, represented schematically in blue, corresponds to the normalized intensity $I_{n\text{DFMSE}}$, which reports on the liquid fraction of the probe fluid.

The rationale for including the mixed-MSE block is that it allows simultaneous acquisition of a reference signal at a short echo time t_{ref} and the cryoporometry signal at t_f, both under identical experimental conditions. Because the reference echo represents the total sample magnetization (rigid + mobile), its intensity remains constant across the phase transition and can be used for normalization. This procedure effectively removes artifacts due to temperature-dependent signal variations, ensuring that changes in $I_{n\text{DFMSE}}$ reflect only the melting process.

To validate the experimental setup and ensure the proper calibration of the DF-MSE-based cryoporometry measurements, the method was first applied to a reference porous silica sample with a nominal pore diameter of 15 nm, using cyclohexane as the probe liquid. Figure S1b shows a typical temperature-dependent curve obtained for this reference sample. The sigmoidal increase of the normalized intensity corresponds to the progressive melting of the confined liquid, while the two distinct steps arise from

confined and bulk fractions, respectively. The melting-point depression between these transitions directly reflects the mean pore size according to the Gibbs–Thomson relation.

Figure S23c displays the pore-size distributions derived from this data. The black points correspond to the numerical derivative of the experimental $I_{n\mathrm{DFMSE}}$ curve, and the red line to the derivative of a smoothed double-Boltzmann fit. Both methods yield a narrow peak centered near 15 nm, in excellent agreement with the manufacturer's specification.

The experiment was conducted on a Bruker Minispec mq20 spectrometer operating at a ^{1}H Larmor frequency of 20 MHz ($B_0 \approx 0.5$T), using π/2 and π pulse lengths of 2.5 μs and 4.8 μs, respectively, and a dead time of 11.5 μs. The longitudinal relaxation time (T_1) was determined by standard inversion recovery, with recycling delays set to 3 T_1. Temperature calibration was performed using a thermocouple at the sample position, cross-checked against the Bruker BVT sensor, and validated by measuring the melting temperatures of cyclohexane, p-xylene, t-butanol, and menthol.

Overall, the DF-MSE sequence enables precise and reproducible NMR cryoporometry measurements.

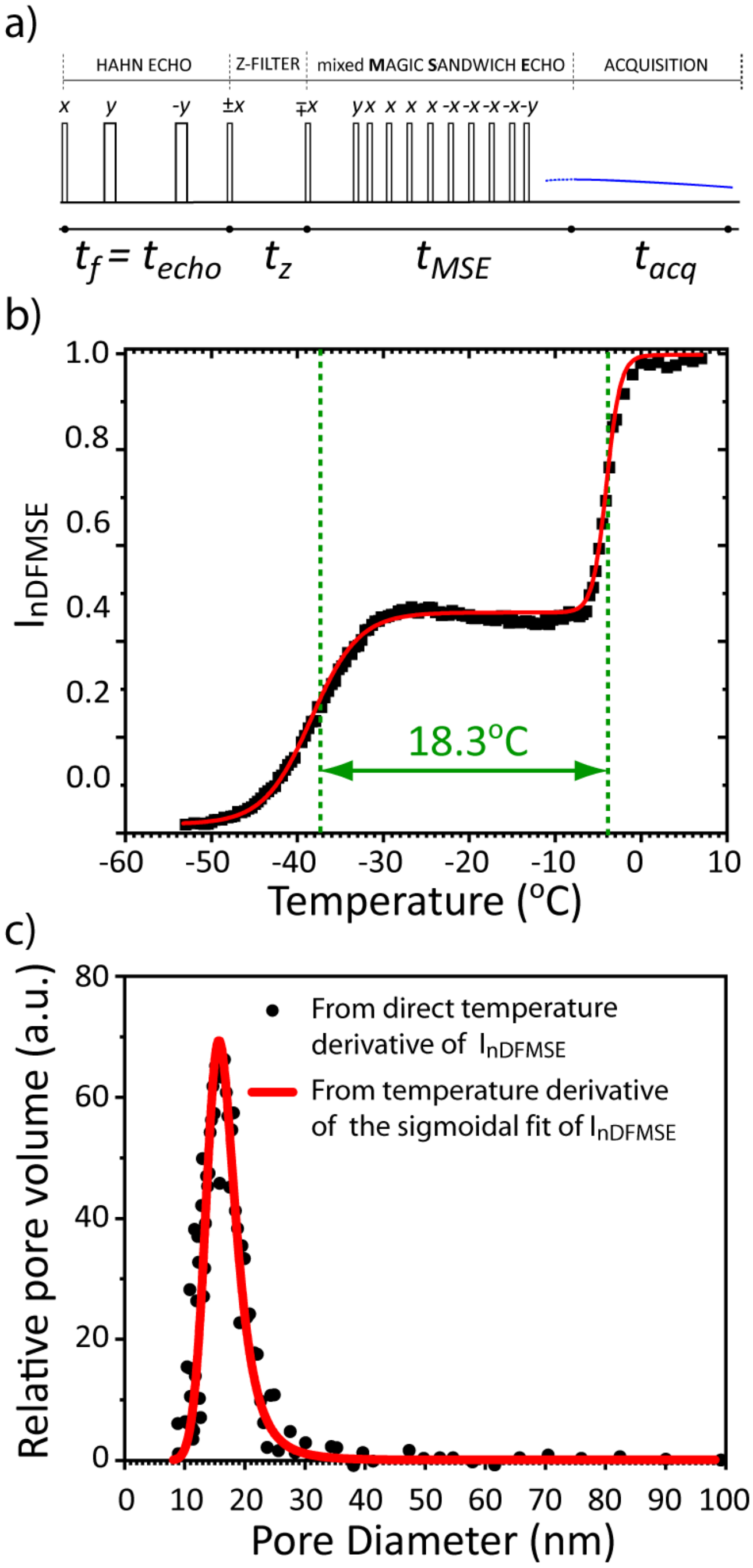

Figure S23: (a) Schematic representation of the Dipolar-Filtered Magic Sandwich Echo (DF-MSE) pulse sequence, showing its constituent blocks, timing parameters, and basic phase cycling of the pulses and receiver. Further details on the sequence implementation can be found in Reference [13]. (b) Normalized DF-MSE signal intensity I_{nDFMSE} as a function of temperature for a controlled-pore glass (CPG) sample with a nominal pore diameter of 15 nm, using cyclohexane as the probe liquid. (c) Pore-size distributions derived from the data in panel (b), obtained both directly from the temperature derivative of I_{nDFMSE} and from the derivative of the fitted sigmoidal Double Boltzmann function.

6. Periodic DFT/GIPAW NMR Calculations for Nitrogen-Site Assignment in dia-Cd(Im)$_2$

Crystallographic source

The initial structural model was obtained from the Cambridge Structural Database (CSD), curated by the Cambridge Crystallographic Data Centre (CCDC). The CIF downloaded for this work corresponds to CCDC deposition number 205574 and to the data DOI 10.5517/cc6wxfd. The associated crystallographic report is the group 12 imidazolato polymer study by Masciocchi and co-workers.[14] The phase considered here is Cd(im)$_2$, where *im* denotes imidazolate. The starting conventional cell is orthorhombic, space group Pbca (No. 61), with Z = 8. The resulting conventional cell contains 32 nitrogen atoms, which are the nuclei used for the NMR assignment analysis.

Table S4. Crystallographic information for the initial Cd(im)$_2$ model.

Source	CSD/CCDC CIF; CCDC 205574; data DOI 10.5517/cc6wxfd
Associated publication DOI	10.1039/B302840B
Chemical description	catena-(bis(μ$_2$-imidazolate-N,N′)-Cd(II)); Cd(im)$_2$
Formula weight	246.56 g mol^{-1}
Crystal system / space group	Orthorhombic / Pbca, No. 61
Unit-cell parameters	a = 14.6045 Å, b = 10.7385 Å, c = 9.9229 Å; α = β = γ = 90°
Cell volume / Z	1556.21 Å³ / 8
Number of nitrogen sites in conventional cell	32

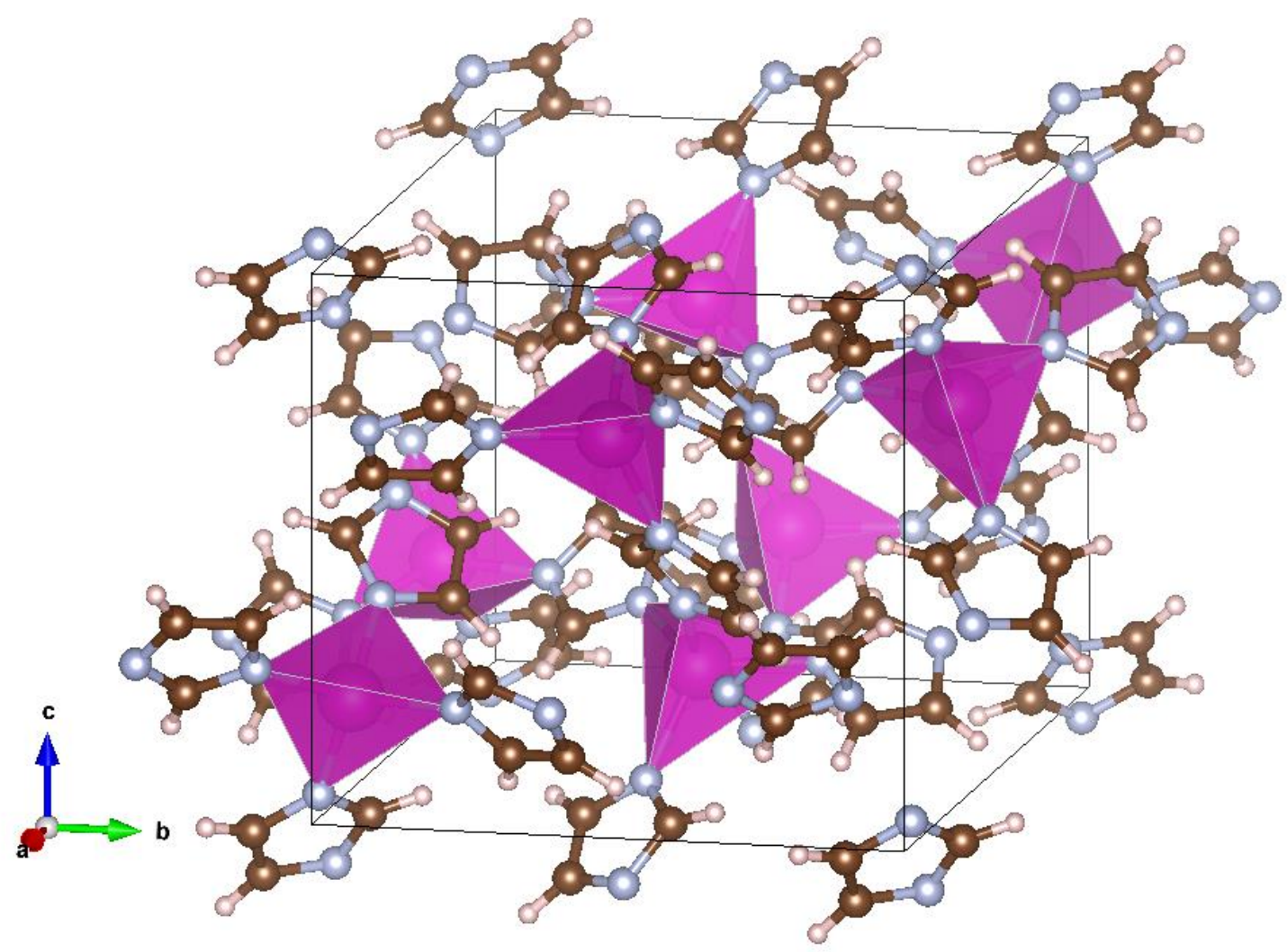

Figure S24. Crystallographic model used as the starting structure.

Since the model above is very complex, we will use a cell convention based on crystal symmetry and for illustrative purposes the following image should be used to understand the subsequent analysis in this Supporting Information.

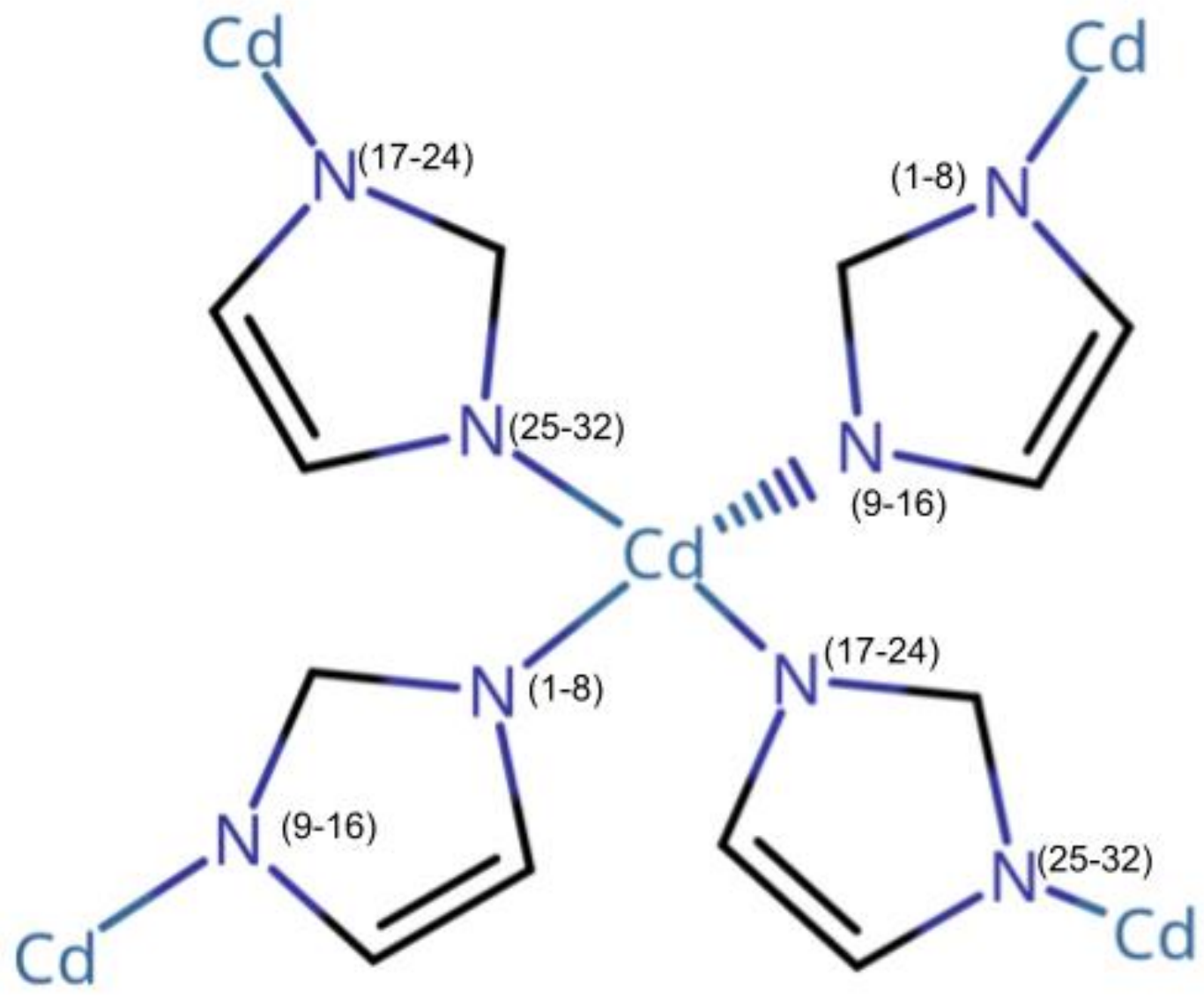

Figure S25. Crystallographic model used as reference for 15N atoms assignments according to the crystallographic symmetry.

Density Functional Theory calculations

All calculations were carried out with CASTEP 25.12 using periodic Kohn–Sham density-functional theory, plane-wave basis sets, and the PBE exchange-correlation functional.[15–18] Brillouin-zone integrations were performed with Monkhorst–Pack k-point meshes.[19] On-the-fly pseudopotentials generated by CASTEP 25.12 were used for all elements. Magnetic shielding and electric-field-gradient (EFG) tensors were calculated using the GIPAW formalism implemented in CASTEP.[20–25]

Table S5. Computational parameters used for DFT and GIPAW NMR calculations.

Code	CASTEP 25.12
Exchange-correlation functional	PBE
Pseudopotentials	CASTEP 25.12 on-the-fly pseudopotentials;
Spin treatment	Non-spin-polarized
Plane-wave cutoff	1200 eV
Electronic minimization tolerance	1.0×10^{-7}

A plane-wave kinetic-energy cutoff sweep was carried out from 600 to 1400 eV using the PBE functional and CASTEP on-the-fly pseudopotentials. The total energy was found to be converged at 1200 eV within the numerical tolerance adopted for this assignment, and 1200 eV was therefore used for subsequent geometry optimization and NMR calculations.

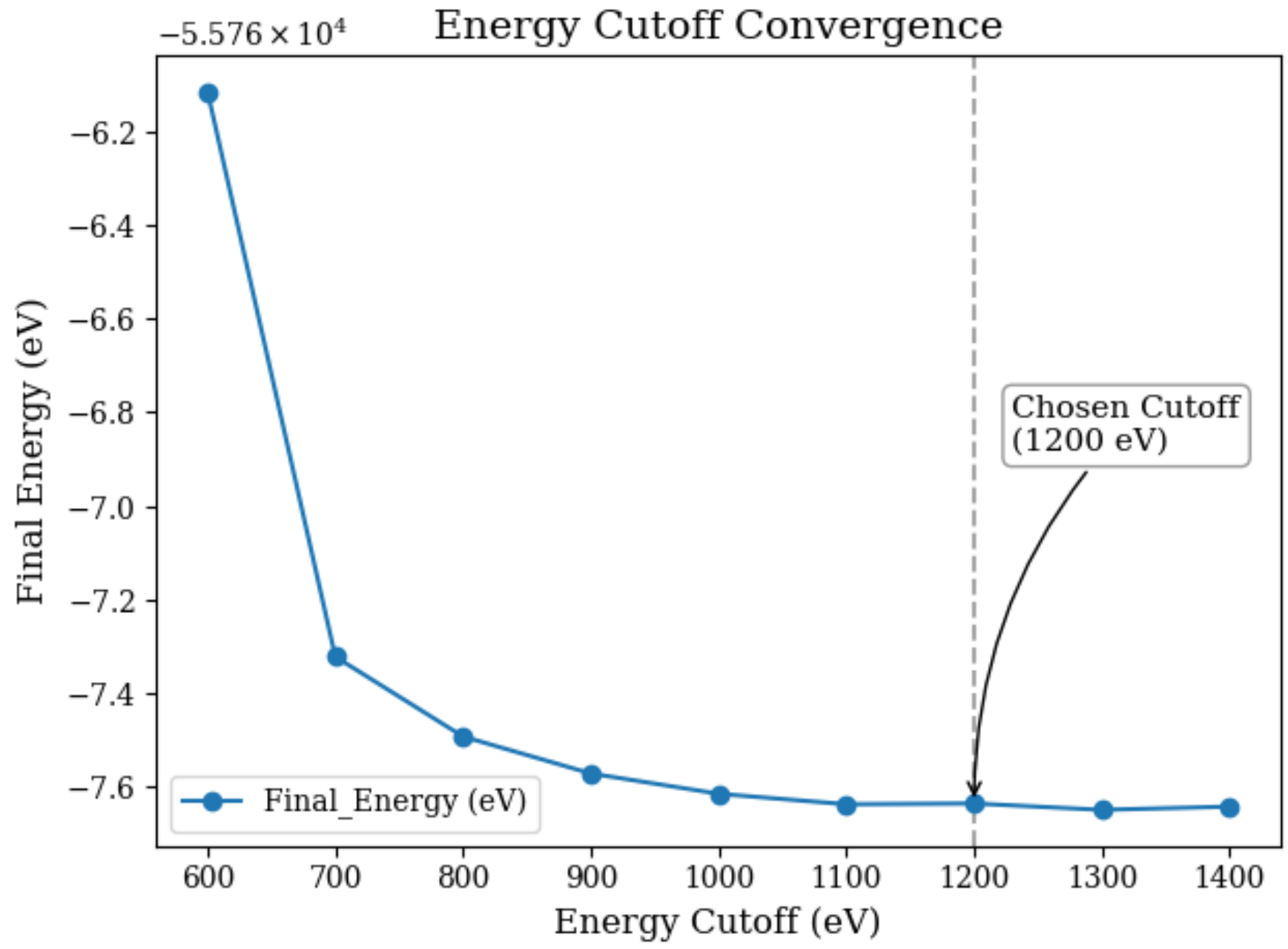

Figure S26. Plane-wave cutoff convergence from 600 to 1400 eV. Final Energy is the SCF converged energy for the whole unit cell.

After cutoff convergence, the Brillouin-zone sampling was tested by increasing the Monkhorst–Pack mesh. Single-point total-energy calculations indicated that a 4 × 5 × 5 mesh provides a practical convergence point for the present $Cd(im)_2$ conventional cell.

This mesh was selected as the production compromise between total-energy convergence and computational cost.

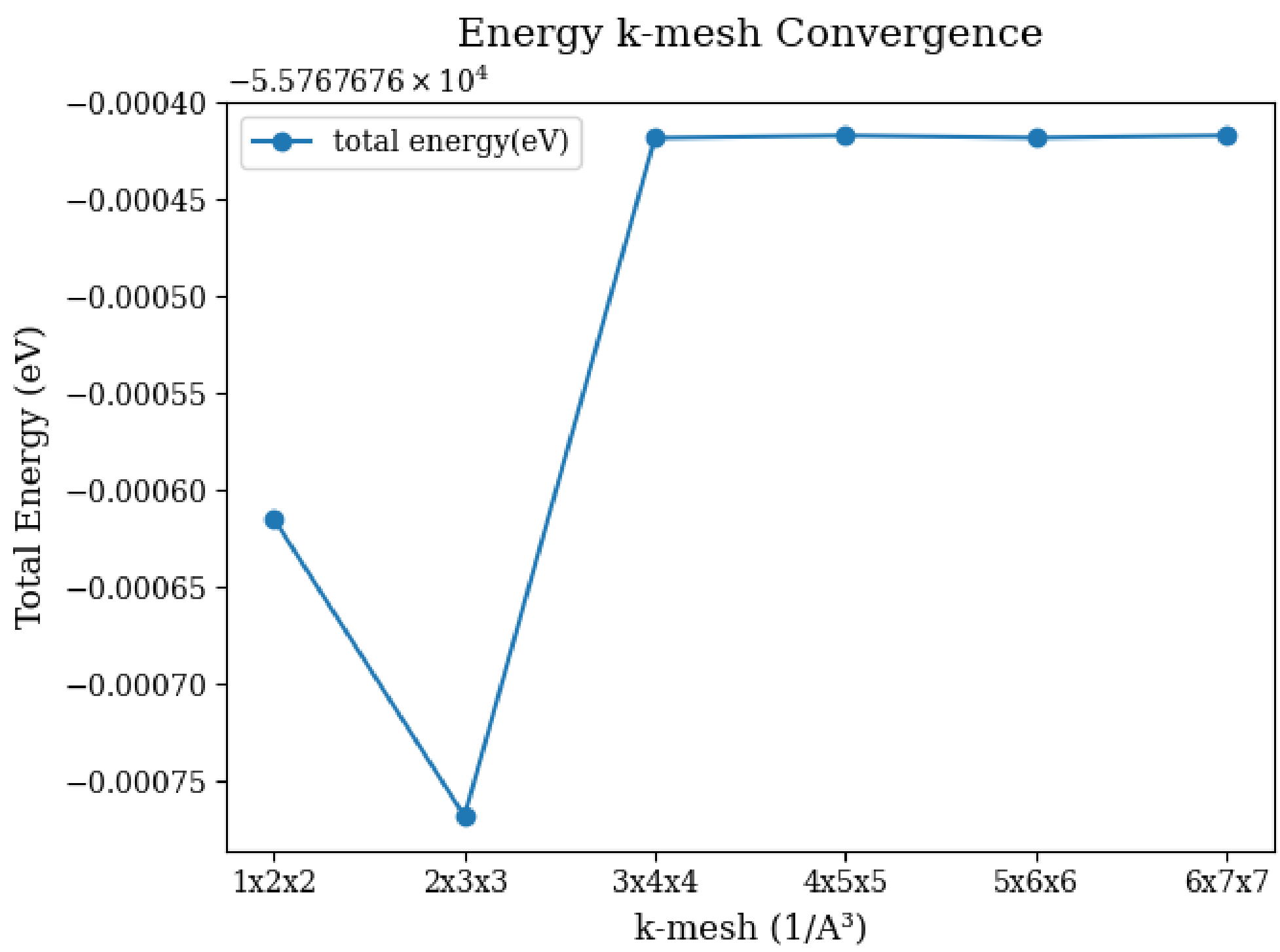

Figure S27. Total-energy convergence with Monkhorst–Pack k-point mesh. The mesh of $4 \times 5 \times 5$ shows the most stable choice for the single energy calculation.

A full NMR k-point convergence sequence on the final central-Γ mesh was not carried out because the corresponding GIPAW calculations are computationally expensive. At first, we tested at selected single-point and multi-point samples. This demonstrated the sensitivity of nitrogen NMR parameters to low Brillouin-zone sampling. The low k-point mesh of $3 \times 4 \times 4$ with an offset of 0.25 relative to Γ showed a stability of nitrogen magnetic shielding values related to other points as demonstrated by Table S3.

Table S6. Available nitrogen NMR response under selected k-point samplings. Values are group averages in ppm, based on the nitrogen similar relative sites around Cd.

Sampling label	Nk	Final energy / eV	N1–N8	N9–N16	N17–N24	N25–N32	Total N span
R single k-point	1	-55767.715331	11.65	20.90	3.42	19.24	17.48
S single k-point	1	-55767.721511	12.54	18.93	1.90	13.91	17.04
0.25 single k-point	1	-55767.677981	13.18	16.78	1.74	16.87	15.50
Offset 0.25 3 × 4 × 4	18	-55767.676663	13.18	16.76	1.73	16.88	15.15
Γ single k-point	1	-55767.637944	13.70	13.08	-0.15	14.13	14.30
Γ 2 × 3 × 3	9	-55767.677939	13.18	16.77	1.74	16.88	15.16
Γ 4 × 5 × 5	27	-55767.676770	13.18	16.76	1.73	16.88	15.15

The available NMR calculations show that single special-point sampling gives site-dependent nitrogen parameters that are not sufficiently stable for robust assignment. In contrast, the available multi-point samplings give a more reproducible separation pattern among the nitrogen groups. The multi-point tests in Table S3 give a total nitrogen span of approximately 15.15–15.16 ppm, close to the experimentally observed separation of approximately 15 ppm between the two broad nitrogen features. This agreement supports an assignment based on relative separations and multiplicities.

Geometry optimization protocols

Geometry optimization was treated as a separate source of uncertainty because NMR parameters, especially nitrogen shielding, are sensitive to local bonding geometry and hydrogen positions. Two optimization protocols were considered. The first completed protocol used the fixed experimental cell while allowing the atoms to relax. A second protocol keeps the unit-cell angles fixed and relaxes selected light atoms, specifically H and N. All the geometry relaxations were performed with a k-point mesh of 3 × 4 × 4. The NMR results were also calculated with a k-point mesh of 4 × 5 × 5.

The fixed-cell geometry optimization converged after 44 LBFGS iterations. The final convergence values were $\Delta E/ion = 4.29\times10^{-6}$ eV, $|F|_{max} = 4.38\times10^{-2}$ eVÅ^{-1}, and $|\Delta R|_{max}=9.42\times10^{-4}$Å, all below the selected convergence thresholds.

The H/N-relaxed geometry optimization was performed with a variable unit cell. This optimization reached full convergence with respect to the energy, maximum ionic force, maximum ionic displacement, and residual-stress criteria (Smax = 8.84×10^{-3} GPa, below the selected tolerance of 1.0×10^{-2} GPa). A third geometry-optimization protocol, in which only hydrogen positions were relaxed within the fixed experimental cell, was also carried out and is reported in Table S8. All three geometry-optimization protocols reported in this section (Tables S6, S7, S8) reached full convergence.

Fixed-cell optimized NMR results

The fixed-cell optimized calculation resolves four nitrogen environments, each with multiplicity eight. The calculated CS span is approximately 17.17 ppm, from the N17–N24 group at 9.16 ppm to the N1–N8 group at 26.23 ppm. This is of the same order as the experimental separation between the two broad nitrogen features. The two central calculated groups, N9–N16 and N25–N32, are separated by approximately 1.65 ppm; depending on the experimental linewidth, these environments may contribute to overlapping intensity within a broad component rather than to two fully resolved peaks. However, the presence of peaks around 16.88 ppm suggests that the final structure is not fully reliable to represent the magnetic shieldings for ^{15}N nuclei.

Table S7. Nitrogen groups from the fixed-cell optimized NMR calculation.

Group	Multiplicity	Mean CS / ppm	CS range / ppm	Internal spread / ppm
N1–N8	8	26.23	26.21–26.24	0.03
N9–N16	8	16.88	16.81–16.95	0.14
N17–N24	8	9.16	9.06–9.26	0.20
N25–N32	8	18.52	18.29–18.76	0.47

Nitrogen/Hydrogen optimized NMR results

The constrained H/N-relaxed structure gives a particularly clear nitrogen pattern. The N9-N16 and N25-N32 groups differ by only 0.63 ppm and are effectively degenerate under a 1 ppm practical resolution. The N1-N8 group lies 2.49-3.12 ppm from these two groups, and all three together span about 3.12 ppm. The N17-N24 group is separated from the N9-N16 group by 14.99 ppm and from the N25-N32 group by 14.36 ppm. Thus, the final H/N-relaxed calculation naturally gives one isolated nitrogen environment and a cluster of three environments.

Table S8. Nitrogen groups from the H/N-relaxed optimized NMR calculation.

Group	Multiplicity	Mean CS / ppm	CS range / ppm	Internal spread / ppm
N1–N8	8	11.32	11.31–11.33	0.02
N9–N16	8	14.24	14.23–14.24	0.01
N17–N24	8	-0.47	-0.47– -0.47	0.00
N25–N32	8	13.62	13.62–13.63	0.01

Table S9. Nitrogen groups from the fixed-cell, H-only-relaxed NMR calculation.

Group	Multiplicity	Mean CS/ppm	CS range/ppm	Internal spread/ppm
N1–N8	8	11.49	11.48–11.50	0.02
N9–N16	8	15.05	15.05–15.05	0.00
N17–N24	8	-0.33	–0.33 to –0.32	0.01
N25–N32	8	15.56	15.56–15.56	0.00

Interpretation for the nitrogen assignment

The experimental spectrum is described by two broad effective nitrogen features separated by approximately 15 ppm. Each broad feature can be decomposed into two overlapping components. The DFT calculations provide three assignment-relevant observations. First, the crystallographic conventional cell contains 32 nitrogen atoms that naturally group into four sets of eight sites. Second, the available multi-point NMR tests give a total nitrogen span close to 15 ppm, consistent with the experimental separation of the broad features. Third, three independent geometry-optimization protocols were evaluated, differing in which atoms and cell parameters were allowed to relax: all atoms within a fixed experimental cell (Table S6, span ≈ 17.2 ppm); hydrogen and nitrogen with a variable cell (Table S7, span ≈ 14.7 ppm); and hydrogen only within a fixed experimental cell (Table S8, span ≈ 15.9 ppm). All three protocols converge fully, and all three independently predict the same topology − one isolated nitrogen environment separated from a near-degenerate cluster of three. This agreement across three methodologically distinct, fully converged protocols is the basis for the assignment, rather than the result of any single calculation.

Validation of the ^{15}N referencing scale

The ^{15}N referencing scale (σ_{ref} = 220.7 ppm, main text) was validated using five reference compounds computed at the same level of theory as the framework (PBE, 1200 eV, GIPAW; Table S9): nitromethane, ammonium chloride[26], glycine[26], imidazole[26-28] and benzimidazole[29]. Each has an independently measured solid-state ^{15}N chemical shift converted into a common liquid-NH_3 chemical-shift scale[26-28]. Regressing the calculated absolute shielding against the experimental chemical shift ($\delta_{exp} = a + b\cdot\sigma_{calc}$, following Marín-Luna et al.[29]) gives, over all seven data points–two nitrogen sites each for imidazole and benzimidazole: $\delta = 223.7 - 1.012\cdot\sigma_{calc}$, $R^2 = 0.996$, residual standard error 8.8 ppm. Nitromethane is the only one of the five reference compounds for which the relaxed geometry is not directly comparable to the fixed-cell, periodic protocols used for the other four compounds and the framework — since it is an isolated, gas-phase-like molecule under full relaxation, whereas the others are relaxed within a fixed periodic cell. Excluding nitromethane, the regression over the remaining four compounds (six data points) gives $\delta = 218.7 - 0.966\cdot\sigma_{calc}$, $R^2 = 0.996$, residual standard error 6.8 ppm — a fit slope close to the theoretical value of −1 and a residual error comparable to Marín-Luna et al.'s own benchmark fits (11.6–19.8 ppm, 14 points), despite the smaller reference set here. Both regressions are consistent with the σref = 220.7 ppm constant used in the main text and reproduce the calculated nitrogen shifts of both Table S7 and Table S8 to within 3 ppm of the experimental 216 and 207 ppm resonances (Table S9). This constitutes a trend-level validation of the referencing scale: it demonstrates that the scale is internally consistent across several independently sourced reference compounds rather than an arbitrary choice. This supports the qualitative one-isolated/cluster-of-three assignment argument in the main text, which does not depend on matching absolute shift values beyond this level of precision.

Table S10. Calculated ^{15}N isotropic shielding for reference compounds (this work, same PBE/1200 eV/GIPAW protocol as the framework) and application of the referencing regression to the framework.

Reference Compound	Geometry protocol	σ_{calc} (ppm)
Nitromethane	isolated molecule, all atoms relaxed	−142.58
Ammonium chloride	fixed cell, all atoms relaxed (symmetry-frozen heavy atoms)	190.11
α-Glycine	fixed cell, H-only relaxed	193.46
Imidazole (N–H / =N–)	fixed cell, all atoms relaxed	42.63 / −21.29
Benzimidazole (N–H / =N–)	fixed cell, all atoms relaxed	58.07 / −11.20

7. SIMPSON Input file for simulation of REDOR curve

```
spinsys {
  # types of used nuclei
  channels 113Cd 1H
  # number of nuclei existent in the molecule
  # example for 3 spin system: nuclei 11B 11B 31P
  nuclei 113Cd 1H 1H 1H 1H
  # calculate dipole coupling in Hz --> must be negative
  # (e.g. for a known distance via "R" by Klaus Eichele)
  # specify nuclei (1 2) as noted above
  # three Euler angles: alpha beta gamma
  # for 3 spin system change beta
  # Cd-H
      dipole 1 2 1106   45.0   54.74   0
      dipole 1 3 1106  315.0  125.26   0
      dipole 1 4 1106  135.0  125.26   0
      dipole 1 5 1106  225.0   54.74   0
  # H-H
```

```
        dipole 2 3 -19187 270.0 135.00 0
        dipole 2 4 -19187 180.0 135.00 0
        dipole 2 5 -19187 225.0  90.00 0
        dipole 3 4 -19187 135.0  90.00 0
        dipole 3 5 -19187 180.0  45.00 0
        dipole 4 5 -19187 270.0  45.00 0
    }

par {
    # spin rate as in the experiment (in Hz)
    spin_rate        5000.0
    # number of points to be calculated
    np               8
    # repulsion scheme for averaging the gamma_angles
    crystal_file     rep100
    # number of equally distributed gamma_angles
    gamma_angles     720
    start_operator   I1x
    # next line indicates nuclei 1 as observed nuclei
    detect_operator  I1x
    # sweep width has to be multiple of spin_rate
    sw               spin_rate/2
    method           direct
    verbose          1111111
    # indicate which magnet was used (in Hz)
    proton_frequency        300000000
}

proc pulseq {} {
    global par
    maxdt 0.1
    offset 0 0
```

```
   #--------------------------------

##### define pulse lengths and power levels

      # pi pulse for 1st channel - see experiment - in micro-s
        set pi1 7.5
        # pi pulse for 2nd channel - see experiment - in micro-s
        set pi2 7.5
        # power for 1st channel - calculate: 1/(2*pi1) - in Hz!
        set powerf1 66666.66
        # power for 2nd channel - calculate: 1/(2*pi2) - in Hz!
        set powerf2 66666.66
      #-------------------
##### missing pi/2 pulse lengths and delays will be calculated

   set pi12   [expr $pi1/2]
   set pi22   [expr $pi2/2]
   set delay1 [expr 1000000.0/$par(spin_rate)/2.0-$pi22]
   set delay2 [expr 1000000.0/$par(spin_rate)/2.0-$pi2]
   set delay3 [expr 1000000.0/$par(spin_rate)/2.0-$pi12-$pi22]

##### store different blocks of sequences as shortcuts

   # start block (1 rotor period)
   reset
   delay $delay1
   pulse $pi2 0 0 $powerf2 x
   delay $delay2
   pulse $pi22 0 0 $powerf2 y
   store 1

   # middle block (1 rotor period)
```

```
    reset
    pulse $pi22  0 0 $powerf2 y
    delay $delay2
    pulse $pi2 0 0 $powerf2 x
    delay $delay2
    pulse $pi22 0 0 $powerf2  y
    store 2

    # 13C shift refocusing (2 rotor periods)
    reset
    pulse $pi22 0 0 $powerf2 y
    delay $delay2
    pulse $pi2 0 0 $powerf2 x
    delay $delay3
    pulse $pi1 $powerf1 0 0 0
    delay $delay3
    pulse $pi2 0 0 $powerf2 x
    delay $delay2
    pulse $pi22 0 0 $powerf2 y
    store 3

    # end block (1 rotor period)
    reset
    pulse $pi22 0 0 $powerf2 y
    delay $delay2
    pulse $pi2 0 0 $powerf2 x
    delay $delay1
    store 4

##### collect the first three points separately

    # reference - no dipolar recoupling
```

```
    reset
    acq

    # first point with recoupling (2 times 1 rotor period)
    reset
    delay $delay1
    pulse $pi2 0 0 $powerf2 x
    delay $delay3
    pulse $pi1 $powerf1 0 0 0
    delay $delay3
    pulse $pi2 0 0 $powerf2 x
    delay $delay1
    acq

    # second point with recoupling (2 times 2 rotor periods)
    reset
    prop 1
    prop 3
    prop 4
    acq

##### remaining points up to desired number of points

    for {set j 3} {$j < $par(np)} {incr j} {

      # succesively add a 2 propagator, store in 5
      reset
      if {$j > 3} {
        prop 5
      }
      prop 2
      store 5
```

```
    # final pulse sequence
    reset
    prop 1
    prop 5
    prop 3
    prop 5
    prop 4
    acq
  }
}
##### quick and dirty calculation of the FID
proc main {} {

 global par
   set f [fsimpson]
   fsave $f $par(name).fid
   funload $f
}
```

8. Data availability statement

CCDC 205574 contains the supplementary crystallographic data for the starting $Cd(im)_2$ structure. These data are available free of charge from the Cambridge Crystallographic Data Centre. The CASTEP input files, output files, magres files, and post-processing scripts used to generate figures can be available upon request.

9. Acknowledgement

Research carried out using the computational resources of the Center for Mathematical Sciences Applied to Industry (CeMEAI) funded by FAPESP (grant #2013/07375-0). This study was also financed, in part, by the São Paulo Research Foundation (FAPESP), Brasil. Process Number #2023/07712-8, #2022/02974-1, #2025/15689-1.

10. References

(1) Bennett, T. D.; Simoncic, P.; Moggach, S. A.; Gozzo, F.; Macchi, P.; Keen, D. A.; Tan, J.-C.; Cheetham, A. K. Reversible pressure-induced amorphization of a zeolitic imidazolate framework (ZIF-4). *Chem. Commun.* **2011**, *47* (28), 7983–7985. https://doi.org/10.1039/C1CC11985K

(2) Mohan, S.; Sundaraganesan, N.; Mink, J. FTIR and Raman studies on benzimidazole. *Spectrochim. Acta A Mol. Biomol. Spectrosc.* **1991**, *47* (8), 1111–1115. https://doi.org/10.1016/0584-8539(91)80042-H

(3) van Meerten, S. G. J.; Franssen, W. M. J.; Kentgens, A. P. M. ssNake: A cross-platform open-source NMR data processing and fitting application. *J. Magn. Reson.* **2019**, *301*, 56–66. https://doi.org/10.1016/j.jmr.2019.02.006

(4) Wiench, J. W.; Bronnimann, C. E.; Lin, V. S.-Y.; Pruski, M. Chemical shift correlation NMR spectroscopy with indirect detection in fast rotating solids: Studies of organically functionalized mesoporous silicas. *J. Am. Chem. Soc.* **2007**, *129* (40), 12076–12077. https://doi.org/10.1021/ja074746+

(5) Mafra, L.; Siegel, R.; Fernandez, C.; Schneider, D.; Aussenac, F.; Rocha, J. High-resolution ^{1}H homonuclear dipolar recoupling NMR spectra of biological solids at MAS rates up to 67 kHz. *J. Magn. Reson.* **2009**, *199* (1), 111–114. https://doi.org/10.1016/j.jmr.2009.04.004

(6) Khokhlov, A.; Valiullin, R.; Kärger, J.; Steinbach, F.; Feldhoff, A. Freezing and melting transitions of liquids in mesopores with ink-bottle geometry. *New J. Phys.* **2007**, *9* (8), 272. https://doi.org/10.1088/1367-2630/9/8/272

(7) Denoyel, R.; Pellenq, R. J.-M. Simple phenomenological models for phase transitions in a confined geometry. 1. Melting and solidification in a cylindrical pore. *Langmuir* **2002**, *18* (7), 2710–2716. https://doi.org/10.1021/la015607k

(8) Petrov, O. V.; Furó, I. Curvature-dependent metastability of the solid phase and the freezing–melting hysteresis in pores. *Phys. Rev. E* **2006**, *73* (1), 011608. https://doi.org/10.1103/PhysRevE.73.011608

(9) Rottreau, T. J.; Parlett, C. M. A.; Lee, A. F.; Evans, R. Extending the range of liquids available for NMR cryoporometry studies of porous materials. *Microporous Mesoporous Mater.* **2019**, *274*, 198–202. https://doi.org/10.1016/j.micromeso.2018.08.007

(10) Mitchell, J.; Webber, J. B. W.; Strange, J. H. Nuclear magnetic resonance cryoporometry. *Phys. Rep.* **2008**, *461* (1), 1–36. https://doi.org/10.1016/j.physrep.2008.02.001

(11) Petrov, O. V.; Furó, I. NMR cryoporometry: Principles, applications and potential. *Prog. Nucl. Magn. Reson. Spectrosc.* **2009**, *54* (2), 97–122. https://doi.org/10.1016/j.pnmrs.2008.06.001

(12) Filgueiras, J. G.; Trebbi, B.; Garcia, R. H. S.; Azevedo, E. R. Dipolar filtered magic-sandwich-echoes as a tool for probing molecular motions using time-domain NMR. *J. Magn. Reson.* **2017**, *285*, 47–54. https://doi.org/10.1016/j.jmr.2017.10.008

(13) Trebbi, B.; Filgueiras, J. G.; Garcia, R. H. dos S.; Murback, R. P.; Oliveira, M.; deAzevedo, E. R. Dipolar-Filtered Magic Sandwich Echo as an Alternative Method for NMR Cryoporometry. *J. Magn. Reson.* **2026**, *388*, 108075. https://doi.org/10.1016/j.jmr.2026.108075

(14) Masciocchi, N.; Ardizzoia, G. A.; Brenna, S.; Castelli, F.; Galli, S.; Maspero, A.; Sironi, A. Synthesis and ab-initio XRPD structure of group 12 imidazolato polymers. *Chem. Commun.* **2003**, No. 16, 2018–2019. https://doi.org/10.1039/B302840B

(15) Hohenberg, P.; Kohn, W. Inhomogeneous electron gas. *Phys. Rev.* **1964**, *136* (3B), B864–B871. https://doi.org/10.1103/PhysRev.136.B864

(16) Kohn, W.; Sham, L. J. Self-consistent equations including exchange and correlation effects. *Phys. Rev.* **1965**, *140* (4A), A1133–A1138. https://doi.org/10.1103/PhysRev.140.A1133

(17) Clark, S. J.; Segall, M. D.; Pickard, C. J.; Hasnip, P. J.; Probert, M. J.; Refson, K.; Payne, M. C. First principles methods using CASTEP. *Z. Kristallogr.* **2005**, *220* (5-6), 567–570. https://doi.org/10.1524/zkri.220.5.567.65075

(18) Perdew, J. P.; Burke, K.; Ernzerhof, M. Generalized gradient approximation made simple. *Phys. Rev. Lett.* **1996**, *77* (18), 3865–3868. https://doi.org/10.1103/PhysRevLett.77.3865

(19) Monkhorst, H. J.; Pack, J. D. Special points for Brillouin-zone integrations. *Phys. Rev. B* **1976**, *13* (12), 5188–5192. https://doi.org/10.1103/PhysRevB.13.5188

(20) Payne, M. C.; Teter, M. P.; Allan, D. C.; Arias, T. A.; Joannopoulos, J. D. Iterative minimization techniques for ab initio total-energy calculations: Molecular dynamics and conjugate gradients. *Rev. Mod. Phys.* **1992**, *64* (4), 1045–1097. https://doi.org/10.1103/RevModPhys.64.1045

(21) Vanderbilt, D. Soft self-consistent pseudopotentials in a generalized eigenvalue formalism. *Phys. Rev. B* **1990**, *41* (11), 7892–7895. https://doi.org/10.1103/PhysRevB.41.7892

(22) Pickard, C. J.; Mauri, F. All-electron magnetic response with pseudopotentials: NMR chemical shifts. *Phys. Rev. B* **2001**, *63* (24), 245101. https://doi.org/10.1103/PhysRevB.63.245101

(23) Yates, J. R.; Pickard, C. J.; Mauri, F. Calculation of NMR chemical shifts for extended systems using ultrasoft pseudopotentials. *Phys. Rev. B* **2007**, *76* (2), 024401. https://doi.org/10.1103/PhysRevB.76.024401

(24) Profeta, M.; Mauri, F.; Pickard, C. J. Accurate first principles prediction of ^{17}O NMR parameters in SiO_2: Assignment of the zeolite ferrierite spectrum. *J. Am. Chem. Soc.* **2003**, *125* (2), 541–548. https://doi.org/10.1021/ja027124r

(25) Bonhomme, C.; Gervais, C.; Babonneau, F.; Coelho, C.; Pourpoint, F.; Azaïs, T.; Ashbrook, S. E.; Griffin, J. M.; Yates, J. R.; Mauri, F.; Pickard, C. J. First-principles calculation of NMR parameters using the Gauge Including Projector Augmented Wave method: A chemist's point of view. *Chem. Rev.* **2012**, *112* (11), 5733–5779. https://doi.org/10.1021/cr300108a

(26) Bertani, P.; Raya, J.; Bechinger, B. ^{15}N chemical shift referencing in solid state NMR. *Solid State Nucl. Magn. Reson.* **2014**, *61–62*, 15–18. https://doi.org/10.1016/j.ssnmr.2014.03.003

(27) Hickman, B. S.; Mascal, M.; Titman, J. J.; Wood, I. G. Protonic conduction in imidazole: A solid-state ^{15}N NMR study. *J. Am. Chem. Soc.* **1999**, *121* (49), 11486–11490. https://doi.org/10.1021/ja9914011

(28) Solum, M. S.; Altmann, K. L.; Strohmeier, M.; Berges, D. A.; Zhang, Y.; Facelli, J. C.; Pugmire, R. J.; Grant, D. M. ^{15}N chemical shift principal values in nitrogen heterocycles. *J. Am. Chem. Soc.* **1997**, *119* (41), 9804–9809. https://doi.org/10.1021/ja964135+

(29) Shkrob, I. A.; Marin, T. W.; Chemerisov, S. D.; Hatcher, J. L.; Wishart, J. F. Radiation induced redox reactions and fragmentation of constituent ions in ionic liquids. 2. Imidazolium cations. *J. Phys. Chem. B* **2011**, *115* (14), 3889–3902. https://doi.org/10.1021/jp200305b
